# ﻿Taxonomic notes of jumping spiders (Araneae, Salticidae) from Guangxi, Hainan, Sichuan, Xizang and Yunnan, China

**DOI:** 10.3897/zookeys.1221.135640

**Published:** 2024-12-17

**Authors:** Cheng Wang, Xiaoqi Mi, Shuqiang Li, Xiang Xu

**Affiliations:** 1 Guizhou Provincial Key Laboratory for Biodiversity Conservation and Utilization in the Fanjing Mountain Region, Tongren University, Tongren, Guizhou 554300, China Tongren University Tongren China; 2 Institute of Zoology, Chinese Academy of Sciences, Beijing 100101, China Chinese Academy of Sciences Beijing China; 3 University of Chinese Academy of Sciences, Beijing, China University of Chinese Academy of Sciences Beijing China; 4 College of Life Science, Hunan Normal University, Changsha 410081, Hunan, China Hunan Normal University Changsha China

**Keywords:** Morphology, new combination, new species, synonym, taxonomy

## Abstract

Twenty-one new species of jumping spiders from five provinces of South China are described: *Cheliceroidesjinxini***sp. nov.** (♂), *Dendroiciusqiong***sp. nov.** (♂♀), *Iciusdeergong***sp. nov.** (♂♀), *Iruraqiuhangi***sp. nov.** (♂♀), *I.yarlungzangbo***sp. nov.** (♂♀), *Mintoniashiwandashan***sp. nov.** (♂), *Myrmarachnekuan***sp. nov.** (♂♀), *Nandiciusxiefengi***sp. nov.** (♂♀), *Pancoriusmedog***sp. nov.** (♀), *P.yingjiang***sp. nov.** (♂♀), *Piranthusmaddisoni***sp. nov.** (♂♀), *Simaethahainan***sp. nov.** (♂♀), *Stertiniuslhoba***sp. nov.** (♂♀), *Synagelideskongmingi***sp. nov.** (♂♀), *S.xuandei***sp. nov.** (♂♀), *S.yunchangi***sp. nov.** (♂♀), *S.yidei***sp. nov.** (♂), *S.zilongi***sp. nov.** (♂♀), *Yaginumaelladaweishan***sp. nov.** (♂♀), *Y.moinba***sp. nov.** (♂♀), and *Y.pingbian***sp. nov.** (♂♀). *Nepalicius* Prószyński, 2016, **syn. nov.** is proposed as a junior synonym of *Okinawicius* Prószyński, 2016. Three new combinations are proposed: *O.nepalicus* (Andreeva, Hęciak & Prószyński, 1984), **comb. nov.** and *O.seychellensis* (Wanless, 1984), **comb. nov.** transferred from *Nepalicius*, and *O.daoxianensis* (Peng, Gong & Kim, 2000), **comb. nov.** transferred from *Philaeus* Thorell, 1869. The unknown females of *O.nepalicus*, *Padillothoraxexilis* (Cao & Li, 2016) and *Silerhanoicus* Prószyński, 1985 are described for the first time. Distribution maps of the studied specimens are also provided.

## ﻿Introduction

Salticidae, the largest family in Araneae, currently contains 6702 extant species in 685 genera distributed worldwide (WSC 2024). The taxonomic study of the family from China has a relatively long history, but it has been in rapid development until recently four decades (WSC 2024). A series of continuous taxonomic studies from some tropical areas, such as Xishuangbanna, Yunnan, and Hainan provinces, and taxonomic studies and revisions on several genera of Chrysillini Simon, 1901 and Euophryini Simon, 1901 have significantly increased our knowledge (Wang et al. 2023; WSC 2024). Peng et al. (1993) and Peng (2020) also conducted two comprehensive taxonomic works.

To date, at least 773 nominal species (including the species described in the present work) under 144 genera have been recorded in China (Metzner 2024; WSC 2024), and the species number far exceeds the figure for nearby countries such as India (349), Vietnam (180), and even the most species-rich countries worldwide, such as Australia (537) and Brazil (714) (Metzner 2024). However, the taxonomic study of the family from China remains unsatisfactory because most regions have not been adequately studied, even some hot spot provinces such as Hainan and Yunnan, where new species or records are continuously being discovered. Moreover, it is also limited by high rates of poorly studied species that cannot be precisely identified or known only from a single sex, and quite a few genera cannot be adequately defined. As stated by Li (2020), Luo and Li (2024), Zhang et al. (2023), Lu et al. (2022), the true diversity of Chinese spiders could reach very high.

In our recent examination of jumping spiders from the five provinces of south China, more than twenty species were recognized as new to science, and the unknown females of three species were found. The goals of the present work are to (re)describe those species (all are the members of the subfamily Salticinae Blackwall, 1841 except *Mintoniashiwandashan* sp. nov. belongs to the subfamily Spartaeinae Wanless, 1984) and propose a synonym and three new combinations.

## ﻿Materials and methods

Specimens were collected by beating shrubs or sieving leaflitter and preserved in 80% or absolute ethanol. They are deposited in the Institute of Zoology, Chinese Academy of Sciences in Beijing (**IZCAS**), China, and Tongren University (**TRU**) in Tongren, China. Methods followed Wang et al. (2024).

All measurements are given in millimeters. Leg measurements are given as: total length (femur, patella, tibia, metatarsus, tarsus). References to figures in the cited papers are listed in lowercase type (fig. or figs), and figures in this paper are noted with an initial capital (Fig. or Figs). Abbreviations used in the text and figures are as follows: **AERW** anterior eye row width; **AME** anterior median eye; **ALE** anterior lateral eye; **AG** accessory gland; **AL** anterior tegular lobe; **AR** atrial ridge; **AS** anterior chamber of spermatheca; **At** atrium; **BTA** baso-retrolateral tibial apophysis; **CD** copulatory duct; **CF** cymbial flange; **CO** copulatory opening; **CP** cymbial process; **CR** prolateral cymibal ridged portion; **DCA** dorsal cymbial apophysis; **DCE** dorsal cymbial extension; **DCP** dorsal cymbial process; **DD** dorsal denticle of retrolateral tibial apophysis; **DTA** dorsal tibial apophysis; **DTP** dorsal tibial process; **E** embolus; **EFL** eye field length; **F** epigynal fold; **FD** fertilization duct; **H** epigynal hood; **JS** junction duct of spermatheca; **MA** median apophysis; **MTP** membranous tegular peak; **PCA** prolateral cymbial apophysis; **PERW** posterior eye row width; **PB** patellar bump of male palp; **PL** posterior tegular lobe; **PME** posterior median eye; **PCA** prolateral cymbial apophysis; **PFA** prolateral femoral apophysis; **PLE** posterior lateral eye; **PS** posterior chamber of spermatheca; **PTA** prolateral tibial apophysis; **PTgA** prolateral tegular apophysis; **RCA** retrolateral cymbial apophysis; **RTA** retrolateral tibial apophysis; **S** spermatheca; **Se** septum; **SD** sperm duct; **TB** tegular bump; **TL** tegular lobe; **UI** U-shaped incision of embolic disc; **VTA** ventral tibial apophysis; **VTP** ventral tibial process.

## ﻿Results

### ﻿Family Salticidae Blackwall, 1841

#### ﻿Genus Cheliceroides

Taxon classificationAnimaliaAraneaeSalticidae

Żabka, 1985

B9906D7A-87DB-5CBE-878B-012C7A250856

##### Type species.

*Cheliceroideslongipalpis* Żabka, 1985; type locality Cuc Phuoug, Vietnam.

##### Comments.

This monotypic genus was considered a synonym of *Colopsus* Simon, 1902 by Logunov (2021) but was recently revalidated by Lin et al. (2024a). It is placed in the tribe Hasariini Simon, 1903 by Maddison (2015), but that has been confirmed as doubtful, and its phylogenetic placement remains uncertain (Lin et al. 2024a).

#### ﻿ Cheliceroides jinxini sp. nov.

Taxon classificationAnimaliaAraneaeSalticidae

938E4566-54D5-59FA-AB7E-65E45A229BBD

https://zoobank.org/AF3ECB2E-C8A4-444E-914F-96A97EDDFE42

Figs 1 , 2 , 47

##### Type material.

***Holotype*** ♂ (TRU-JS 0729), China: • Yunnan Province, Menghai County, Bameng Village (22°08.1'N, 100°31.56'E, ca 2030 m), 1.VII.2023, J.X. Liu et al. leg.

##### Etymology.

The specific name is a patronym in honor of the collector; noun (name) in the genitive case.

##### Diagnosis.

**Figure 1.**
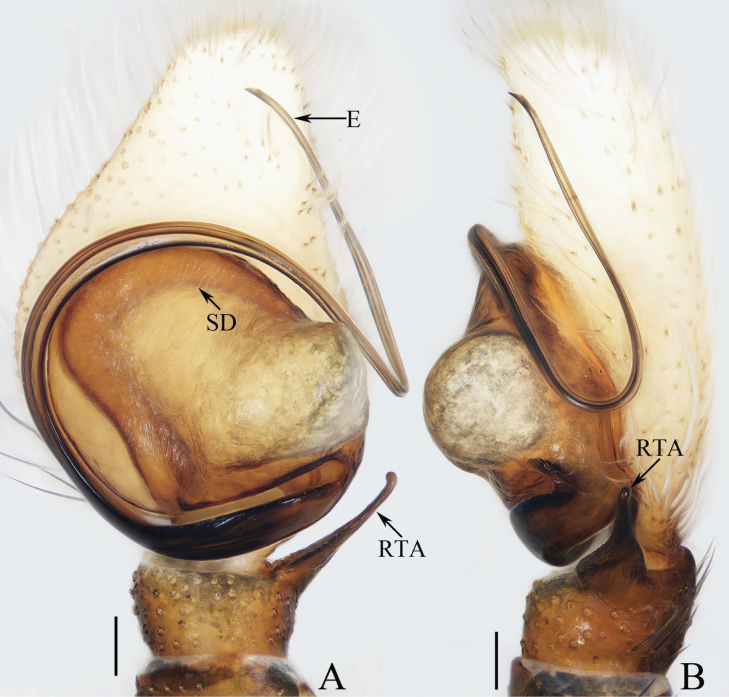
Male palp of *Cheliceroidesjinxini* sp. nov., holotype **A** ventral **B** retrolateral. Abbreviations: E embolus; RTA retrolateral tibial apophysis; SD sperm duct. Scale bars: 0.1 mm.

**Figure 2.**
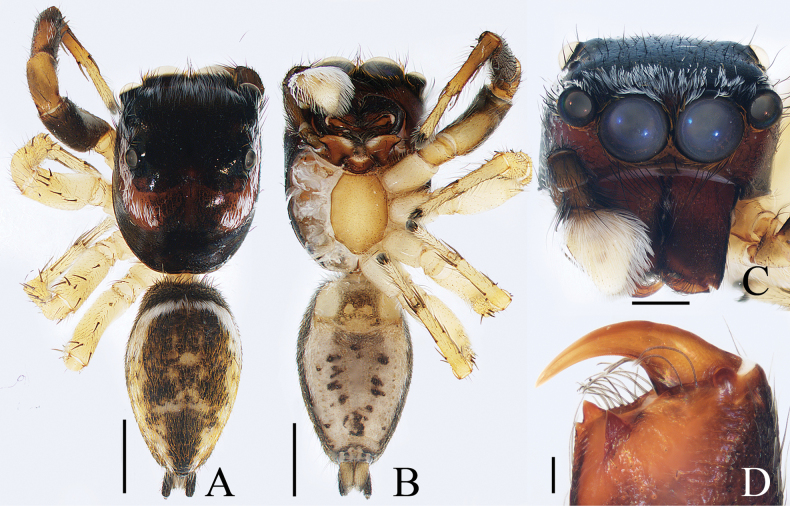
*Cheliceroidesjinxini* sp. nov., holotype **A** habitus, dorsal **B** ditto, ventral **C** carapace, frontal **D** chelicera, posterior. Scale bars: 1.0 mm (**A, B**); 0.5 mm (**C**); 0.1 mm (**D**).

*Cheliceroidesjinxini* sp. nov. resembles *C.longipalpis* in habitus and palpal structure, but can be distinguished by the following: 1) presence of a raised tegular portion (Fig. 1A, B) vs absent (Lin L. et al. 2024a: figs 9–11, 16); 2) male palpal tibia ~ 1/5 of cymbial length (Fig. 1A, B) vs approx. as long as cymbium (Lin et al. 2024a: figs 9–11, 16); 3) embolus (E) originating at ca 4 o’clock position (Fig. 1A, B) vs ca 2 o’clock position (Lin et al. 2024a: fig. 10); and 4) chelicerae unmodified, and presence of one retromarginal tooth (Fig. 2D) vs modified, and two retromarginal teeth (Logunov 2021: figs 2, 4).

##### Description.

**Male** (Figs 1, 2). Total length 5.40. Carapace 2.60 long, 2.04 wide. Abdomen 2.68 long, 1.64 wide. Eye sizes and interdistances: AME 0.64, ALE 0.36, PLE 0.30, AERW 1.96, PERW 1.80, EFL 1.24. Legs: I 5.99 (1.75, 1.08, 1.45, 1.08, 0.63), II 5.12 (1.58, 0.95, 1.18, 0.88, 0.53), III 5.67 (1.83, 0.88, 1.20, 1.13, 0.63), IV 5.89 (1.83, 0.80, 1.28, 1.35, 0.63). Carapace dark except anterior half of thoracic part red-brown, covered with dense dark and white setae, with clusters of bilateral, dense white scales. Chelicerae red-brown, with two promarginal teeth and one retromarginal tooth. Legs overall yellow except femora I dark brown, patellae, tibiae, and metatarsi I yellow-brown, spiny. Dorsum of abdomen yellow laterally, with anterior, transverse, arc-shaped setal stripes, and central, longitudinal, dark patch; venter pale brown, with dark spots.

***Palp*** (Fig. 1A, B): femur length/width ratio ca 3.32; patella slightly wider than long; tibia short, ~ 2× wider than long in ventral view; retrolateral tibial apophysis (RTA) broadened into sub-quadrangular portion at base, then tapered to blunt end slightly curved inward; cymbium flat, ~ 1.5× longer than wide in ventral view; tegulum almost round, with swollen retrolateral portion; embolus (E) long, arising at ca 4 o’clock position, extending circularly (ca 340°) along tegulum before strongly curving 180°, then antero-prolaterally extending into acutely pointed tip at apex of cymbium.

**Female**. Unknown.

##### Distribution.

Known only from the type locality in Yunnan, China (Fig. 47).

##### Comments.

The new species is considered a member of the genus because it shares a series of characters with *C.longipalpis*, such as the similar habitus, pattern, and long and whip-like embolus. However it is also obviously different from the latter by the unmodified chelicerae with one retromarginal tooth (vs modified, elongated chelicerae with two retromarginal teeth; Logunov 2021: figs 3, 9), the C-shaped sperm duct (vs S-shaped; Logunov 2021: fig. 5), and only the cymbium bears dense white setae (vs all segments except coxae and femora are densely covered with white setae; Logunov 2021: figs 2, 4). Therefore, the generic position of this species remains uncertain. Discovering its unknown female and obtaining enough molecular evidence could be helpful in confirming this issue.

#### ﻿Genus Dendroicius

Taxon classificationAnimaliaAraneaeSalticidae

Lin & Li, 2020

5D0C1179-375C-52C1-B933-3378457AB8D0

##### Type species.

*Dendroiciushotaruae* Lin & Li, 2020; type locality Menglun Township, Mengla County, Yunnan, China.

##### Comments.

This monotypic genus was not placed in any of the subfamilies and tribes of Salticidae. Judging from the conformation of the male palp, and particularly in having a tegular bump, it belongs to Chrysillini Simon, 1901. It is only known from the original description (WSC 2024).

#### ﻿ Dendroicius qiong sp. nov.

Taxon classificationAnimaliaAraneaeSalticidae

8BDF5D8C-7AB7-5304-ACFB-A25B26F4DBD4

https://zoobank.org/A1C42A54-F6D7-4CCF-8BA0-F916BB5544D5

Figs 3 , 4 , 47

##### Type material.

***Holotype*** ♂ (TRU-JS 0730), China: • Hainan Province, Baoting Li and Miao Autonomous County, Maogan Township, 124 road (18°39.32'N, 109°32.45'E, ca 530 m), 4.VIII.2023, C. Wang et al. leg. ***Paratypes*** • 1 ♂ (TRU-JS 0731), same data as for holotype; • 2 ♀ (TRU-JS 0823, 0824), same locality as for holotype, 4.IX.2024, C. Wang and S. K. Li leg.

##### Etymology.

The specific name refers to the short name of type locality (Hainan Province); noun in apposition.

##### Diagnosis.

**Figure 3.**
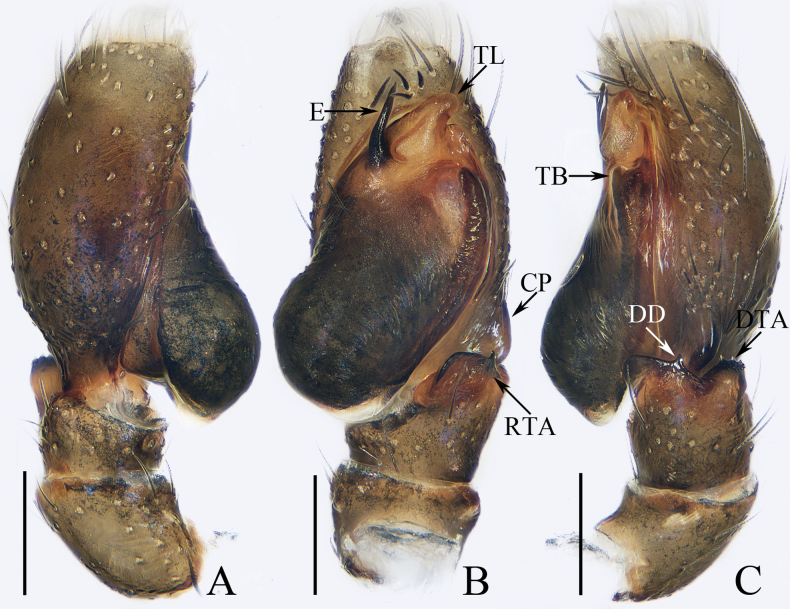
Male palp of *Dendroiciusqiong* sp. nov., holotype **A** palp, prolateral **B** ditto, ventral **C** ditto, retrolateral. Abbreviations: CP cymbial process; DD dorsal denticle of retrolateral tibial apophysis; DTA dorsal tibial apophysis; E embolus; RTA retrolateral tibial apophysis; TB tegular bump; TL tegular lobe. Scale bars: 0.1 mm.

**Figure 4.**
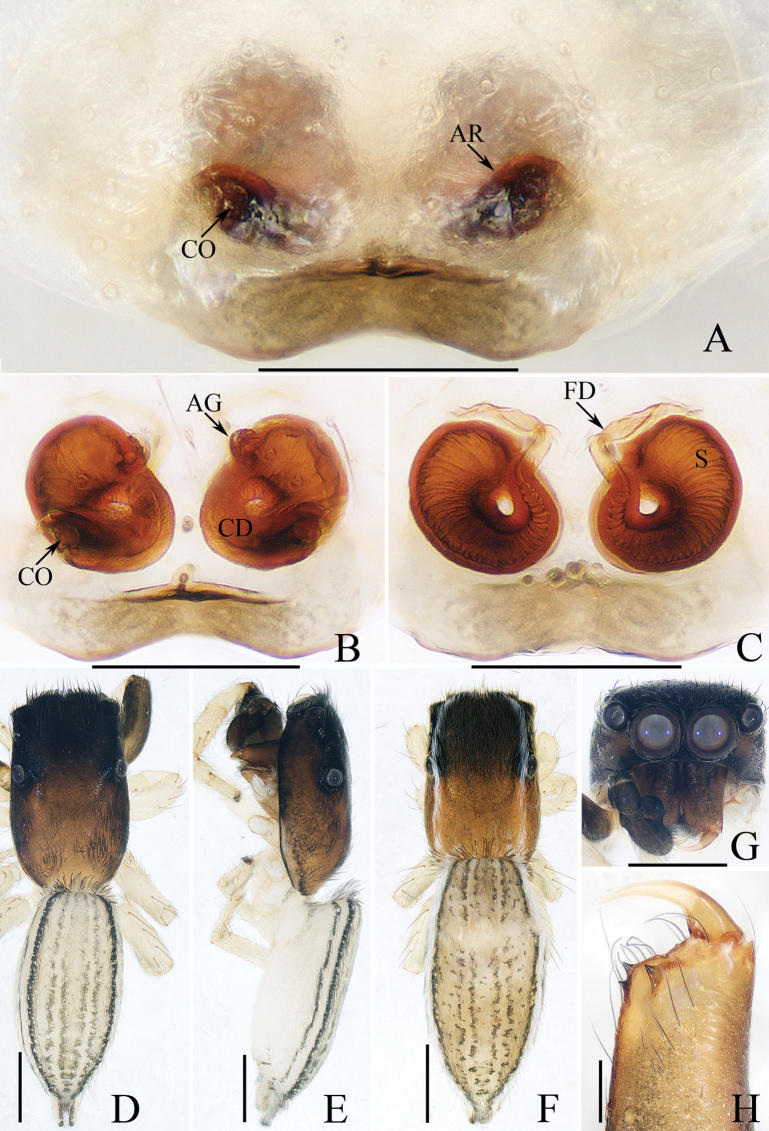
*Dendroiciusqiong* sp. nov. **D, E, G, H** male holotype and **A–C, F** female paratype (TRU-JS 0823) **A, B** epigyne, ventral **C** vulva, dorsal **D, F** habitus, dorsal **E** ditto, lateral **G** carapace, frontal **H** chelicera, posterior. Abbreviations: AG accessory gland; AR atrial ridge; CD copulatory duct; CO copulatory opening; FD fertilization duct; S spermatheca. Scale bars: 0.1 mm (**A–C, H**); 0.5 mm (**D–G**).

*Dendroiciusqiong* sp. nov. resembles *D.hotaruae* in having similar habitus and copulatory organs, especially the presence of a pair of white lateral setal stripes across the whole surface of carapace, but can be easily distinguished by the absence of latero-terminal tibial apophysis and mesal branch of dorsal tibial apophysis (Fig. 3C) vs present (Lin and Li 2020: fig. 3C) and by the distance between copulatory openings (CO), which is ~ 3/4 of epigynal width, and the C-shaped copulatory ducts (CD) (Fig. 4A–C) vs distance between copulatory openings ~ 1/3 of epigynal width, and nearly S-shaped copulatory ducts (Lin and Li 2020: fig. 4A, B).

##### Description.

**Male** (Figs 3, 4D, E, G, H). Total length 2.91. Carapace 1.40 long, 0.89 wide. Abdomen 1.53 long, 0.80 wide. Eye sizes and interdistances: AME 0.28, ALE 0.14, PLE 0.13, AERW 0.80, PERW 0.84, EFL 0.47. Legs: I 2.19 (0.68, 0.45, 0.53, 0.33, 0.20), II 1.74 (0.53, 0.33, 0.38, 0.30, 0.20), III 1.63 (0.55, 0.25, 0.30, 0.33, 0.20), IV 2.19 (0.70, 0.33, 0.53, 0.40, 0.23). Carapace almost rectangular, yellow-brown except eye field dark, covered with dense dark setae; fovea indistinct. Chelicerae red-brown, with two promarginal teeth and one retromarginal tooth. Legs pale except femora dark brown, with two pairs of spines on tibiae and metatarsi I. Dorsum of abdomen grey, with six longitudinal, dark green and green-brown stripes extending across complete surface; venter pale.

***Palp*** (Fig. 3A–C): femur length/width ratio ca 2.0; patella almost as long as wide in retrolateral view; tibia almost as long as patella in retrolateral view; retrolateral tibial apophysis (RTA) lamellar, with dorsal spinous denticle (DD); dorsal tibial apophysis (DTA) wider than long, with several anteromarginal denticles; cymbium ~ 1.8× longer than wide, with almost horizontal tip and flat baso-retrolateral process (CP); tegulum elongate-oval, swollen at posterior half, with irregular anterior lobe (TL) and small disto-retrolateral bump (TB); embolus (E) strongly sclerotized, tapered, almost as long as anterior tegular lobe, slightly curved medially and pointed apically.

**Female** (Fig. 4A–C, F). Total length 2.66. Carapace 1.12 long, 0.77 wide. Abdomen 1.64 long, 0.80 wide. Eye sizes and interdistances: AME 0.26, ALE 0.13, PLE 0.12, AERW 0.70, PERW 0.74, EFL 0.49. Legs: I 1.49 (0.48, 0.28, 0.30, 0.25, 0.18), II 1.31 (0.40, 0.28, 0.25, 0.20, 0.18), III 1.41 (0.45, 0.20, 0.30, 0.28, 0.18), IV 1.99 (0.63, 0.30, 0.48, 0.35, 0.23). Habitus (Fig. 4F) similar to that of male except paler and with pair of longitudinal, white setal stripes laterally on carapace.

***Epigyne*** (Fig. 4A–C) wider than long, with posterior concave > 3× wider than long; atrium (At) oval, paired, with anterior arc-shaped ridges (AR); copulatory openings (CO) almost round, laterally opened, separated from each other ~ 3/4 epigynal width; copulatory ducts (CD) curved into C-shape, and with small terminal accessory glands (AG); spermathecae (S) elongated.

##### Distribution.

Known only from the type locality in Hainan, China (Fig. 47).

#### ﻿Genus Icius

Taxon classificationAnimaliaAraneaeSalticidae

Simon, 1876

9E896C4C-09A5-5277-9FB7-F8F7A6036CDE

##### Type species.

*Marpissahamata* C. L. Koch, 1846; type locality Naples, Italy.

##### Comments.

*Icius*, one of the most species-rich genera of Chrysillini, comprises 47 species widely distributed in five continents (Maddison 2015; WSC 2024). The genus has not been revised recently, and 20 of its species are known only from a single sex (WSC 2024). The species are rather diverse in habitus and copulatory organs, especially the south and east Asian and African members, indicating that it is likely polyphyletic.

#### ﻿ Icius deergong sp. nov.

Taxon classificationAnimaliaAraneaeSalticidae

371DCA83-937F-5C9F-8BE4-AE81C8971C80

https://zoobank.org/B0961AB8-F6A6-4E8B-B7AA-E18C6341DB1A

Figs 5 , 6 , 47

##### Type material.

***Holotype*** ♂ (TRU-JS 0732), China: • Xizang Autonomous Region, Medog County, Beibeng Township, Deergong Village, Yarlung Zangbo National Nature Reserve (29°10.84'N, 95°8.67'E, ca 1670 m), 25.V.2024, X.Q. Mi et al. leg. ***Paratypes*** • 1 ♂ 3 ♀ (TRU-JS 0733–0736), same data as for holotype.

##### Etymology.

The specific name refers to the type locality, Deergong Village; noun in apposition.

##### Diagnosis.

**Figure 5.**
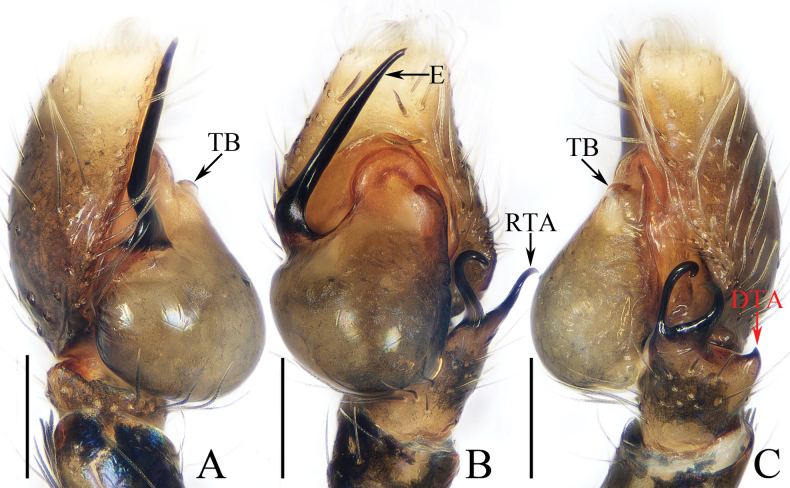
Male palp of *Iciusdeergong* sp. nov., paratype (TRU-JS 0733) **A** prolateral **B** ventral **C** retrolateral. Abbreviations: DTA dorsal tibial apophysis; E embolus; RTA retrolateral tibial apophysis; TB tegular bump. Scale bars: 0.1 mm.

**Figure 6.**
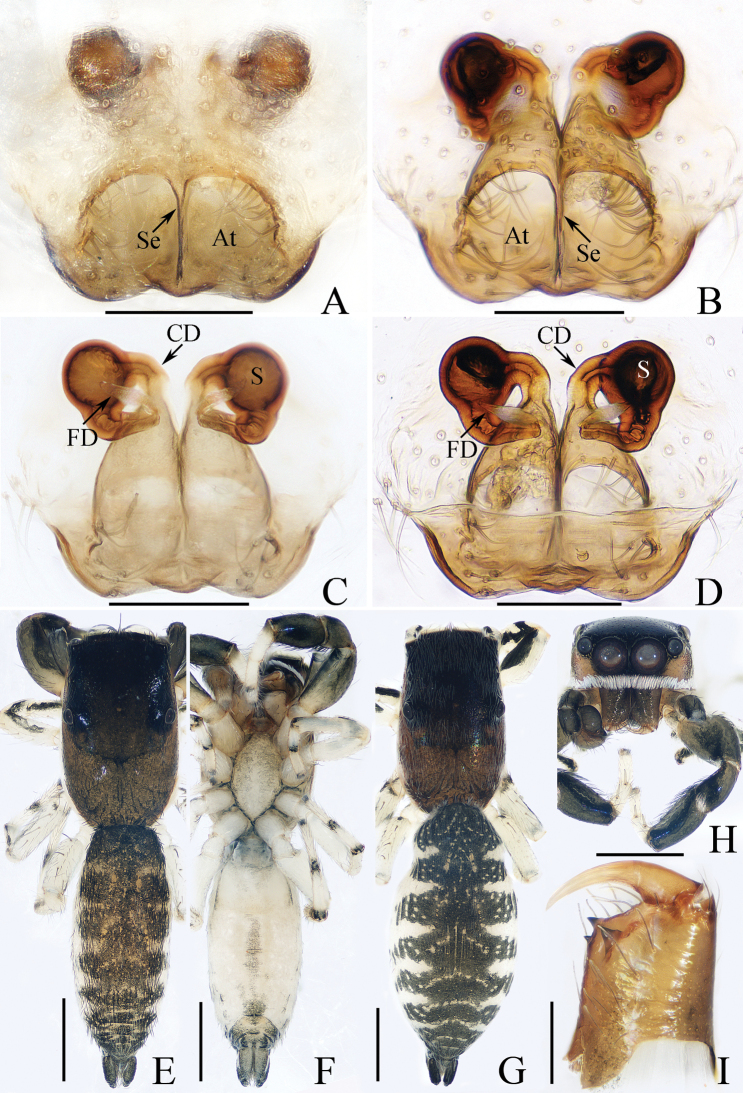
*Iciusdeergong* sp. nov., **E, F, H, I** male holotype and **A–D, G** female paratype (TRU-JS 0734) **A, B** epigyne, ventral **C, D** vulva, dorsal **E, G** habitus, dorsal **F** ditto, ventral **H** carapace and leg I, frontal **I** chelicera, posterior. Abbreviations: At atrium; CD copulatory duct; FD fertilization duct; S spermatheca ; Se septum. Scale bars: 0.1 mm (**A–D, I**); 0.5 mm (**E–H**).

*Iciusdeergong* sp. nov. resembles *I.yadongensis* Hu, 2001 in general shape of copulatory organs, especially the invert infundibuliform base of copulatory duct, but can be easily distinguished by the bifurcated retrolateral tibial apophysis (RTA), the presence of epigynal septum (Se) and proximally touching copulatory ducts (CD) (Figs 5B, C, 6A–D) vs non-bifurcated retrolateral tibial apophysis, lacking septum and copulatory ducts apart from each other proximally (Hu 2001: fig. 8–247-2, 3, 5–7).

##### Description.

**Male** (Figs 5, 6E, F, H, I). Total length 2.56. Carapace 1.16 long, 0.71 wide. Abdomen 1.43 long, 0.60 wide. Eye sizes and interdistances: AME 0.23, ALE 0.11, PLE 0.11, AERW 0.63, PERW 0.69, EFL 0.51. Legs: I 1.77 (0.53, 0.33, 0.40, 0.28, 0.23), II 1.42 (0.43, 0.25, 0.28, 0.23, 0.23), III 1.47 (0.43, 0.23, 0.30, 0.28, 0.23), IV 1.99 (0.60, 0.30, 0.48, 0.38, 0.23). Carapace elongated, grey-brown except eye field dark, with marginal white scale-like setal stripe; fovea indistinct. Chelicerae with two promarginal teeth and one retromarginal tooth. Legs pale with dark stripes except femora, patellae, and tibiae I dark brown; legs I with thickened femora, patellae, and tibiae, covered with cluster of dark ventral setae on patellae and tibiae. Dorsum of abdomen mainly dark brown, covered with dense dark setae, with several transverse, pale yellow lateral stripes; venter pale.

***Palp*** (Fig. 5A–C): femur length/width ratio ca 2.0; patella slightly wider than long in retrolateral view; tibia ~ 2/3 of patellar length in retrolateral view; retrolateral tibial apophysis (RTA) strongly sclerotized, bifurcated basally with two slender, hook-shaped rami pointed apically; dorsal tibial apophysis (DTA) tiny, with pointed tip; cymbium ~ 1.35× longer than wide; tegulum ~ 1.43× longer than wide, with small, antero-retrolateral bump (TB); embolus (E) originating at ca 9:30 o’clock position, curved ventrally at base, and with rather blunt tip.

**Female** (Fig. 6A–D, G). Total length 2.88. Carapace 1.20 long, 0.73 wide. Abdomen 1.78 long, 0.90 wide. Eye sizes and interdistances: AME 0.24, ALE 0.11, PLE 0.11, AERW 0.63, PERW 0.73, EFL 0.55. Legs: I 1.64 (0.50, 0.28, 0.38, 0.25, 0.23), II 1.38 (0.40, 0.25, 0.30, 0.23, 0.20), III 1.49 (0.45, 0.23, 0.30, 0.28, 0.23), IV 2.11 (0.65, 0.35, 0.45, 0.43, 023). Habitus (Fig. 6G) similar to that of male except darker, and without cluster of dark ventral setae on patellae and tibiae I.

***Epigyne*** (Fig. 6A–D) longer than wide; atrium (At) large, occupying most region of posterior 2/5, separated by narrow septum (Se); copulatory openings (CO) almost round, touching each other; copulatory ducts (CD) tapered into invert infundibuliform on proximal half, then acutely narrowed and forming ca 90° curves; spermathecae (S) almost spherical, with posteriorly extending portions.

##### Distribution.

Known only from the type locality in Xizang, China (Fig. 47).

#### ﻿Genus Irura

Taxon classificationAnimaliaAraneaeSalticidae

Peckham & Peckham, 1901

C25CA6C5-C0A5-5A80-AB3A-AC1F11E292AD

##### Type species.

*Irurapulchra* Peckham & Peckham, 1901; type locality Ceylon, now Sri Lanka.

##### Comments.

This genus is assigned to the subtribe Simaethina Simon, 1903, within the Viciriini Simon, 1901 (Maddison 2015), and contains 21 species known from east, south, and southeast Asia (WSC 2024). The genus is rather poorly studied, as the generotype is lacking diagnostic drawings, and nearly 30% of its species are known only from a single sex (WSC 2024). Moreover, based on our recent study, several Chinese species are mismatched (female and male belong to different species) and need further revision. The following two species are placed in the genus due to similar habitus and copulatory organs to most *Irura* species.

#### ﻿ Irura qiuhangi sp. nov.

Taxon classificationAnimaliaAraneaeSalticidae

E0AB8AE6-1A8C-5B11-B605-DB1B81252241

https://zoobank.org/0743067C-2711-42DE-A8BB-61C2B7496F91

Figs 7 , 8 , 48

##### Type material.

***Holotype*** ♀ (TRU-JS 0737), China: • Yunnan Province, Menghai County, Menghai Township, Manliang Village (21°56.36'N, 100°28.37'E, elevation undetailed), 18.III.2024, Hang Qiu leg. ***Paratype*** • 1 ♂ (TRU-JS 0738), same data as for holotype.

##### Etymology.

The specific name is a patronym in honor of the collector; noun (name) in the genitive case.

##### Diagnosis.

**Figure 7.**
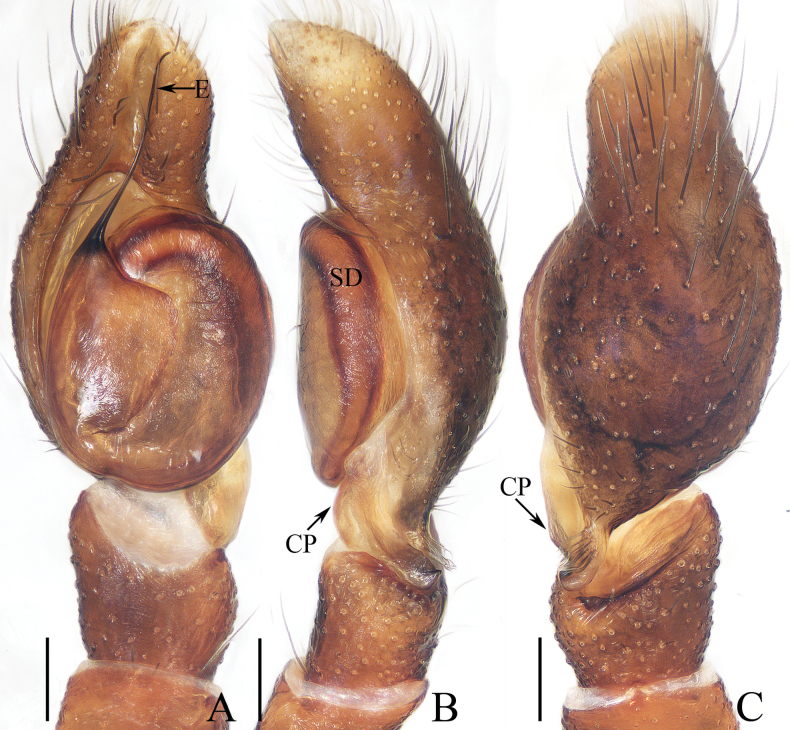
Male palp of *Iruraqiuhangi* sp. nov., paratype (TRU-JS 0738) **A** ventral **B** retrolateral **C** dorsal. Abbreviations: CP cymbial process; E embolus; SD sperm duct. Scale bars: 0.1 mm.

**Figure 8.**
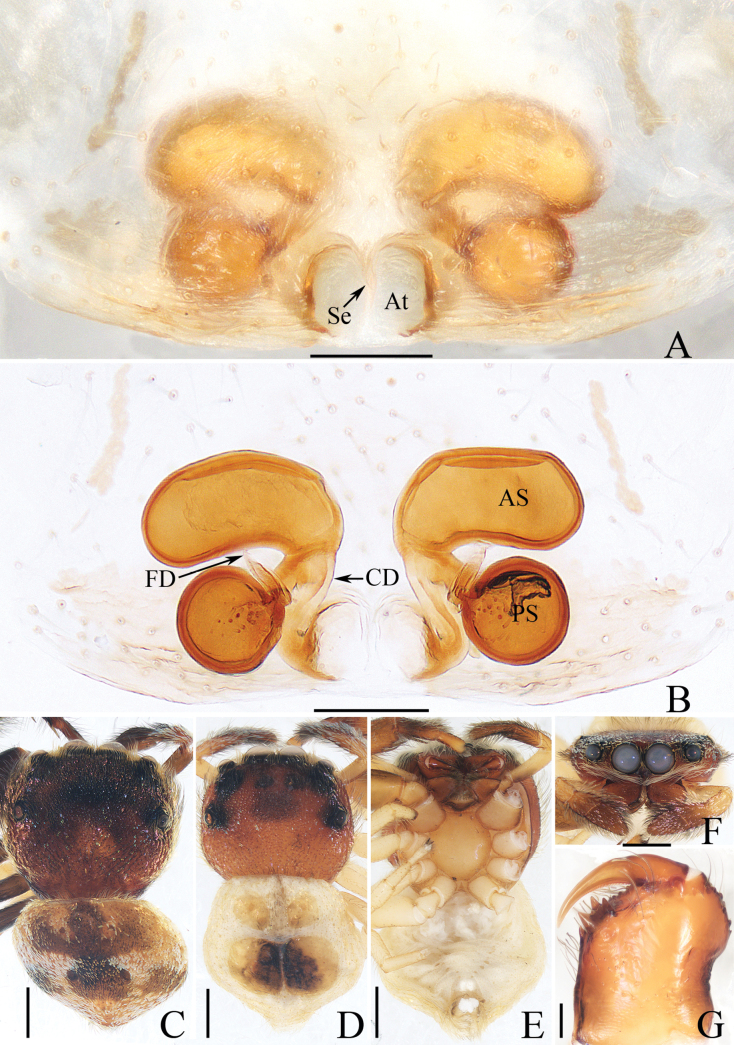
*Iruraqiuhangi* sp. nov., **A**, **B**, **D**–**G** female holotype and **C** male paratype (TRU-JS 0738) **A** epigyne, ventral **B** vulva, dorsal **C**, **D** habitus, dorsal **E** ditto, ventral **F** carapace, frontal **G** chelicera, posterior. Abbreviations: At atrium; AS anterior chamber of spermatheca; CD copulatory duct; FD fertilization duct; PS posterior chamber of spermatheca; Se septum. Scale bars: 0.1 mm (**A, B, G**); 0.5 mm (**C–F**).

The female of *Iruraqiuhangi* sp. nov. resembles that of *I.uniprocessa* Mi & Wang, 2016 in having a similar atrium (At) and transversely extended anterior chamber of spermatheca (AS), but can be easily distinguished by the rounded posterior chamber of spermatheca (PS) (Fig. 8B) vs elongated (Mi and Wang 2016: figs 1G, 2e). The male of *I.qiuhangi* sp. nov. resembles that of *I.shendurney* Asima, Caleb & Prasad, 2024 in the point of origin of the embolus (E) and the form of cymbial process (CP), but can be easily distinguished by the absence of tibial apophysis (Fig. 7B, C) vs retrolateral apophysis present (Asima et al. 2024: figs 41, 44).

##### Description.

**Female** (Fig. 8A, B, D–G). Total length 2.65. Carapace 1.34 long, 1.44 wide. Abdomen 1.38 long, 1.53 wide. Eye sizes and interdistances: AME 0.30, ALE 0.17, PLE 0.16, AERW 1.11, PERW 1.34, EFL 0.49. Legs: I 3.63 (1.05, 0.75, 0.98, 0.50, 0.35), II (0.75, 0.45, 0.45, 0.40, missing), III 2.12 (0.65, 0.38, 0.43, 0.38, 0.28), IV (0.88, 0.43, 0.53, missing, missing). Carapace orange-brown, with pair of round dots behind PMEs, followed by oval, brown patch, covered with pale brown long setae and scales. Chelicerae red-brown, incised on base of anterior surface, with two promarginal teeth and one retromarginal fissidentate tooth with four cusps. Leg I robust, with two pairs of ventral spines on tibiae and metatarsi. Abdomen oval, dorsum pale yellow, with two pairs of large depressions; venter pale, with small pale brown dots medially.

***Epigyne*** (Fig. 8A, B) ~ 1.8× wider than long; atrium (At) almost square, divided by narrow septum (Se); copulatory openings (CO) beneath lateral portions of atrium; copulatory ducts (CD) weakly sclerotized, curved at base, and connected to distal ends of junction ducts of spermathecae (JS); spermathecae (S) divided into transversely extending, kidney-shaped anterior chamber (AS) and round posterior chamber (PS); fertilization ducts (FD) originating from antero-inner portions of posterior chamber of spermatheca.

**Male** (Figs 7, 8C). Total length 2.62. Carapace 1.38 long, 1.58 wide. Abdomen 1.35 long, 1.68 wide. Eye sizes and interdistances: AME 0.31, ALE 0.18, PLE 0.17, AERW 1.19, PERW 1.50, EFL 0.61. Legs: I 4.75 (1.50, 1.15, 1.00, 0.65, 0.45), II 2.68 (0.90, 0.50, 0.50, 0.48, 0.30), III 2.19 (0.75, 0.38, 0.38, 0.40, 0.28), IV 2.70 (0.90, 0.50, 0.50, 0.50, 0.30). Carapace (Fig. 8C) red-brown, with central irregular dark patch on cephalon, covered with dense thin setae and scales. Abdomen (Fig. 8C) oval, dorsum with irregular brown patch, and without similar large shallow depressions as in female; venter brown.

***Palp*** (Fig. 7A–C): femur length/width ratio ca 2.8; patella ~ 1.4× longer than wide; tibia slightly longer than wide, lacking apophyses; cymbium ~ 2× longer than wide, with sizeable baso-retrolateral process (CP) curved medially and with pointed end; tegulum flat, almost round, with sperm duct (SD) extending along submargin; embolus (E) originating at ca 10 o’clock position, ~ 5/6 tegular length, flagelliform.

##### Distribution.

Known only from the type locality in Yunnan, China (Fig. 48).

##### Comments.

As the female can be more easily distinguished from other congeners than the male, it was chosen as the holotype.

#### ﻿ Irura yarlungzangbo sp. nov.

Taxon classificationAnimaliaAraneaeSalticidae

457EB737-C636-5BB4-AF7A-5830D2CF5EC1

https://zoobank.org/34323023-3011-48DA-B5B9-0BF3A5FFA9DC

Figs 9 , 10 , 47

##### Type material.

***Holotype*** ♀ (TRU-JS 0739), China: • Xizang Autonomous Region, Medog County, Beibeng Township, Deergong Village, Yarlung Zangbo National Nature Reserve (29°10.84'N, 95°8.67'E, ca 1670 m), 25.V.2024, X.Q. Mi et al. leg. ***Paratypes*** • 1 ♂ 2 ♀ (TRU-JS 0740–0742), same data as for holotype.

##### Etymology.

The specific name refers to the Yarlung Zangbo National Nature Reserve, the type locality; noun in apposition.

##### Diagnosis.

**Figure 9.**
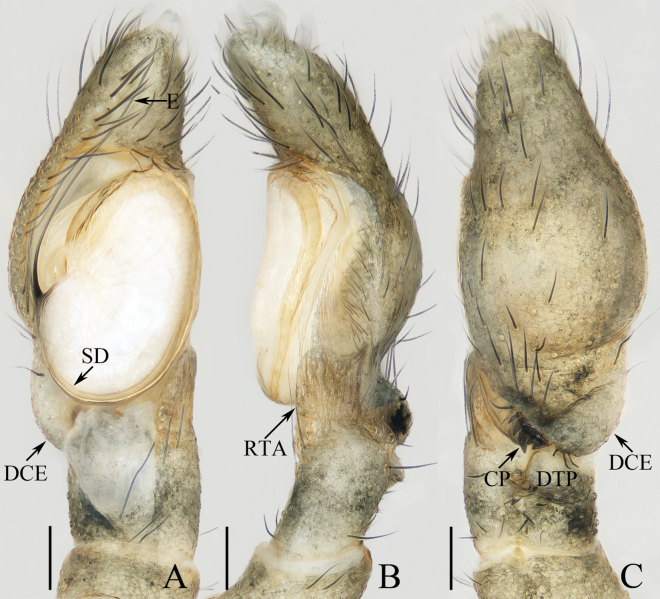
Male palp of *Irurayarlungzangbo* sp. nov., paratype (TRU-JS 0740) **A** ventral **B** retrolateral **C** dorsal. Abbreviations: CP cymbial process; E embolus; DCE dorsal cymbial extension; DTP dorsal tibial process; RTA retrolateral tibial apophysis; SD sperm duct. Scale bars: 0.1 mm.

**Figure 10.**
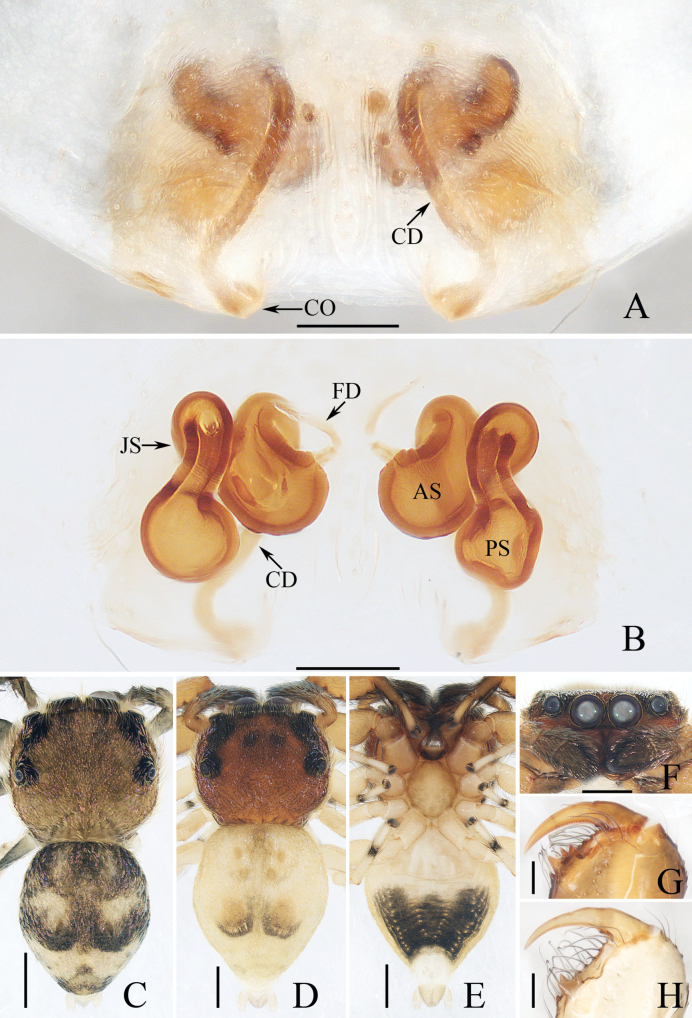
*Irurayarlungzangbo* sp. nov. **A, B, D–G** female holotype and **C, H** male paratype (TRU-JS 0740) **A** epigyne, ventral **B** vulva, dorsal **C, D** habitus, dorsal **E** ditto, ventral **F** carapace, frontal **G, H** chelicera, posterior. Abbreviations: AS anterior chamber of spermatheca; CD copulatory duct; CO copulatory opening; FD fertilization duct; JS junction duct of spermatheca; PS posterior chamber of spermatheca. Scale bars: 0.1 mm (**A, B, G, H**); 0.5 mm (**C–F**).

The female of *Irurayarlungzangbo* sp. nov. resembles that of *I.zhangae* Gan, Wang & Peng, 2017 in having a similar epigyne, but can be easily distinguished by the anterior chamber of spermatheca (AS), ~ 1.3× longer than wide (Fig. 10B) vs ~ 2× longer than wide (Gan et al. 2017: fig. 2F, G, 3D, E), and by the absence of an incision between copulatory openings (Fig. 10A) vs having a square incision between copulatory openings (Gan et al. 2017: figs 2F, G, 3D, E). The male can be easily distinguished by the presence of dorsal cymbial extension (DCE), which bears several retromarginal spines (Fig. 9B, C) vs absent in other congeners (see Metzner 2024).

##### Description.

**Female** (Fig. 10A, B, D–G). Total length 3.78. Carapace 1.43 long, 1.65 wide. Abdomen 2.05 long, 1.65 wide. Eye sizes and interdistances: AME 0.38, ALE 0.22, PLE 0.19, AERW 1.31, PERW 1.55, EFL 0.74. Legs: I 3.28 (1.05, 0.75, 0.65, 0.48, 0.35), II 2.31 (0.75, 0.45, 0.45, 0.38, 0.28), III 2.02 (0.63, 0.35, 0.38, 0.38, 0.28), IV 2.49 (0.85, 0.43, 0.50, 0.43, 0.28). Carapace red-brown, covered with thin brown setae and pale scales, with pair of dark spots centrally on eye field. Chelicerae incised on base of anterior surface, with two promarginal teeth and one retromarginal fissidentate tooth with three cusps. Leg I robust, with two pairs of ventral spines on tibiae and metatarsi. Abdomen oval, dorsum mainly pale, with two pairs of anterior muscle depressions and medio-posterior shallow depressions surrounded by brown C-shaped stripes; venter with dark brown posterior half, and two pairs of pale yellow dotted lines.

***Epigyne*** (Fig. 10A, B) ~ 1.65× wider than long, weakly sclerotized; copulatory openings (CO) postero-marginally located, opened posterolaterally, separated by > 1/3 epigynal width; copulatory ducts (CD) thin, slightly curved proximally and distally, and connected to distal portions of junction ducts of spermathecae (JS); spermathecae (S) divided into oval anterior chamber (AS) and spherical posterior chamber (PS); fertilization ducts (FD) originating at antero-inner margins of anterior chamber of spermatheca.

**Male** (Figs 9, 10C, H). Total length 2.63. Carapace 1.29 long, 1.37 wide. Abdomen 1.41 long, 1.22 wide. Eye sizes and interdistances: AME 0.35, ALE 0.20, PLE 0.15, AERW 1.14, PERW 1.31, EFL 0.65. Legs: I 3.53 (1.05, 0.75, 0.78, 0.50, 0.45), II 2.34 (0.75, 0.43, 0.45, 0.43, 0.28), III 2.00 (0.63, 0.33, 0.38, 0.38, 0.28), IV missing. Carapace (Fig. 10C) brown, covered with purplish gold scales. Chelicerae (Fig. 10H) similar to that of female except retromarginal fissidentate tooth only with two cusps. Legs brown, mingled with green. Abdomen (Fig. 10C) oval, dorsum mainly dark brown, covered with purplish gold scales, with pair of pale median patches and transverse, posterior, pale band; venter dark brown.

***Palp*** (Fig. 9A–C): femur length/ width ratio ca 2.72; patella ~ 1.6× longer than wide in retrolateral view; tibia slightly longer than patella, with well-developed, lamellar retrolateral apophysis (RTA) and swollen dorsal process (DTP); cymbium ~ 2× longer than wide, with weakly sclerotized retrolateral process (CP) partly covered by retrolateral tibial apophysis and with pointed end, and well developed, posteriorly extending dorsal extension (DCE) bearing several retromarginal spines; tegulum flat, oval; embolus (E) filiform, 1.2× longer than tegulum, originating at ca 9 o’clock position.

##### Distribution.

Known only from the type locality in Xizang, China (Fig. 47).

#### ﻿Genus Mintonia

Taxon classificationAnimaliaAraneaeSalticidae

Wanless, 1984

A69A7F71-9015-50D9-8B1F-358484856680

##### Type species.

*Mintoniatauricornis* Wanless, 1984; type locality Sarawak, Indonesia.

##### Comments.

This genus is placed in the subtribe Spartaeina Wanless, 1984 within the Spartaeini Wanless, 1984 (Maddison 2015). To date, ten species have been described, and all are restricted to Southeast Asia (WSC 2024). A significant taxonomic study of the genus was done by Wanless (1984, 1987), who described eight new species and first illustrated the transferred species, *Mintoniaramipalpis* (Thorell, 1890). However, seven species described by him are only known from a single sex.

#### ﻿ Mintonia shiwandashan sp. nov.

Taxon classificationAnimaliaAraneaeSalticidae

50F4192F-4850-561A-B16E-6C85C4008CCD

https://zoobank.org/6CB986A6-0998-4233-898A-3AA4652F30F8

Figs 11 , 47

##### Type material.

***Holotype*** ♂ (TRU-JS 0743), China: • Guangxi Zhuang Autonomous Region, Fangchenggang City, Shiwandashan National Nature Reverse, Wanglue Station (21°54.23'N, 107°54.18'E, ca 310 m), 30.IV.2021, A.L. He et al. leg.

##### Etymology.

The specific name refers to the type locality: Shiwandashan National Nature Reverse; noun in apposition.

##### Diagnosis.

**Figure 11.**
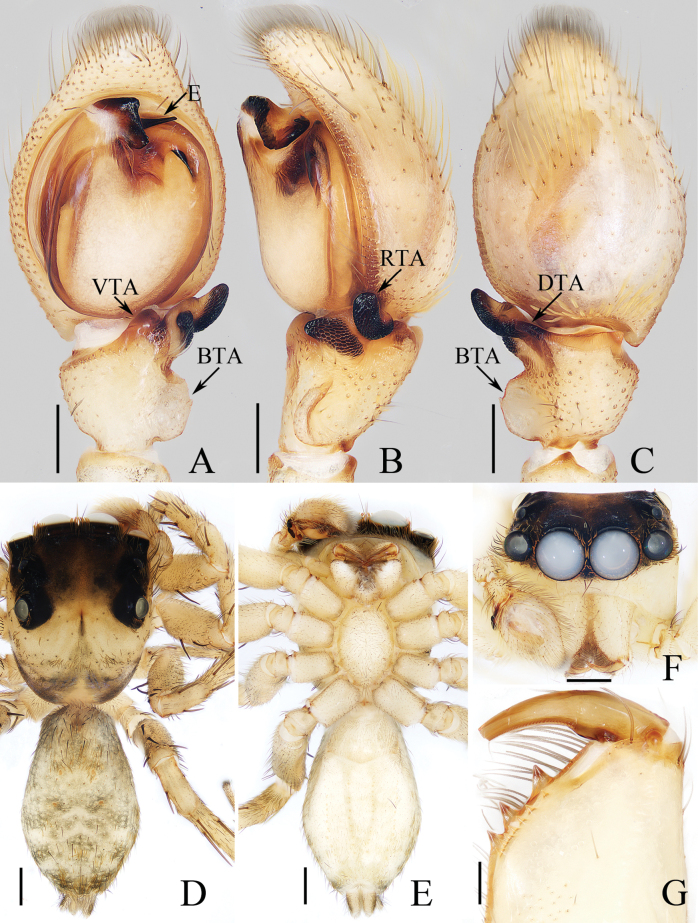
*Mintoniashiwandashan* sp. nov., holotype **A** palp, ventral **B** ditto, retrolateral **C** ditto, dorsal **D** habitus, dorsal **E** ditto, ventral **F** carapace, frontal **G** chelicera, posterior. Abbreviations: BTA baso-retrolateral tibial apophysis; DTA dorsal tibial apophysis; E embolus; RTA retrolateral tibial apophysis; VTA ventral tibial apophysis. Scale bars: 0.2 mm (**A–C, G**); 0.5 mm (**D–F**).

*Mintoniashiwandashan* sp. nov. resembles *M.breviramis* Wanless, 1984 in having very short embolus (E), but can be easily distinguished by the presence of baso-retrolateral and dorsal tibial apophyses, and by the bifurcated retrolateral tibial apophysis (RTA) (Fig. 11B, C) vs lacking baso-retrolateral and dorsal tibial apophyses and having non-bifurcated retrolateral tibial apophysis (Wanless 1984: fig. 12A, B).

**Figure 12.**
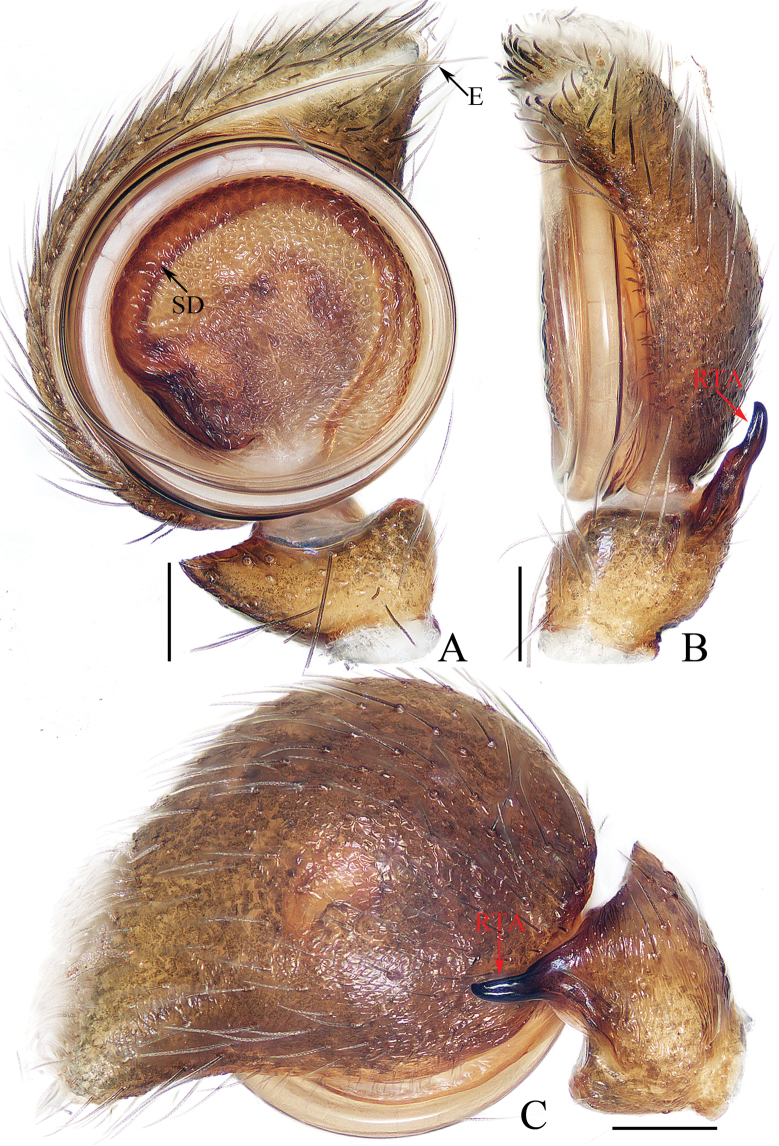
Male palp of *Myrmarachnekuan* sp. nov., paratype (TRU-JS 0745) **A** ventral **B** retrolateral **C** dorsal. Abbreviations: E embolus; RTA retrolateral tibial apophysis; SD sperm duct. Scale bars: 0.1 mm.

##### Description.

**Male** (Fig. 11). Total length 5.10. Carapace 2.43 long, 2.05 wide. Abdomen 2.62 long, 1.71 wide. Eye sizes and interdistances: AME 0.62, ALE 0.38, PLE 0.34, AERW 2.00, PERW 1.86, EFL 1.19. Legs: I 5.92 (1.70, 0.88, 1.53, 1.18, 0.63), II 5.43 (1.60, 0.85, 1.30, 1.10, 0.58), III 5.08 (1.50, 0.50, 1.25, 1.25, 0.58), IV 6.86 (1.95, 0.78, 1.68, 1.75, 0.70). Carapace pale yellow except eye field dark, with elevated and square cephalon, covered with brown and golden thin setae; fovea dark. Chelicerae yellow, with three promarginal and seven smaller retromarginal teeth. Legs yellow, tinged with brown, spiny. Dorsum of abdomen pale to brown, covered with golden and dark setae, with two well-visible pairs of anteromedian muscle depressions; venter pale, with two pairs of dotted lines medially.

***Palp*** (Fig. 11A–C): femur length/width ratio ca 3.3; patella ~ 1.5× longer than wide in retrolateral view; tibia slightly longer than wide in ventral view, with almost half-round, lamellar base-retrolateral apophysis (BTA); ventral tibial apophysis (VTA) almost sub-triangular; retrolateral tibial apophysis (RTA) strongly sclerotized, bifurcated with two blunt rami; dorsal tibial apophysis (DTA) bar-shaped, with blunt end in dorsal view; cymbium ~ 1.47× longer than wide in ventral view; tegulum oval; embolus (E) strongly sclerotized, broad, with tapered projection.

**Female.** Unknown.

##### Distribution.

Known only from the type locality in Guangxi, China (Fig. 47).

##### Comments.

Although the new species is similar to *Portiajianfeng* Song & Zhu, 1998 in palpal structure, it has not been considered to be a member of *Portia* Karsch, 1878 because it lacks tufts on the abdomen and a pronounced dorso-basal flange on the cymbium, which are diagnostic for *Portia* (Wanless 1984). The new species is provisionally placed in the genus *Mintonia* due to the general similarity of palpal structure to current congeners.

#### ﻿Genus Myrmarachne

Taxon classificationAnimaliaAraneaeSalticidae

MacLeay, 1839

299438ED-5D21-5BBE-8BC6-E4F95C289971

##### Type species.

*Myrmarachnemelanocephala* MacLeay, 1839; type locality India.

##### Comments.

*Myrmarachne*, the species-richest genus of the subtribe Myrmarachnina Simon, 1901 within the tribe Myrmarachnini Simon, 1901 (Maddison and Szűts 2019), contains 192 nominal species widely distributed all over the globe (WSC 2024). *Myrmarachne* is one of the most poorly studied genera among the Salticidae since ~ 48.95% of its species are known only from a single sex or sub-adult specimen, > 50 species have not been illustrated or lack essential diagnostic drawings. Moreover, many of its species share similar copulatory organs and present several color patterns, making them difficult to identify. There is no doubt that a few of its species could be potential synonyms or the “missing” sex of another congener. In addition, Prószyński (2016) split and resurrected eleven genera from *Myrmarachne* according to morphological characters. However, this conclusion has not been supported by the subsequent molecular evidence (Yamasaki et al. 2018; Maddison and Szűts 2019). Thus, the phylogenetic relationship between *Myrmarachne* and the mentioned eleven genera remains uncertain.

#### ﻿ Myrmarachne kuan sp. nov.

Taxon classificationAnimaliaAraneaeSalticidae

62A4E42A-CB09-56F6-9425-56B84E0C22AF

https://zoobank.org/99B67F00-8D9E-4D75-AF2D-A5B0E7D327B4

Figs 12 , 13 , 48

##### Type material.

***Holotype*** ♂ (TRU-JS 0744), China: • Yunnan Province, Pingbian Miao Autonomous County, around Tuanpo Reservoir (22°58.33'N, 103°41.25'E, ca 1560 m), 15.V.2024, C. Wang et al. leg. ***Paratypes*** • 2 ♂ 5 ♀ (TRU-JS 0745–0751), same data as for holotype; • 1 ♀ (IZCAS-Ar 45282), Hainan, Lingshui County, Diaoluoshan National Nature Reserve, Power Station (18°39.84'N, 109°55.81'E, ca 100 m), 20.IV.2009, G. Tang leg.

##### Etymology.

The specific name is a noun and comes from Chinese Pinyin ‘kuan’, meaning broad, which refers to the broadened thoracic part.

##### Diagnosis.

**Figure 13.**
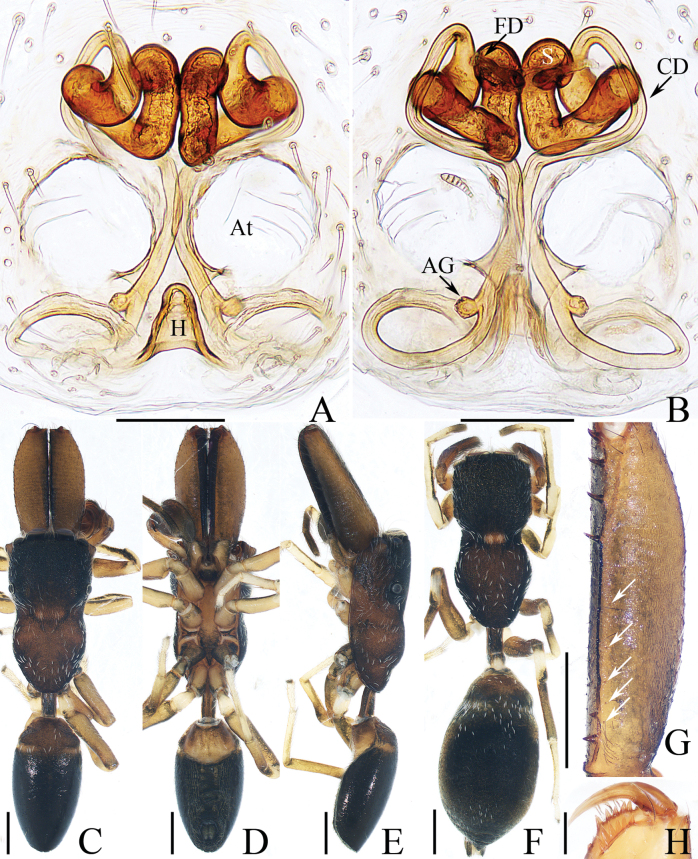
*Myrmarachnekuan* sp. nov. **C–E, G** holotype and **A, B, F, H** female paratype (TRU-JS 0747) **A** epigyne, ventral **B** vulva, dorsal **C, F** habitus, dorsal **D** ditto, ventral **E** ditto, lateral **G, H** chelicera, posterior. Abbreviations: AG accessory gland; At atrium; CD copulatory duct; FD fertilization duct; H epigynal hood; S spermatheca. Scale bars: 0.1 mm (**A, B, H**); 0.5 mm (**C–G**).

The male of *Myrmarachnekuan* sp. nov. resembles that of *M.salaputium* Yamasaki, 2018 in general shape of the palp, but can be easily distinguished by the flat cephalon that is lower than thoracic part in lateral view (Fig. 13E) vs elevated cephalon that is much higher than thoracic part (Yamasaki et al. 2018: fig. 45). The female resembles those of *M.lambirensis* Yamasaki & Ahmad, 2013 in having a similar epigyne, but can be easily distinguished by the presence of an epigynal hood (H), and by the sclerotized portions of copulatory ducts curved into circles at base (Fig. 13A, B) vs epigynal hood absent, and sclerotized portions of copulatory ducts slightly curved into C-shapes (Yamasaki and Ahmad 2013: fig. 23D–F).

**Figure 14.**
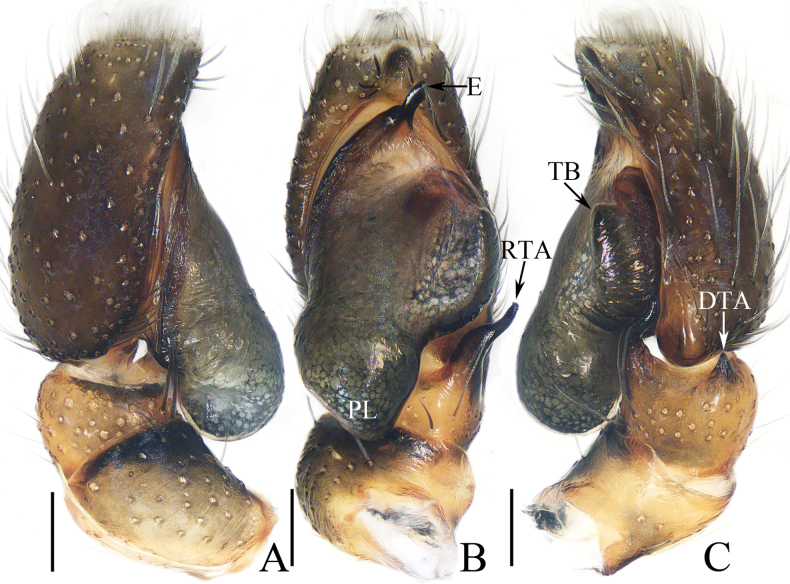
Male palp of *Nandiciusxiefengi* sp. nov., holotype **A** prolateral **B** ventral **C** retrolateral. Abbreviations: DTA dorsal tibial apophysis; E embolus; PL posterior tegular lobe; RTA retrolateral tibial apophysis; TB tegular bump. Scale bars: 0.1 mm.

##### Description.

**Male** (Figs 12, 13C–E, G). Total length 3.72. Carapace 1.80 long, 0.98 wide. Abdomen 1.61 long, 0.83 wide. Eye sizes and interdistances: AME 0.30, ALE 0.15, PLE 0.14, AERW 0.89, PERW 0.98, EFL 0.65. Legs: I 2.91 (0.83, 0.40, 0.88, 0.50, 0.30), II 2.32 (0.65, 0.38, 0.58, 0.43, 0.28), III 2.39 (0.70, 0.33, 0.53, 0.53, 0.30), IV 3.19 (0.95, 0.43, 0.80, 0.68, 0.33). Carapace flat, covered with sparse white scales, with laterally broadened thoracic part. Chelicerae elongated, with six promarginal and five tiny retromarginal teeth. Legs slender, with one, eight, and four ventral spines on patellae, tibiae, and metatarsi I, respectively. Abdomen slightly constricted at anterior 1/5, dorsum mainly dark, covered with several white scales; venter dark brown.

***Palp*** (Fig. 12A–C): femur length/width ratio ca 2.5; patella ~ 1.4× longer than wide in retrolateral view; tibia broad, with prolateral projected portion, and tapered retrolateral apophysis (RTA) approximately as long as tibia, slightly curved inward distally, and with rather pointed tip; cymbium length/width ratio ca 1.37, tapered at distal 1/4; tegulum flat and round, with sperm duct extending along submargin circularly; embolus (E) originating at ca 6 o’clock position of tegulum, making ca 540° course and terminating at ca 1:30 o’clock position.

**Female** (Fig. 13A, B, F, H). Total length 4.33. Carapace 1.88 long, 0.86 wide. Abdomen 1.96 long, 1.12 wide. Eye sizes and interdistances: AME 0.30, ALE 0.15, PLE 0.14, AERW 0.87, PERW 0.92, EFL 0.61. Legs: I 2.44 (0.68, 0.40, 0.68, 0.40, 0.28), II 2.02 (0.58, 0.38, 0.50, 0.33, 0.23), III 2.12 (0.60, 0.33, 0.48, 0.48, 0.23), IV 3.03 (0.90, 0.45, 0.75, 0.63, 0.30). Habitus (Fig. 13F) similar to that of male except less-developed chelicerae (Fig. 13H) with six larger retromarginal teeth.

***Epigyne*** (Fig. 13A, B) longer than wide, with posterior, bell-shaped hood (H); atrium (At) paired, almost round; copulatory openings (CO) invisible; sclerotized portions of copulatory ducts slender, forming complicated coils, and with short accessory glands (AG) on position of proximal 2/5; spermathecae (S) elongated, folded twice; fertilization ducts (FD) originating from antero-inner portions of spermathecae.

##### Distribution.

China (Hainan, Yunnan; Fig. 48).

#### ﻿Genus Nandicius

Taxon classificationAnimaliaAraneaeSalticidae

Prószyński, 2016

F126F149-1356-50FF-A0B8-155CDF94897F

##### Type species.

*Phintellamussooriensis* Prószyński, 1992; type locality Mussoorie, India.

##### Comments.

This genus was recently considered to be a member of Chrysillini (Yang & Zhang, 2024). To date, 13 species are known from Afghanistan to Japan (WSC 2024). Within the genus, many species (> 46%) are known only from a single sex (WSC 2024), and several members most likely are misplaced and need to be further revised, such as *N.proszynskii* Wang & Li, 2021 (Yunnan, China), *N.shihaitaoi* Wang & Li, 2023 (Hainan, China), and *N.woongilensis* Kim & Lee, 2016 (Korea). The first two could be related to *Iciusindicus* (Simon, 1901), and the last one may belong to *Pseudeuophrys* Dahl, 1912.

#### ﻿ Nandicius xiefengi sp. nov.

Taxon classificationAnimaliaAraneaeSalticidae

503DAD8A-4E1E-5D43-BB9E-AFA379550209

https://zoobank.org/277A2491-14F1-4E0A-871C-9D4BF0506035

Figs 14 , 15 , 48

##### Type material.

***Holotype*** ♂ (TRU-JS 0752), China: • Xizang Autonomous Region, Medog County, Damu Township, Zhu Village (29°29.73'N, 95°25.86'E, ca 1740 m), 27.V.2024, X.F. Wang. leg. ***Paratype*** 1 ♀ (TRU-JS 0753), same data as for holotype.

##### Etymology.

The specific name is a patronym in honor of the collector; noun (name) in the genitive case.

##### Diagnosis.

**Figure 15.**
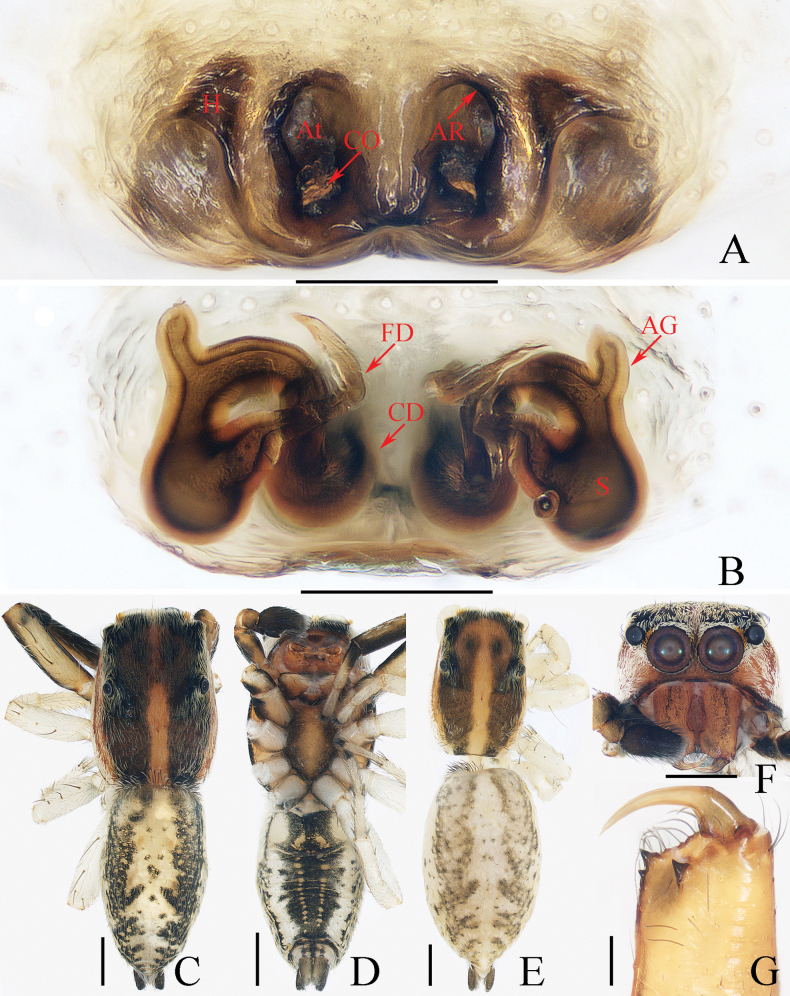
*Nandiciusxiefengi* sp. nov. **C, D, F, G** male holotype and **A, B, E** female paratype (TRU-JS 0753) **A** epigyne, ventral **B** vulva, dorsal **C**, **E** habitus, dorsal **D** ditto, ventral **F** carapace, frontal **G** chelicera, posterior. Abbreviations: AG accessory gland; AR atrial ridge; At atrium; CD copulatory duct; CO copulatory opening; FD fertilization duct; H epigynal hood; S spermatheca. Scale bars: 0.1 mm (**A, B, G**); 0.5 mm (**C–F**).

The male of *Nandiciusxiefengi* sp. nov. resembles that of *N.gyirongensis* (Hu, 2001) in having similar retrolateral tibial apophysis (RTA), but can be easily distinguished by the following: 1) embolus (E) curved towards antero-retrolateral side (Fig. 14B) vs antero-prolateral side (Yang and Zhang 2024: figs 155, 161); 2) posterior tegular lobe (PL) extending posteriorly (Fig. 14B) vs extending prolatero-posteriorly (Yang and Zhang 2024: figs 155, 161). The female of *N.xiefengi* sp. nov. can be easily distinguished from congeners by the anteriorly located epigynal hood (H) (Fig. 15A) vs posteriorly located in the others (see Metzner 2024).

##### Description.

**Male** (Figs 14, 15C, D, F, G). Total length 3.68. Carapace 1.84 long, 1.32 wide. Abdomen 1.95 long, 1.08 wide. Eye sizes and interdistances: AME 0.34, ALE 0.16, PLE 0.15, AERW 1.08, PERW 1.11, EFL 0.81. Legs: I 3.88 (1.05, 0.65, 0.90, 0.90, 0.38), II 2.64 (0.78, 0.48, 0.60, 0.45, 0.33), III 2.74 (0.93, 0.43, 0.50, 0.53, 0.35), IV 3.12 (0.93, 0.43, 0.70, 0.68, 0.38). Carapace mainly dark brown, with pair of elongated, dark patches centrally on cephalon, and longitudinal, orange band extending from middle between PMEs to posterior end, covered with dense pale and dark setae. Chelicerae orange, with two promarginal teeth and one retromarginal tooth. Legs pale except legs I mottled with dark. Dorsum of abdomen mainly green-brown, with longitudinal, irregular pale patch extended over whole surface, and two pairs of median muscle depressions; venter pale laterally, and dark brown centrally, with pair of longitudinal, central dotted lines.

***Palp*** (Fig. 14A–C): femur length/width ratio ca 3.2; patella almost as long as wide in retrolateral view; tibia ~1.3× wider than long in retrolateral view; retrolateral tibial apophysis (RTA) strongly sclerotized, curved medially, and with rather blunt tip; dorsal tibial apophysis (DTA) sub-triangular; cymbium ~ 1.6× longer than wide, with hollow against embolus; tegulum longer than cymbium, swollen medio-posteriorly, with posteriorly extended posterior lobe (PL), and disto-retrolateral bump (TB); embolus (E) strongly sclerotized, short, slightly curved, with rather blunt end.

**Female** (Fig. 15A, B, E). Total length 4.24. Carapace 1.68 long, 1.14 wide. Abdomen 2.41 long, 1.46 wide. Eye sizes and interdistances: AME 0.32, ALE 0.16, PLE 0.16, AERW 0.98, PERW 1.06, EFL 0.71. Legs: I 2.56 (0.75, 0.50, 0.63, 0.38, 0.30), II 2.31 (0.70, 0.45, 0.53, 0.35, 0.28), III 2.56 (0.78, 0.43, 0.50, 0.55, 0.30), IV 3.29 (1.03, 0.48, 0.80, 0.68, 0.30). Habitus (Fig. 15E) similar to that of male but paler.

***Epigyne*** (Fig. 15A, B) > 2× wider than long, with pair of anterior hoods (H) lateral to atrium (At); atrium almost square, with pair of lateral auricle-shaped ridges (AR); copulatory openings (CO) posteriorly located on atrium, irregular; copulatory ducts (CD) strongly curved circularly at proximal, then curved to C-shape, with bar-shaped, terminal accessory glands (AG); spermathecae (S) sub-spherical, with antero-inner extensions.

##### Distribution.

Known only from the type locality in Xizang, China (Fig. 48).

#### ﻿Genus Okinawicius

Taxon classificationAnimaliaAraneaeSalticidae

Prószyński, 2016

5AD30E32-4821-5289-9C5B-1130C04062CE

Okinawicius Prószyński, 2016: 22.
Nepalicius Prószyński, 2016: 21. Syn. nov.

##### Type species.

*Pseudiciusokinawaensis* Prószyński, 1992; type locality Okinawa.

##### Diagnosis and description.

See Prószyński (2016).

##### Composition.

This genus currently includes 12 species: *Okinawiciusdaitaricus* (Prószyński, 1992) (♀); *O.daoxianensis* (Peng, Gong & Kim, 2000), comb. nov. (♂); *O.delesserti* (Caporiacco, 1941) (♂); *O.modestus* (Simon, 1885) (♀); *O.nepalicus* (Andreeva, Hęciak & Prószyński, 1984), comb. nov. (♂♀); *O.okinawaensis* (Prószyński, 1992) (♀); *O.seychellensis* (Wanless, 1984), comb. nov. (♂♀); *O.sheherezadae* (Prószyński, 1989) (♀); *O.shirinae* (Prószyński, 1989) (♂); *O.sindbadi* (Prószyński, 1989) (♂); *O.tekdi* Tripathi & Kulkarni, 2024 (♂♀); *O.tokaraensis* (Bohdanowicz & Prószyński, 1987) (♂♀).

##### Comments.

Both *Nepalicius* and *Okinawicius* were described by Prószyński (2016). They are considered to be congeneric because the newly discovered females of *N.nepalicus* (the generotype of *Nepalicius*) share consistent habitus and epigyne with *O.okinawaensis* (the generotype of *Okinawicius*, known only from females), especially the copulatory ducts that form several coils in a plane almost perpendicular to the vertical axis. Thus, *Nepalicius* is proposed as a synonym of *Okinawicius*. We act as First Revisor per ICZN (1999). *Okinawiciusdaoxianensis* (Peng, Gong & Kim, 2000), comb. nov. is transferred due to it having the round tegulum encircled by embolus and with dorsal ramus of retrolateral tibial apophysis reduced to a triangular protuberance, which is consistent with *O.nepalicus*. As Yang et al. (2024) mentioned, some *Afraflacilla* species, only known by males, may also belong to *Okinawicius*. Moreover, the relationship between *Okinawicius* and *Afraflacilla* also needs further attention.

#### ﻿ Okinawicius nepalicus

Taxon classificationAnimaliaAraneaeSalticidae

(Andreeva, Hęciak & Prószyński, 1984) comb. nov.

6293DBB2-A34C-5F09-84DE-0721CAF05FF5

Figs 16 , 17 , 47

Icius nepalicus Andreeva, Hęciak & Prószyński, 1984: 372, figs 49–51 (holotype ♂, not examined).
Pseudicius nepalicus : Prószyński, 1992: 106, figs 67, 69–72 (♂).
Nepalicius nepalicus : Prószyński, 2016: 22, fig. 7A, B (transferred from Pseudicius).

##### Note.

For a complete reference list of the species, see WSC (2024).

##### Material examined.

2 ♂ 3 ♀ (TRU-JS 0754–0758), China: • Xizang Autonomous Region, Medog County, Beibeng Township, Deergong Village, Yarlung Zangbo National Nature Reserve (29°10.84'N, 95°8.67'E, ca 1670 m), 25.V.2024, X.Q. Mi et al. leg.

##### Diagnosis.

**Figure 16.**
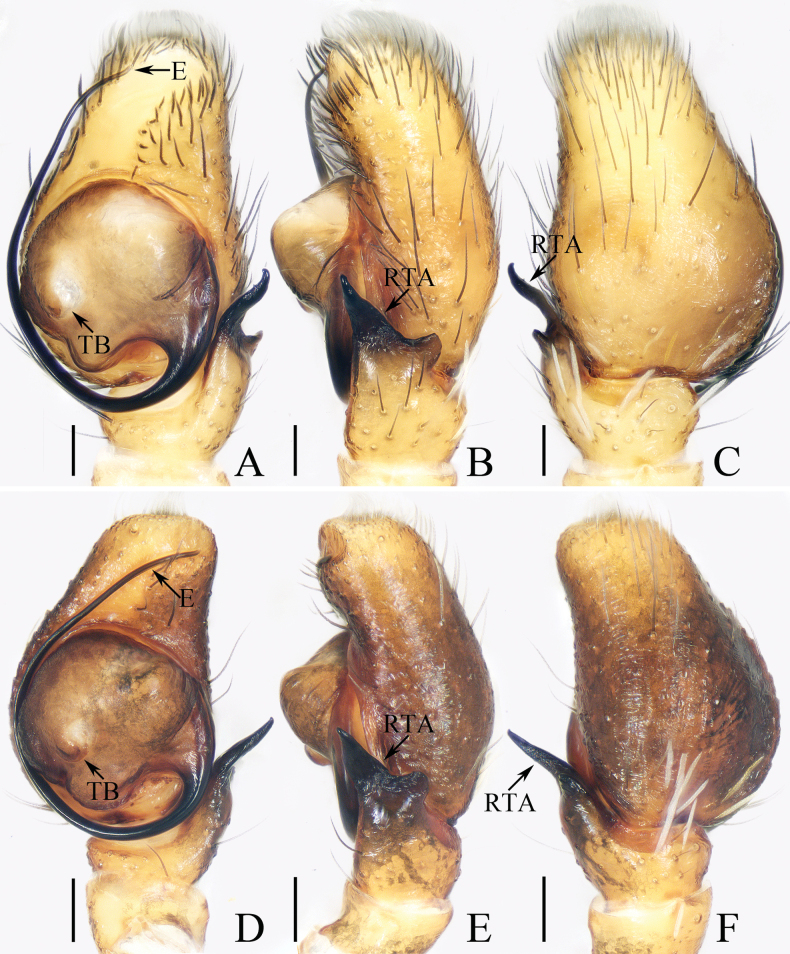
Male palp of *Okinawiciusnepalicus* (Andreeva, Hęciak & Prószyński, 1984) **A–C**TRU-JS 0754 **D–F**TRU-JS 0755 **A, D** ventral **B, E** retrolateral **C, F** dorsal. Abbreviations: E embolus; RTA retrolateral tibial apophysis; TB tegular bump. Scale bars: 0.1 mm.

**Figure 17.**
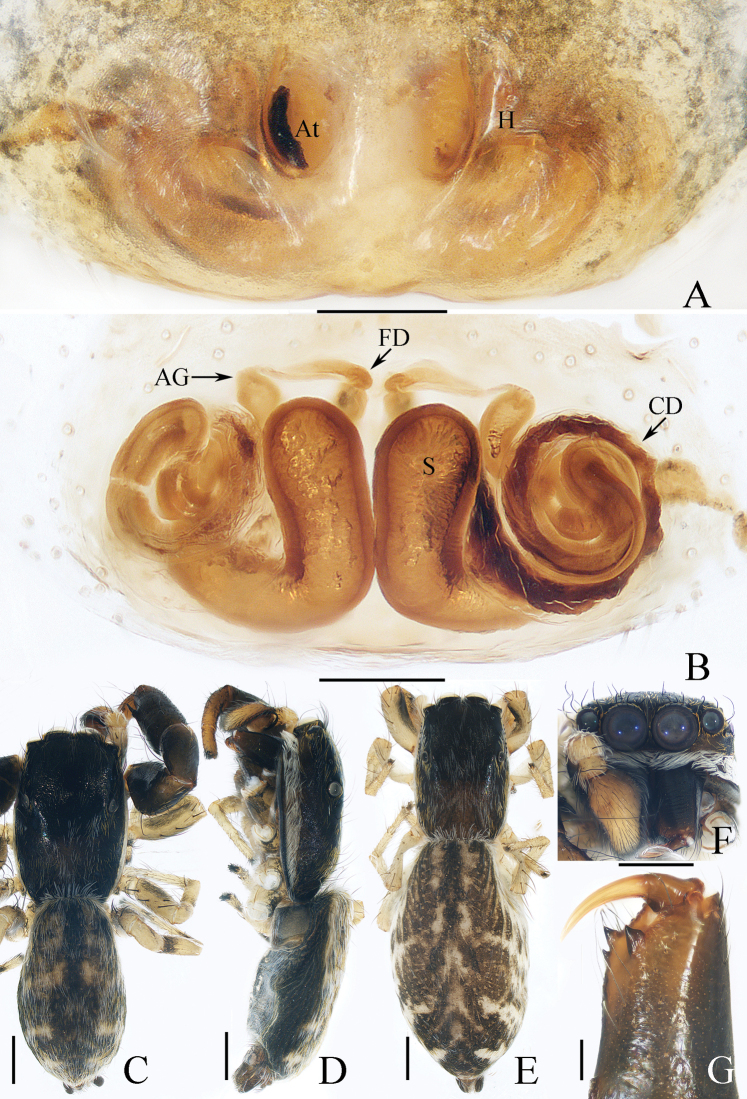
*Okinawiciusnepalicus* (Andreeva, Hęciak & Prószyński, 1984) **C, D, F, G** male (TRU-JS 0754) and **A, B, E** female (TRU-JS 0756) **A** epigyne, ventral **B** vulva, dorsal **C, E** habitus, dorsal **D** ditto, lateral **F** carapace, frontal **G** chelicera, posterior. Abbreviations: AG accessory gland; At atrium; CD copulatory duct; FD fertilization duct; H epigynal hood; S spermatheca. Scale bars: 0.1 mm (**A, B, G**); 0.5 mm (**C–F**).

This species resembles *O.tokaraensis* in having very similar habitus and copulatory organs, especially the epigynal structure, but differs in: 1) embolus (E) originating at ca 4 o’clock position (Fig. 16A, D) vs ca 6:30 – ca 9:00 o’clock position (Yang et al. 2024: figs 80–83); 2) membranous portions of copulatory ducts make 2 coils (Fig. 17B) vs ~ 3 coils (Yang et al. 2024: figs 89, 91, 93, 99–101).

##### Re-description.

**Male** (Figs 16, 17C, D, F, G). Total length 3.69. Carapace 1.79 long, 1.25 wide. Abdomen 2.01 long, 1.17 wide. Eye sizes and interdistances: AME 0.32, ALE 0.19, PLE 0.18, AERW 1.01, PERW 1.02, EFL 0.77. Legs: I 3.79 (1.13, 0.75, 1.00, 0.63, 0.28), II 2.53 (0.80, 0.48, 0.55, 0.40, 0.30), III 2.65 (0.80, 0.40, 0.55, 0.60, 0.30), IV 3.34 (1.00, 0.50, 0.83, 0.73, 0.28). Carapace dark, with marginal white setal band, covered with dense pale, golden and dark setae. Chelicerae mainly dark, with two promarginal teeth and one retromarginal tooth. legs I with thickness femora, patellae and tibiae, and single pro-ventral spine on tibiae. Dorsum of abdomen dark brown, with four pairs of transverse, pale stripes laterally; venter colored as dorsum.

***Palp*** (Fig. 16A–F): femur length/width ratio ca 2.44; patella almost as long as wide in retrolateral view; tibia ~ 1.6× wider than long in ventral view, with strongly sclerotized retrolateral apophysis (RTA) bifurcated into sub-triangular ventral ramus and sub-semicircular dorsal ramus; cymbium ~ 1.54× longer than wide; tegulum almost round, with prolatero-posterior bump (TB) and antero-retrolateral swollen portion; embolus (E) originating at ca 4 o’clock position, coiled in less than complete circle, with rather blunt tip.

**Female** (Fig. 17A, B, E). Total length 4.40. Carapace 1.72 long, 1.14 wide. Abdomen 2.86 long, 1.64 wide. Eye sizes and interdistances: AME 0.34, ALE 0.17, PLE 0.15, AERW 0.91, PERW 1.01, EFL 0.71. Legs: I 2.79 (0.88, 0.63, 0.63, 0.40, 0.25), II 2.28 (0.65, 0.5, 0.5, 0.38, 0.25), III 2.55 (0.75, 0.45, 0.50, 0.55, 0.30), IV 3.52 (1.08, 0.58, 0.83, 0.73, 0.30). Habitus (Fig. 17E) similar to that in male except paler and with thinner femora, patellae, and tibiae I.

***Epigyne*** (Fig. 17A, B) ~ 2× wider than long, with pair of anteriorly located hoods (H) ~ 1.5× longer than wide; atrium (At) almost oval; copulatory openings (CO) indistinct; copulatory ducts (CD) forming ~ 2 coils, with terminal bar-shaped accessory glands (AG) curved medially; spermathecae (S) tube-shaped, touching each other.

##### Distribution.

China (Xizang; Fig. 47), India (Tamil Nadu), Nepal (Kathmandu).

##### Comments.

Although the male specimens described here are almost identical to the holotype, they also have some differences, such as the origin of embolus, which arises at ca 4 o’clock position (vs ca 3 o’clock in the holotype; see Andreeva et al. 1984: fig. 49), those are here considered as interspecific variations.

#### ﻿Genus Padillothorax

Taxon classificationAnimaliaAraneaeSalticidae

Simon, 1901

02A73916-8DF9-52CD-BDEB-CC064D39A214

##### Type species.

*Padillothoraxsemiostrinus* Simon, 1901; type locality Malaysia.

##### Comments.

This genus is considered to be a member of Baviini Simon, 1901 (Maddison 2015; Maddison et al. 2020). It has always been poorly known, from only two nominal species until a proper redefinition was provided by Maddison et al. (2020), who first illustrated the generotype, proposed three new combinations and added two new members. Further taxonomic attention to the genus is also essential because three members, including the generotype, remain known only from a single sex and *P.taprobanicus* Simon, 1902 lacks diagnostic drawings (WSC 2024).

#### ﻿ Padillothorax exilis

Taxon classificationAnimaliaAraneaeSalticidae

(Cao & Li, 2016)

1B576DFF-2C50-5BF3-B167-15DAC41F2D84

Figs 18 , 19 , 48

Bavia exilis Cao & Li, in Cao, Li & Żabka, 2016: 54, figs 7A–D, 8A, B (holotype ♂, not examined).
Bavirecta exilis : Kanesharatnam and Benjamin 2018: 8 (transferred from Bavia).
Padillothorax exilis : Maddison et al. 2020: 65 (transferred from Bavirecta).

##### Material examined.

1 ♂ 1 ♀ (TRU-JS 0759–0760), China: • Hainan Province, Qiongzhong County, Limushan National Nature Reserve (19°9.35'N, 109°44.70'E, ca 620 m), 6.VIII.2023, C. Wang et al. leg.

##### Diagnosis.

**Figure 18.**
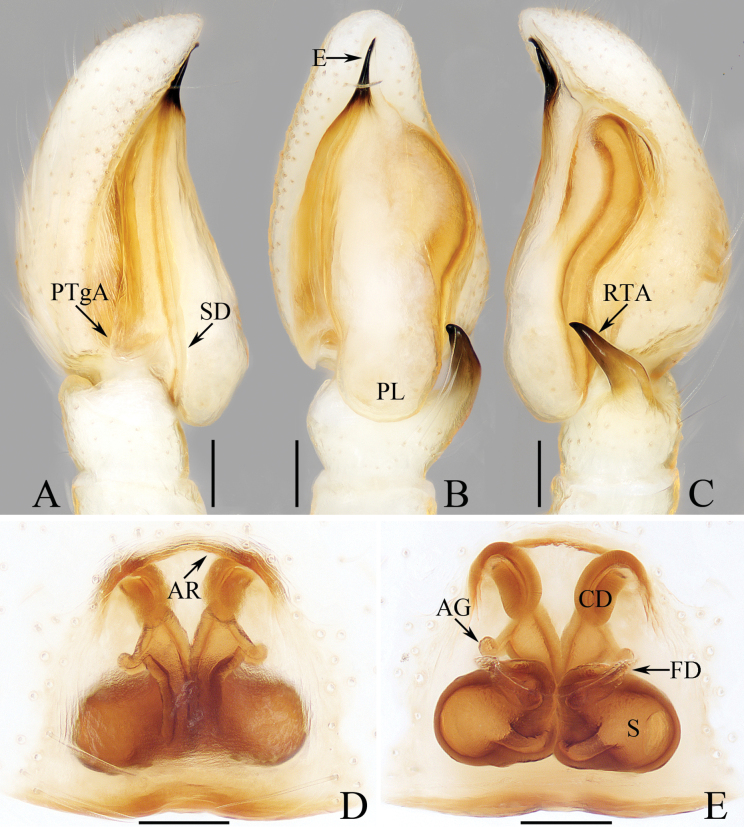
Copulatory organs of *Padillothoraxexilis* (Cao & Li, 2016) **A–C** male palp (TRU-JS 0759) and **D, E** epigyne (TRU-JS 0760) **A** prolateral **B** ventral **C** retrolateral **D** epigyne, ventral **E** vulva, dorsal. Abbreviations: AG accessory gland; AR atrial ridge; CD copulatory duct; E embolus; FD fertilization duct; PL posterior tegular lobe; PTgA prolateral tegular apophysis; RTA retrolateral tibial apophysis; S spermatheca; SD sperm duct. Scale bars: 0.1 mm.

The male was diagnosed in Cao et al. (2016). The female resembles that of *P.casteti* (Simon, 1900) in the general shape of epigyne, but differs in: 1) presence of accessory glands (AG) of copulatory ducts (Fig. 18D, E) vs absent (see the drawings in Prószyński 1987: 78); 2) copulatory ducts (CD) curved distally and connected to the dorsal surface of spermathecae (S) (Fig. 18E) vs straight distally and connected to the ventral surface of spermathecae (see the drawings in Prószyński 1987: 78).

##### Description.

**Male.** See Cao et al. (2016).

**Figure 19.**
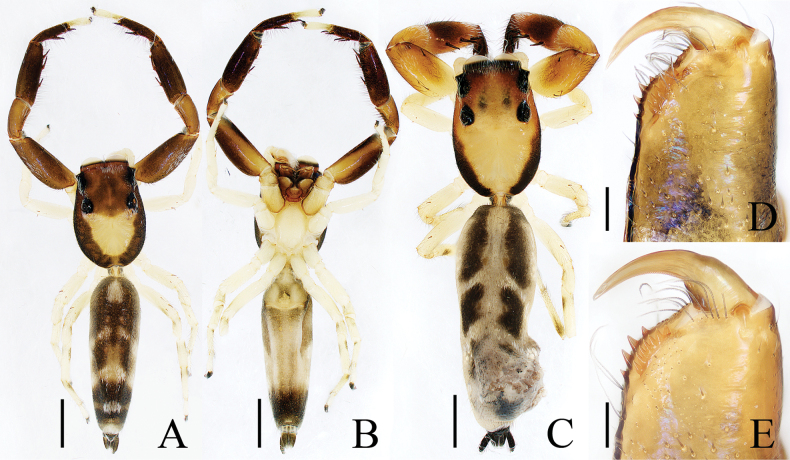
*Padillothoraxexilis* (Cao & Li, 2016) **A, B, D** male (TRU-JS 0759) and **C, E** female (TRU-JS 0760) **A, C** habitus, dorsal **B** ditto ventral **D, E** chelicera, posterior. Scale bars: 1.0 mm (**A–C**); 0.1 mm (**D, E**).

**Female** (Figs 18D, E, 19C, E). Total length 5.36. Carapace 1.86 long, 1.32 wide. Abdomen 3.36 long, 1.23 wide. Eye sizes and interdistances: AME 0.45, ALE 0.18, PLE 0.18, AERW 1.14, PERW 1.05, EFL 0.77. Legs: I 5.24 (1.53, 0.93, 1.40, 0.88, 0.50), II 3.51 (1.00, 0.65, 0.85, 0.63, 0.38), III 3.18 (0.95, 0.60, 0.55, 0.70, 0.38), IV 4.36 (1.25, 0.65, 1.03, 1.05, 0.38). Carapace mainly yellow, with pair of dark stripes laterally on thoracic part, covered with sparse setae, denser on eye base. Chelicerae yellow, with four promarginal and seven retromarginal teeth. Leg I robust, with thickened femora, three and two pairs of ventral spines on tibiae and metatarsi, respectively. Dorsum of abdomen with symmetrical, alternating pale and dark patches; venter pale.

***Epigyne*** (Fig. 18D, E) ~ 1.3× wider than long; atrium (At) with anterior arc-shaped ridge (AR), copulatory openings (CO) slit-shaped, partly covered by atrial ridge; copulatory ducts (CD) thickened in walls at proximal 1/3, strongly curved distally, and with medially located, laterally extended accessory glands (AG) forming round ends; spermathecae (S) oval, touching each other.

##### Distribution.

China (Yunnan, Hainan; Fig. 48).

#### ﻿Genus Pancorius

Taxon classificationAnimaliaAraneaeSalticidae

Simon, 1902

528DE5E4-3804-53D9-8F88-83F4B5D8D530

##### Type species.

*Erganedentichelis* Simon, 1899; type locality Padang, Indonesia.

##### Comments.

*Pancorius* is placed in the subtribe *Plexippina* Simon, 1901 within the tribe *Plexippini* Simon, 1901 (Maddison 2015), and comprises 46 species restricted to Asia (WSC 2024). The genus is poorly studied and its generotype is only known from limited diagnostic drawings, resulting in it not being precisely delimited. In addition, members are rather diverse in habitus and copulatory organs, and several species, such as *P.guiyang* Yang, Gu & Yu, 2023, *P.inexpectatus* Logunov, 2024, *P.lui* Gan, Mi & Wang, 2022, and *P.nyingchi* Wang, Mi & Li, 2024 were tentatively placed, indicating that they could be polyphyletic. Moreover, half its species are only known from a single sex (WSC 2024).

#### ﻿ Pancorius medog sp. nov.

Taxon classificationAnimaliaAraneaeSalticidae

EA7FFB9D-0647-5D0A-B1EE-467122C12558

https://zoobank.org/60603398-7E09-433D-ADF6-1F078E7D4B96

Figs 20 , 47

##### Type material.

***Holotype*** ♀ (TRU-JS 0761), China: • Xizang Autonomous Region, Medog County, Beibeng Township, Deergong Village, Yarlung Zangbo National Nature Reserve (29°10.84'N, 95°8.67'E, ca 1670 m), 25.V.2024, X.Q. Mi et al. leg. ***Paratypes*** • 3 ♀ (TRU-JS 0762–0764), same data as for holotype.

##### Etymology.

The specific name is named after the type locality, Medog County; noun in apposition.

##### Diagnosis.

**Figure 20.**
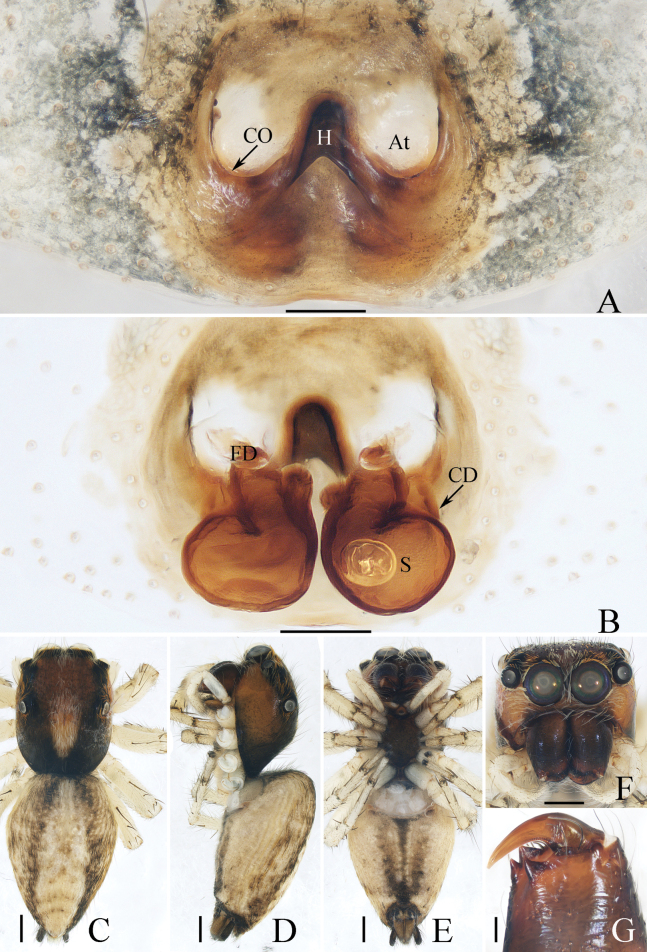
*Pancoriusmedog* sp. nov., holotype **A** epigyne, ventral **B** vulva, dorsal **C** habitus, dorsal **D** ditto, lateral **E** ditto, ventral **F** carapace, frontal **G** chelicera, posterior. Abbreviations: At atrium; CO copulatory opening; CD copulatory duct; FD fertilization duct; H epigynal hood; S spermatheca. Scale bars: 0.1 mm (**A, B, G**); 0.5 mm (**C–F**).

*Pancoriusmedog* sp. nov. resembles that of *P.nyingchi* Wang, Mi & Li, 2024 in having a central epigynal hood (H), longitudinal band on the dorsum of abdomen, but can be easily distinguished by the following: 1) epigynal hood opened posteriorly (Fig. 20A) vs opened ventro-posteriorly (Wang et al. 2024: fig. 12A); 2) the distinct spermathecae (S) (Fig. 20B) vs indistinct (Wang et al. 2024: fig. 12B); 3) presence of central yellow area bearing pale thin setae on carapace (Fig. 20C) vs absent (Wang et al. 2024: fig. 12E).

##### Description.

**Female** (Fig. 20). Total length 5.19. Carapace 2.31 long, 1.78 wide. Abdomen 3.01 long, 2.03 wide. Eye sizes and interdistances: AME 0.52, ALE 0.27, PLE 0.25, AERW 1.67, PERW 1.67, EFL 1.03. Legs: I 4.08 (1.25, 0.75, 1.00, 0.63, 0.45), II 3.72 (1.13, 0.70, 0.88, 0.58, 0.43), III 4.18 (1.40, 0.63, 0.90, 0.80, 0.45), IV 4.72 (1.45, 0.68, 1.08, 1.03, 0.48). Carapace orange-brown on cephalon and dark on thoracic part, with central yellow area bearing pale thin setae, covered with pale, dark brown and golden setae. Chelicerae red-brown, with two promarginal teeth and one retromarginal tooth. Legs pale, spiny. Dorsum of abdomen grey-brown, with longitudinal, sub-fusiform central stripe extended across whole surface; venter mainly pale brown, with central, longitudinal, non-consecutive, dark patches.

***Epigyne*** (Fig. 20A, B) longer than wide, with central, posteriorly opened hood (H) with inverted V-shaped margin; atrium (At) sub-square, located anteriorly; copulatory openings (CO) slit-shaped; copulatory ducts (CD) short, curved into U-shape and then folded to connect to antero-inner portions of spermathecae; spermathecae (S) almost spherical, with anterior extended extensions.

**Male.** Unknown.

##### Distribution.

Known only from the type locality in Xizang, China (Fig. 47).

#### ﻿ Pancorius yingjiang sp. nov.

Taxon classificationAnimaliaAraneaeSalticidae

6D15C05E-C674-56A7-9813-BED89D1B0777

https://zoobank.org/C4E290D2-5E94-4C5B-8DF3-A14C58250BFD

Figs 21 , 22 , 48

##### Type material.

***Holotype*** ♀ (TRU-JS 0765), China: • Yunnan Province, Dehong Dai Autonomous Prefecture, Yingjiang County, Tongbiguan Township, Banggetong (24°35.96'N, 97°38.48'E, elevation undetailed) 3.V.2024, H. Qiu leg. ***Paratypes*** • 3 ♂ (TRU-JS 0766–0768), same data as for holotype.

##### Etymology.

The species name comes from the type locality, Yingjiang County; noun in apposition.

##### Diagnosis.

**Figure 21.**
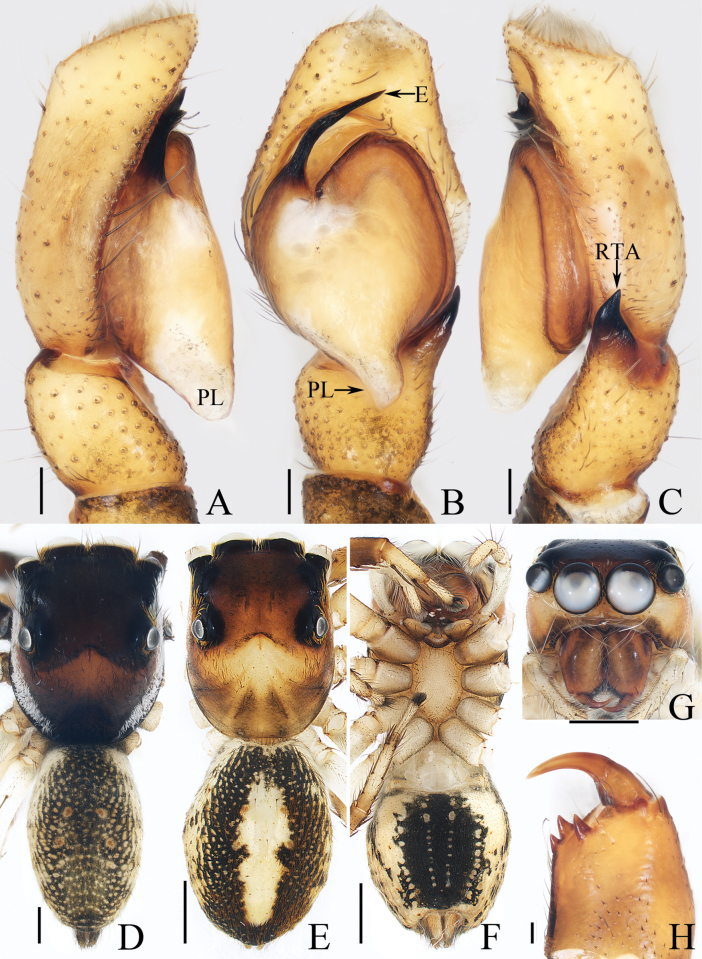
*Pancoriusyingjiang* sp. nov. **E–H** female holotype and **A–D** male paratype (TRU-JS 0766) **A** palp, prolateral **B** ditto, ventral **C** ditto, retrolateral **D, E** hatitus, dorsal **F** ditto, ventral **G** carapace, frontal **H** chelicera, posterior. Abbreviations: E embolus; PL posterior tegular lobe; RTA retrolateral tibial apophysis. Scale bars: 0.5 mm (**D, G**); 1.0 mm (**E, F**); 0.1 mm (**A–C, H**).

**Figure 22.**
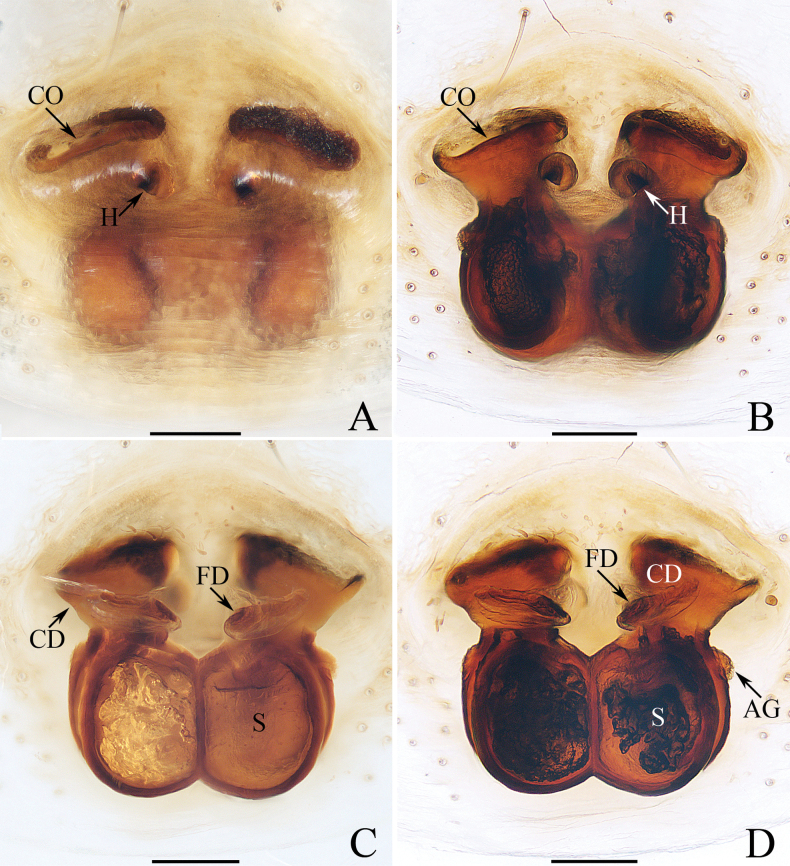
Epigyne of *Pancoriusyingjiang* sp. nov., female holotype **A, B** epigyne, ventral **C, D** vulva, dorsal. Abbreviations: AG accessory gland; CO copulatory opening; CD copulatory duct; FD fertilization duct; H epigynal hood; S spermatheca. Scale bars: 0.1 mm.

*Pancoriusyingjiang* sp. nov. resembles that of *P.manipuriensis* (Biswas & Biswas, 2004) in having a similar male palp and a small, anteriorly located epigynal hood (H), but can be easily distinguished by the following: 1) copulatory openings (CO) opened anteriorly (Fig. 22A, B) vs opened opposite (Caleb 2023: figs 21, 22); 2) epigynal hoods (H) posterior to copulatory openings (CO) (Fig. 22A, B) vs lateral to copulatory openings (Caleb 2023: figs 21, 22); 3) retrolateral tibial apophysis (RTA) directed towards ca 12 o’clock position in ventral view (Fig. 21B) vs ca 2 o’clock position (Caleb 2023: fig. 15).

##### Description.

**Female** (Figs 21E–H, 22). Total length 6.40. Carapace 3.03 long, 2.40 wide. Abdomen 3.23 long, 2.43 wide. Eye sizes and interdistances: AME 0.72, ALE 0.43, PLE 0.38, AERW 2.30, PERW 2.20, EFL 1.40. Legs: I 6.16 (1.88, 1.18, 1.50, 1.00, 0.60), II 5.56 (1.63, 1.10, 1.25, 0.95, 0.63), III 6.61 (2.13, 1.00, 1.45, 1.35, 0.68), IV 6.97 (2.13, 0.98, 1.55, 1.63, 0.68). Carapace yellow-brown, covered with dark golden and pale setae, with irregular yellow area anteriorly on thoracic part. Chelicerae brawny, with two promarginal teeth and one retromarginal tooth. Legs pale, mingled with red-brown, spiny. Dorsum of abdomen dark and spotted laterally, with central, longitudinal, pale stripe and two pairs of median muscle depressions; venter dark centrally, with pair of dotted lines.

***Epigyne*** (Fig. 22A–D) with pair of anterior, small hoods (H) below copulatory openings (CO); copulatory openings slit-shaped, opened anteriorly, and apart from each other < 1/2 their width; copulatory ducts (CD) broad, with small, mediolateral accessory glands (AG); spermathecae (S) almost round, touching each other.

**Male** (Fig. 21A–D). Total length 5.23. Carapace 2.66 long, 2.14 wide. Abdomen 2.63 long, 1.60 wide. Eye sizes and interdistances: AME 0.66, ALE 0.40, PLE 0.37, AERW 2.06, PERW 1.97, EFL 1.26. Legs: I 6.84 (2.00, 1.08, 1.78, 1.25, 0.73), II 5.64 (1.75, 0.90, 1.33, 1.03, 0.63), III 6.54 (2.08, 0.90, 1.38, 1.45, 0.73), IV 6.82 (2.03, 0.85, 1.43, 1.58, 0.93). Habitus (Fig. 21D) similar to that of female except carapace darker, and without central, longitudinal, pale stripe on dorsum of abdomen.

***Palp*** (Fig. 21A–C): femur length/width ratio ca 3.34; patella ~ 1/2 femoral length; tibia slightly longer than wide, with strongly sclerotized, tapered retrolateral apophysis (RTA) curved distally and with pointed tip; cymbium ~ 1.5× longer than wide; tegulum slightly swollen posteriorly, with well-developed posterior lobe (PL) with blunt end; embolus (E) arising from anteroprolateral portion of tegulum, with median sub-triangular extension and pointed end.

##### Distribution.

Known only from the type locality in Yunnan, China (Fig. 48).

##### Comments.

As the female can be more easily distinguished from other congeners than the male, it is proposed as the holotype.

#### ﻿Genus Piranthus

Taxon classificationAnimaliaAraneaeSalticidae

Thorell, 1895

08576105-6096-58ED-B710-9CE2E0DA0970

##### Type species.

*Piranthusdecorus* Thorell, 1895; type locality Palon, Myanmar.

##### Comments.

*Piranthus* is considered as a member of the tribe Baviini Simon, 1901 (Maddison 2015; Maddison et al. 2020). To date, six species are known from tropical Asia (WSC 2024), of which four were described by Maddison et al. (2020). The genus is relatively well studied because all members are known from diagnostic drawings, and only two are known from a single sex (WSC 2024). Besides the below-described new species, the generotype has also been found in Hainan, China.

#### ﻿ Piranthus maddisoni sp. nov.

Taxon classificationAnimaliaAraneaeSalticidae

DD899E13-30C3-5708-9B17-21C4CBCAD732

https://zoobank.org/62C62771-5D33-41E5-AB29-F0831674E7D7

Figs 23 , 24 , 47

##### Type material.

***Holotype*** ♂ (TRU-JS 0769), China: • Hainan Province, Changjiang Li Autonomous County, Bawangling National Nature Reserve (19°7.12'N, 109°9.34'E, ca 640 m), 24.IV.2021, F.E. Li leg. ***Paratype*** • 1 ♀ (IZCAS-Ar 45283), Lingshui County, Diaoluoshan (18°40.22'N, 109°53.67'E, ca 260 m), 14.IV.2009, G. Tang leg.

##### Etymology.

The specific name is a patronym in honor of Prof. Wayne P. Maddison (Vancouver, Canada), the leading specialist in jumping spiders, who has made significant contributions to the taxonomy of salticids worldwide; noun (name) in the genitive case.

##### Diagnosis.

**Figure 23.**
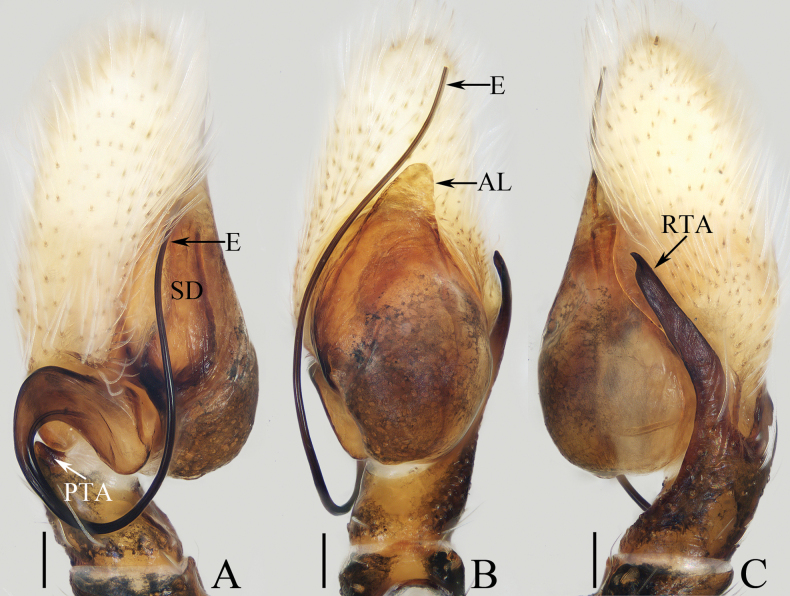
Male palp of *Piranthusmaddisoni* sp. nov., holotype **A** prolateral **B** ventral **C** retrolateral. Abbreviations: AL anterior tegular lobe; E embolus; PTA prolateral tibial apophsis; RTA retrolateral tibial apophysis; SD sperm duct. Scale bars: 0.1 mm.

**Figure 24.**
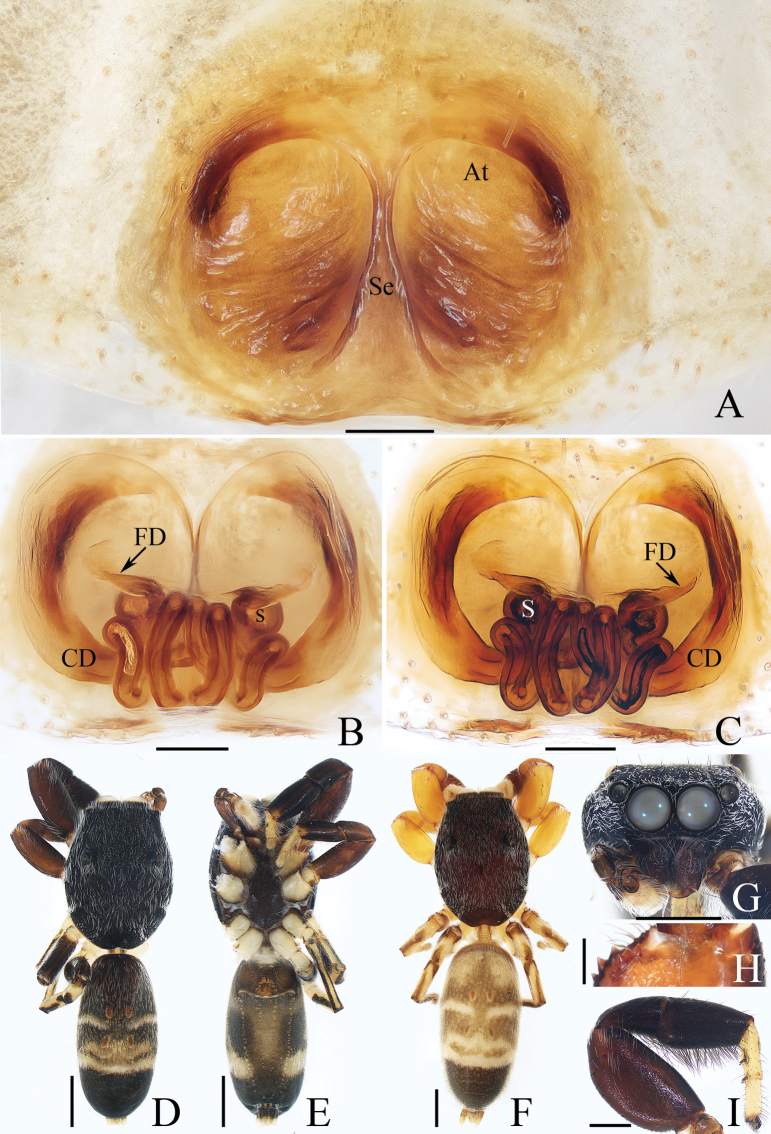
*Piranthusmaddisoni* sp. nov. **D, E, G–I** male holotype and **A–C, F** female paratype (IZCAS-Ar 45283) **A** epigyne, ventral **B, C** vulva, dorsal **D**, **F** habitus, dorsal **E** ditto, ventral **G** carapace, frontal **H** chelicera, posterior **I** leg I, prolateral. Abbreviations: At atrium; CD copulatory duct; FD fertilization duct; S spermatheca; Se septum. Scale bars: 0.1 mm (**A–C, H**); 1.0 mm (**D–G**); 0.5 mm (**I**).

*Piranthusmaddisoni* sp. nov. resembles that of *P.bakau* Maddison, 2020 in having similar habitus, pattern, and palpal structure, but differs in: 1) retrolateral tibial apophysis (RTA) not broadened at base, and forming an incision at distal end in retrolateral view (Fig. 23C) vs broadened into a dorsal prominent portion and lacking similar incision (Maddison et al. 2020: fig. 239); 2) presence of a well-developed anterior tegular lobe (AL) (Fig. 23B) vs indistinct (Maddison et al. 2020: fig. 238); 3) base of septum (Se) < 1/4 of epigynal width (Fig. 24A) vs ~ 1/3 of epigynal width (Maddison et al. 2020: fig. 240).

##### Description.

**Male** (Figs 23, 24D, E, G–I). Total length 6.10. Carapace 2.78 long, 2.15 wide. Abdomen 3.27 long, 1.61 wide. Eye sizes and interdistances: AME 0.55, ALE 0.26, PLE 0.26, AERW 1.51, PERW 1.59, EFL 1.12. Legs: I 5.25 (1.75, 1.00, 1.25, 0.75, 0.50), II 4.51 (1.38, 0.90, 1.10, 0.68, 0.45), III 3.76 (1.13, 0.70, 0.70, 0.80, 0.43), IV 5.04 (1.58, 0.75, 1.18, 1.08, 0.45). Carapace almost oval, dark, and covered with dense white setae. Chelicerae red-brown, with three promarginal and five retromarginal teeth. Legs I and II yellow except thickness femora, patellae, and tibiae dark brown, bearing pale setae on patellae and tibiae I. Dorsum of abdomen with two pairs of muscle depressions, pair of transverse, pale setal stripes followed by big transverse pale grey band medially, covered by anterior scutum ~ 1/3 abdominal length; venter colored as dorsum, with pair of oval postero-lateral pale spots and median dotted lines.

***Palp*** (Fig. 23A–C): femur length/width ratio ca 2.5; patella ~ 1.4× longer than wide in retrolateral view; tibia slightly longer than wide, with sub-triangular disto-prolateral apophysis (PTA) and blade-shaped retrolateral apophysis (RTA) longer than tibia, slightly curved at proximal 1/3, and forming shallow incision at distal end; cymbium pale, > 1.5× longer than wide; tegulum elongate-oval, swollen medio-posteriorly, with lamellar, anteriorly extended antero-marginal sub-triangular lobe (AL); embolus (E) arising at baso-prolateral corner of tegulum, with broad base extended anticlockwise, and then acutely narrowed into flagelliform portion.

**Female** (Fig. 24A–C, F). Total length 8.34. Carapace 3.47 long, 2.53 wide. Abdomen 4.53 long, 2.20 wide. Eye sizes and interdistances: AME 0.43, ALE 0.26, PLE 0.26, AERW 1.48, PERW 1.60, EFL 1.05. Legs: I 4.89 (1.65, 1.03, 1.08, 0.63, 0.50), II 4.83 (1.50, 1.05, 1.15, 0.68, 0.45), III 4.24 (1.30, 0.80, 0.78, 0.93, 0.43), IV 6.00 (1.80, 1.00, 1.40, 1.30, 0.50). Habitus (Fig. 24F) similar to that of male except paler and without dorsal abdominal scutum.

***Epigyne*** (Fig. 24A–C) ~ 1.2× wider than long; atrium (At) almost oval, separated by basally broadened septum (Se); copulatory openings (CO) anteriorly located, partly visible; copulatory ducts (CD) long, broadened and flat proximally, and then forming complicated coils; spermathecae (S) spherical, separated from each other ~ 2× their diameter.

##### Distribution.

Known only from the type locality in Hainan, China (Fig. 47).

##### Comments.

Although the male and female were collected in different places, they share consistent habitus, and pattern and thus they are considered to be conspecific, but this may need further confirmation.

#### ﻿Genus Siler

Taxon classificationAnimaliaAraneaeSalticidae

Simon, 1889

E4DD64D0-F95B-57AE-8097-C261BA05F719

##### Type species.

*Silercupreus* Simon, 1889; type locality Yokohama, Japan.

##### Comments.

*Siler*, a member of Chrysillini, comprises 12 species, mainly distributed in east and southeast Asia (Maddison 2015; WSC 2024). The genus has not been revised recently. Like most salticid genera, a high rate (58.3%) of its species are known only from a single sex. In addition, three species are only known from the original description, and *S.pulcher* Simon, 1901 has never been illustrated (WSC 2024).

#### ﻿ Siler hanoicus

Taxon classificationAnimaliaAraneaeSalticidae

Prószyński, 1985

4D00E460-470F-55DD-ADE8-42FA35030620

Figs 25 , 26 , 48

Siler hanoicus Prószyński, 1985: 75, figs 21, 22 (holotype ♂, not examined); Żabka, 1985: 447, figs 571, 572 (♂).

##### Material examined.

1 ♂ 1 ♀ (TRU-JS 0770–0771), China: • Guangxi Zhuang Autonomous Region, Fangchenggang City, Shiwandashan National Nature Reserve, west border of Pinglong Station (21°50.73'N, 107°53.24'E, ca 430 m), 30.IV.2024, A.L. He et al. leg.

##### Diagnosis.

**Figure 25.**
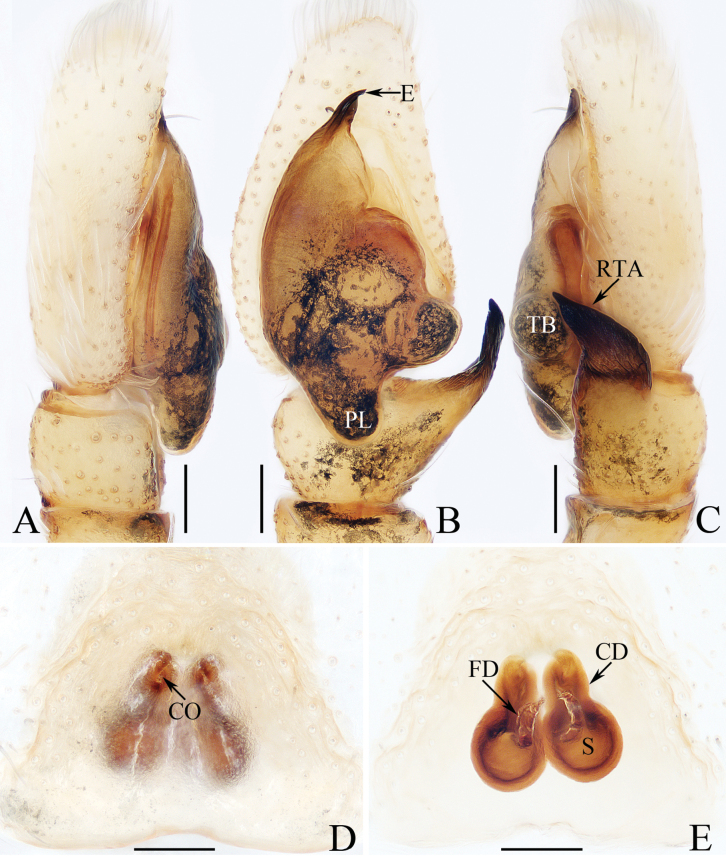
Copulatory organs of *Silerhanoicus* Prószyński, 1985 **A–C** male palp (TRU-JS 0770) and **D, E** eigyne (TRU-JS 0771) **A** prolateral **B** ventral **C** retrolateral **D** epigyne, ventral **E** vulva, dorsal. Abbreviations: CD copulatory duct; CO copulatory opening; E embolus; FD fertilization duct; PL posterior tegular lobe; RTA retrolateral tibial apophysis; S spermatheca; TB tegular bump. Scale bars: 0.1 mm.

*Silerhanoicus* resembles that of *S.cupreus* in the general shape of copulatory organs but differs in: 1) embolus (E) curved (Fig. 25B) vs straight (Peng 2020: fig. 296b); 2) retrolateral tibial apophysis (RTA) curved ventrally in retrolateral view (Fig. 25C), vs anteriorly extending before curved ventrally (Peng 2020: fig. 296b); 3) presence of a pair of atrial ridges (AR) (Fig. 25D), vs absent (Peng 2020: fig. 296f).

##### Re-description.

**Figure 26.**
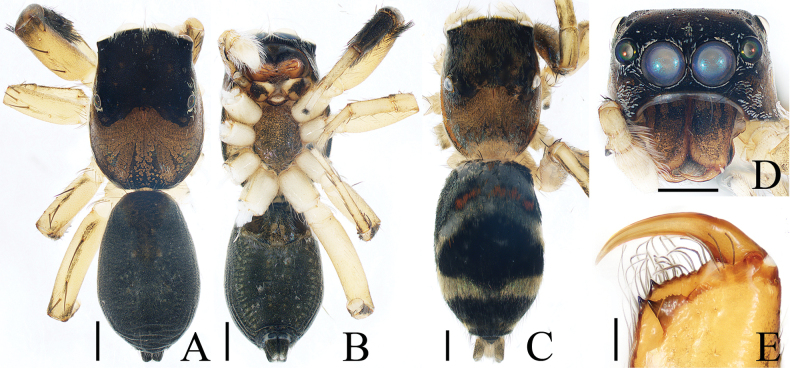
*Silerhanoicus* Prószyński, 1985 **A, B, D, E** male (TRU-JS 0770) and **C** female (TRU-JS 0771) **A, C** habitus, dorsal **B** ditto, ventral **D** carapace, frontal **E** chelicera, posterior. Scale bars: 0.5 mm (**A–D**); 0.1 mm (**E**).

**Male** (Figs 25, 26A, B, D, E). Total length 4.09. Carapace 2.03 long, 1.49 wide. Abdomen 2.11 long, 1.37 wide. Eye sizes and interdistances: AME 0.41, ALE 0.21, PLE 0.19, AERW 1.16, PERW 1.37, EFL 0.94. Legs: I 4.16 (1.35, 0.70, 0.95, 0.73, 0.43), II 3.23 (1.00, 0.50, 0.75, 0.63, 0.35), III 4.07 (1.13, 0.48, 1.18, 0.85, 0.43), IV (1.50, missing, missing, missing, missing). Carapace brown except cephalon dark, covered with pale scales on face and around PMEs; fovea red. Chelicerae yellow, mingled with dark, with two promarginal teeth and one retromarginal tooth. Legs yellow except tibiae I dark, covered with dense, dark, ventral setae on patellae and tibiae I. Dorsum of abdomen dark, mingled with green, with irregular anterior scutum ~ 1/3 abdominal length; venter colored as dorsum.

***Palp*** (Fig. 25A–C): femur length/width ratio ca 2.5; patella ~ 1.2× longer than wide in retrolateral view; tibia ~ as long as wide in retrolateral view; retrolateral tibial apophysis (RTA) strongly sclerotized, curved ventrally to rather pointed tip in retrolateral view; cymbium pale yellow, ~ 1.6× longer than wide; tegulum length/width ratio ca 1.53, with posteriorly extended posterior lobe (PL) with blunt end, and sub-spherical retrolateral tegular bump (TB); embolus (E) originating from most anterior portion of tegulum, slightly curved, and with blunt end.

**Female** (Figs 25D, E, 26C). Total length 5.14. Carapace 2.29 long, 1.67 wide. Abdomen 2.90 long, 1.81 wide. Eye sizes and interdistances: AME 0.43, ALE 0.26, PLE 0.26, AERW 1.48, PERW 1.60, EFL 1.05. Legs: I 4.61 (1.55, 0.68, 1.10, 0.83, 0.45), II 3.91 (1.25, 0.63, 0.88, 0.75, 0.40), III 4.64 (1.38, 0.63, 1.00, 1.13, 0.50), IV 6.18 (1.68, 0.80, 1.50, 1.70, 0.50). Carapace (Fig. 26C) similar to that of male. Dorsum of abdomen (Fig. 26C) with inconsecutive, anterior, orange, arc-shaped setal stripes followed by alternate pale and dark setal patches; venter dark.

***Epigyne*** (Fig. 25D, E) sub-triangular; atrium (At) anteriorly located, with arc-shaped lateral ridges (AR); copulatory openings (CO) small, partly visible; copulatory ducts (CD) thick, posteriorly extended; spermathecae (S) touching each other, spherical.

##### Distribution.

China (Guangxi; Fig. 48); Vietnam (Hanoi).

#### ﻿Genus Simaetha

Taxon classificationAnimaliaAraneaeSalticidae

Thorell, 1881

688CF539-D68D-5DB4-99CE-82835361BCFE

##### Type species.

*Simaethathoracica* Thorell, 1881; type locality Australia.

##### Comments.

The genus was assigned by Maddison (2015) in the subtribe Simaethina Simon, 1903 within the tribe Viciriini Simon, 1901, and is represented by 23 nominal species, mainly distributed from South Asia to Australia (WSC 2024). Although a detailed revision of Oceanian species has been done by Żabka (1994), the genus remains poorly studied because nearly half (11) of the species are known only from a single sex, and four species cannot be precisely identified due to lack of diagnostic drawings (WSC 2024).

#### ﻿ Simaetha hainan sp. nov.

Taxon classificationAnimaliaAraneaeSalticidae

D9727390-4E18-5BA3-80E7-D6979E1FF064

https://zoobank.org/7FCE4F94-E617-42E1-AC89-FC806BB826A7

Figs 27 , 28 , 48

##### Type material.

***Holotype*** ♂ (IZCAS-Ar 45284), China: • Hainan Province, Lingshui County, Diaoluoshan National Nature Reserve (18°39.96'N, 109°35.81'E, ca 80 m), 15.IV.2009, G. Tang leg. ***Paratypes*** • 1 ♀ (IZCAS-Ar 45285), same data as for holotype; • 1 ♂ (IZCAS-Ar 45286), Diaoluoshan National Nature Reserve (18°40.44'N, 109°52.72'E, ca 580 m), 16.IV.2009, G. Tang leg; • 1 ♂ (IZCAS-Ar 45287), Diaoluoshan National Nature Reserve (18°40.44'N, 109°52.60'E, ca 490 m), 10.VIII.2010, G. Tang leg; • 1 ♀ (IZCAS-Ar 42288), Qiongzhong County, Yinggeling National Nature Reserve, Yinggezui Station (19°03.05'N, 109°33.75'E, ca 690 m), 25.VIII.2010, G. Zhou leg; • 2 ♂ (TRU-JS 0772–0773), Ledong County, Jianfeng Township, Jianfengling National Nature Reserve, Main Peak (18°43.11'N, 108°52.32'E, ca 1400 m), 16.IV.2019, C. Wang & Y.F. Yang leg.

##### Etymology.

The specific name is after the type locality, Hainan; noun in apposition.

##### Diagnosis.

**Figure 27.**
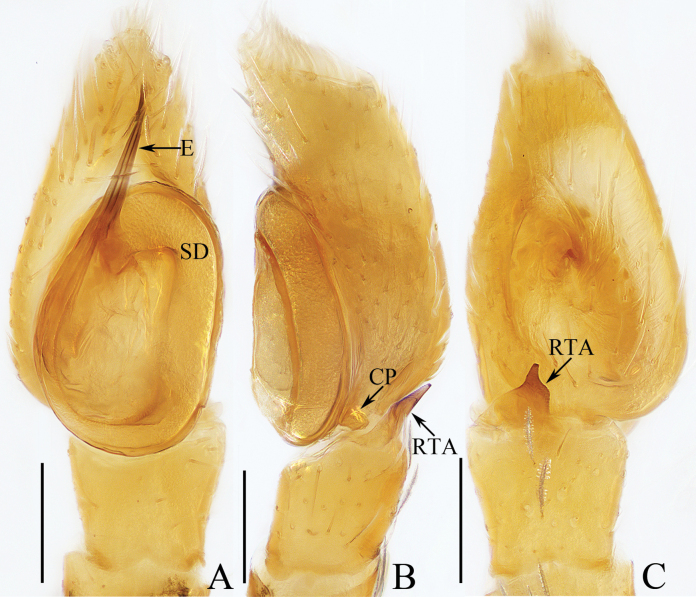
Male palp of *Simaethahainan* sp. nov., holotype **A** ventral **B** retrolateral **C** dorsal. Abbreviations: CP cymbial process; E embolus; RTA retrolateral tibial apophysis; SD sperm duct. Scale bars: 0.1 mm.

**Figure 28.**
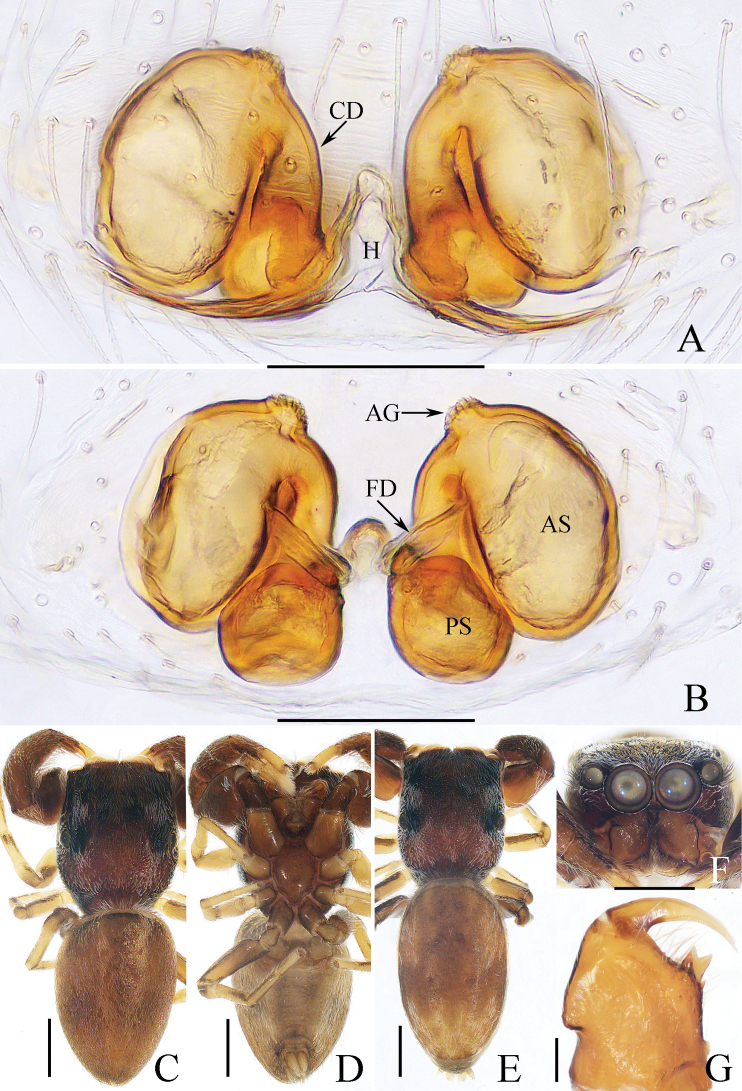
*Simaethahainan* sp. nov. **C, D, F, G** male holotype and **A, B, E** female paratype (IZCAS-Ar 45285) **A** epigyne, ventral **B** vulva, dorsal **C, E** habitus, dorsal **D** ditto, ventral **F** carapace, frontal **G** chelicera, anterior. Abbreviations: AG accessory gland; AS anterior chamber of spermatheca; CD copulatory duct; FD fertilization duct; H epigynal hood; PS posterior chamber of spermatheca. Scale bars: 0.1 mm (**A, B, G**); 0.5 mm (**C–F**).

*Simaethahainan* sp. nov. resembles that of *S.cheni* Wang & Li, 2021, in having the blade-shaped retrolateral tibial apophysis (RTA), the presence of antero-marginal protuberances on anterior surface of chelicerae, but differs in: 1) embolus (E) straight (Fig. 27A) vs curved prolaterally at distal portion (Wang and Li 2021: fig. 18B); 2) tibia slightly longer than wide in retrolateral view (Fig. 27B) vs wider than long (Wang and Li 2021: fig. 18C); 3) epigynal hood (H) posteriorly located, and approximately half the length of anterior chamber of spermatheca (Fig. 28A) vs anteriorly located and < 1/4 length of anterior chamber of spermatheca (Wang and Li 2021: fig. 19A, B).

##### Description.

**Male** (Figs 27, 28C, D, F, G). Total length 2.74. Carapace 1.32 long, 1.08 wide. Abdomen 1.50 long, 1.06 wide. Eye sizes and interdistances: AME 0.32, ALE 0.16, PLE 0.16, AERW 0.90, PERW 1.04, EFL 0.64. Legs: I 2.41 (0.78, 0.50, 050, 0.33, 0.30), II 1.76 (0.53, 0.30, 0.38, 0.30, 0.25), III 1.63 (0.50, 0.25, 0.33, 0.30, 0.25), IV 2.06 (0.73, 0.30, 0.45, 0.33, 0.25). Carapace red-brown, covered with pale and golden scales, with central dark patch on cephalon. Chelicerae red-yellow, with base-lateral protuberances on anterior surface, two promarginal teeth and one retromarginal fissidentate tooth with two cusps. Leg I robust, with enlarged femora, and three and two pairs of ventral spines on tibiae and metatarsi, respectively. Dorsum of abdomen red-brown, covered completely by large scutum; venter brown, with two pairs of dotted lines medially.

***Palp*** (Fig. 27A–C): femur length/width ratio ca 3.2; patella ~ 1.5× longer than wide in retrolateral view; tibia slightly longer than wide, with lamellar retrolateral apophysis (RTA) acutely narrowed at distal portion and blunt apically in dorsal view; cymbium ~ 1.8× longer than wide, with baso-retrolateral process (CP); tegulum oval; embolus (E) originating at ca 10:30 o’clock position, straight, tapered to rather blunt tip.

**Female** (Fig. 28A, B, E). Total length 3.16. Carapace 1.34 long, 1.07 wide. Abdomen 1.95 long, 1.08 wide. Eye sizes and interdistances: AME 0.32, ALE 0.17, PLE 0.17, AERW 0.97, PERW 1.08, EFL 0.74. Legs: I 2.11 (0.75, 0.45, 0.38, 0.28, 0.25), II 1.89 (0.58, 0.35, 0.38, 0.33, 0.25), III 1.68 (0.50, 0.25, 0.35, 0.33, 0.25), IV 2.24 (0.83, 0.28, 0.50, 0.38, 0.25). Habitus (Fig. 28E) similar to that of male except without base-lateral protuberances on anterior surface of chelicerae.

***Epigyne*** (Fig. 28A, B) ~ 1.7× wider than long, with posterior, sub-triangular hood (H) ~ 1/2 length of anterior chamber of spermatheca (AS); copulatory openings (CO) lateral to hood; copulatory ducts (CD) slightly curved medially, connected to antero-inner portions of anterior chamber of spermatheca, with small terminal accessory glands (AG); spermathecae (S) divided into oval anterior chamber extended posteriorly and spherical posterior chamber (PS); fertilization ducts (FD) arising from antero-inner portions of posterior chamber of spermatheca.

##### Distribution.

Known only from the type locality in Hainan, China (Fig. 48).

#### ﻿Genus Stertinius

Taxon classificationAnimaliaAraneaeSalticidae

Simon, 1890

E2CDC477-78C8-5D03-A82B-DAB8A997E2FB

##### Type species.

*Stertiniusdentichelis* Simon, 1890; type locality Mariana Is.

##### Comments.

*Stertinius*, is considered a member of Simaethina (Maddison 2015). Currently, 16 species have been placed in this genus, primarily from east and southeast Asia (WSC 2024). The genus is poorly defined because the generotype is lacking essential diagnostic drawings, and most of its species were assigned to the genus based only on the similarity to some of the known congeners (Wang et al. 2024).

#### ﻿ Stertinius lhoba sp. nov.

Taxon classificationAnimaliaAraneaeSalticidae

3EB0288C-D840-58A8-9297-23F56C104D60

https://zoobank.org/339A1A0E-A710-4B62-81AE-77367D097F6D

Figs 29 , 30 , 47

##### Type material.

***Holotype*** ♂ (TRU-JS 0774), China: • Xizang Autonomous Region, Medog County, Beibeng Township, Deergong Village, Yarlung Zangbo National Nature Reserve (29°10.84'N, 95°8.67'E, ca 1670 m), 25.V.2024, X.Q. Mi et al. leg. ***Paratypes*** • 2 ♀ (TRU-JS 0775–0776), same data as for holotype.

##### Etymology.

The specific name is after the Lhoba ethnic group, one of the two significant national minorities in Medog; noun in apposition.

##### Diagnosis.

**Figure 29.**
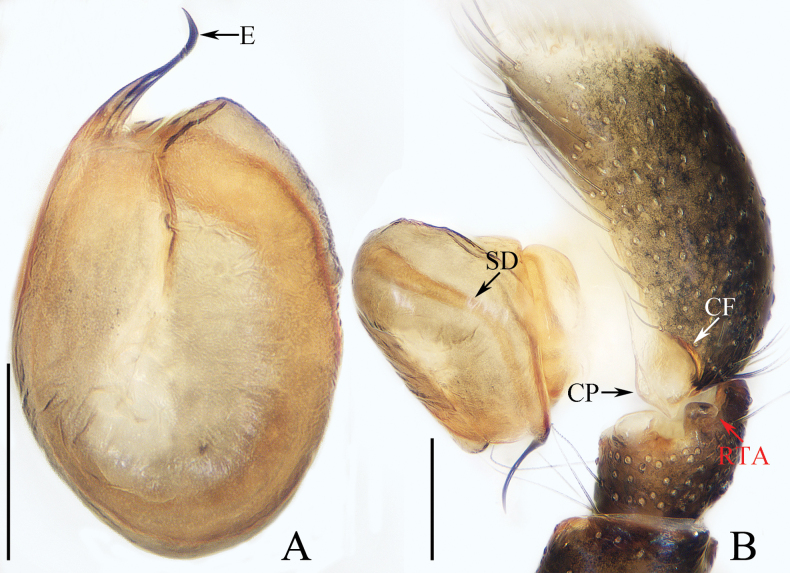
*Stertiniuslhoba* sp. nov., holotype **A** bulb, ventral **B** palp, retrolateral. Abbreviations: CF cymbial flange; CP cymbial process; E embolus; RTA retrolateral tibial apophysis; SD sperm duct. Scale bars: 0.1 mm.

**Figure 30.**
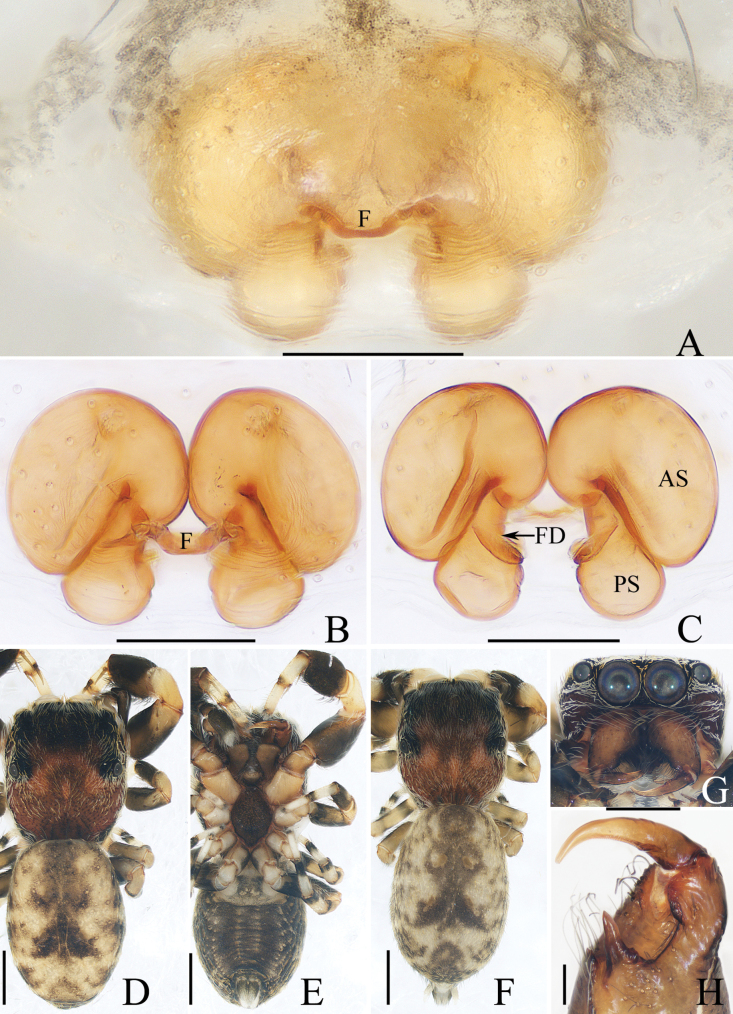
*Stertiniuslhoba* sp. nov. **D, E, G, H** male holotype and **A–C, F** female paratype (TRU-JS 0775) **A, B** epigyne, ventral **C** vulva, dorsal **D, F** habitus, dorsal **E** ditto, ventral **G** carapace, frontal **H** chelicera, posterior. Abbreviations: AS anterior chamber of spermatheca; F epigynal fold; FD fertilization duct; PS posterior chamber of spermatheca. Scale bars: 0.1 mm (**A–C, H**); 0.5 mm (**D–G**).

*Stertiniuslhoba* sp. nov. resembles that of *S.liqingae* Wang, Mi & Li, 2024 in general shape of copulatory organs, especially the epigyne structure, but differs in: 1) retrolateral tibial apophysis (RTA) almost equal in width in retrolateral view (Fig. 29B) vs almost tapered (Wang et al. 2024: fig. 15C); 2) epigyne has a fold (F) (Fig. 30A, B) vs a hood (Wang et al. 2024: fig. 16A, B); 3) anterior chamber of spermatheca (AS) almost posteriorly extending (Fig. 30B, C) vs transversely extending (Wang et al. 2024: fig. 16B, C).

##### Description.

**Male** (Figs 29, 30D, E, G, H). Total length 2.78. Carapace 1.28 long, 1.13 wide. Abdomen 1.60 long, 1.13 wide. Eye sizes and interdistances: AME 0.30, ALE 0.16, PLE 0.15, AERW 0.95, PERW 1.08, EFL 0.60. Legs: I 2.87 (0.90, 0.63, 0.68, 0.38, 0.28), II 2.04 (0.63, 0.38, 0.45, 0.30, 0.28), III 1.91 (0.60, 0.30, 0.40, 0.33, 0.28), IV 2.29 (0.75, 0.38, 0.50, 0.38, 0.28). Carapace mainly red-brown, covered with golden and pale setae, with central, irregular dark patch on cephalon. Chelicerae red-brown, with two promarginal teeth and one much larger pillar-shaped retromarginal tooth. Leg I robust, with enlarged femora and tibiae, and two pairs of ventral spines on tibiae and metatarsi. Dorsum of abdomen pale yellow, mingled with dark brown, covered wholly by scutum, with longitudinal, irregular central dark patch; venter dark with median dotted lines.

***Palp*** (Fig. 29A, B): femur length/width ratio ca 4.0; patella ~ 1.4× longer than wide in retrolateral view; tibia almost as long as wide in retrolateral view, with short retrolateral apophysis (RTA) slightly curved outwards and blunt apically; cymbium ~ 2× longer than tibia in ventral view, with sub-triangular baso-retrolateral process (CP); tegulum nearly oval, with sperm duct (SD) extending along submargin; embolus (E) originating from antero-prolateral portion of tegulum, slightly curved prolaterally at median portion and with pointed tip directed towards ca 11 o’clock position.

**Female** (Fig. 30A–C, F). Total length 2.72. Carapace 1.15 long, 1.01 wide. Abdomen 1.68 long, 1.10 wide. Eye sizes and interdistances: AME 0.30, ALE 0.16, PLE 0.15, AERW 0.90, PERW 1.01, EFL 0.58. Legs: I 1.94 (0.63, 0.40, 0.40, 0.28, 0.23), II 1.62 (0.50, 0.33, 0.33, 0.23, 0.23), III 1.59 (0.50, 0.28, 0.30, 0.28, 0.23), IV 2.23 (0.70, 0.45, 0.45, 0.38, 0.25). Habitus (Fig. 30F) similar to that of male except smaller retromarginal cheliceral tooth, and without dorsal abdominal scutum.

***Epigyne*** (Fig. 30A–C) ~ 1.46× wider than long, with sub-labiate central fold (F); copulatory openings (CO) small, beneath lateral portion of fold; copulatory ducts (CD) short, without distinct border; spermathecae (S) divided into oval anterior chamber (AS) and spherical posterior chamber (PS); fertilization ducts (FD) originating from antero-inner portions of posterior chamber of spermatheca.

##### Distribution.

Known only from the type locality in Xizang, China (Fig. 47).

#### ﻿Genus Synagelides

Taxon classificationAnimaliaAraneaeSalticidae

Strand, 1906

94D20DD4-1DAA-5299-9F81-3528FF9CFC6B

##### Type species.

*Synagelidesagoriformis* Strand, 1906; type locality Japan.

##### Comments.

*Synagelides* is placed in the tribe Agoriini Simon, 1901 (Maddison, 2015). To date, 78 nominal species have been described from east to southeast Asia, of which more than 60% are recorded from China (WSC 2024). The genus is relatively well studied because all its species are known from diagnostic drawings. However, > 41% of its species are only known from a single sex. The species described below are consistent in having hollowed fovea, two promarginal teeth and one retromarginal tooth on chelicerae, sub-triangular prolateral femoral apophysis (PFA), enlarged male palpal patella with a disto-prolateral bump (PB), and short male palpal tibia with a flat and broad ventral process (VTP).

#### ﻿ Synagelides kongmingi sp. nov.

Taxon classificationAnimaliaAraneaeSalticidae

102FADB3-58AA-53E1-81E7-3071823EF3BC

https://zoobank.org/2C8CCDDE-E317-4DD2-966E-626BC653C4DB

Figs 31 , 32 , 47

##### Type material.

***Holotype*** ♂ (TRU-JS 0777), China: • Sichuan Province, Bazhong City, Nanjiang County, Guangwu Township, Guangwushan-Nuoshuihe National Geopark (32°40.76'N, 106°46.11'E, ca 1010 m), 3.VI.2022, A.L. He et al. leg. ***Paratypes*** • 1 ♂ 2 ♀ (TRU-JS 0778–0780), Sandaoguan Scenic Area (32°39.57'N, 106°44.36'E, ca 1470 m), 4.VIII.2022, A.L. He et al. leg.

##### Etymology.

The specific name is a patronym in honor of a famous wise strategist Zhuge Kongming; noun (name) in the genitive case.

##### Diagnosis.

**Figure 31.**
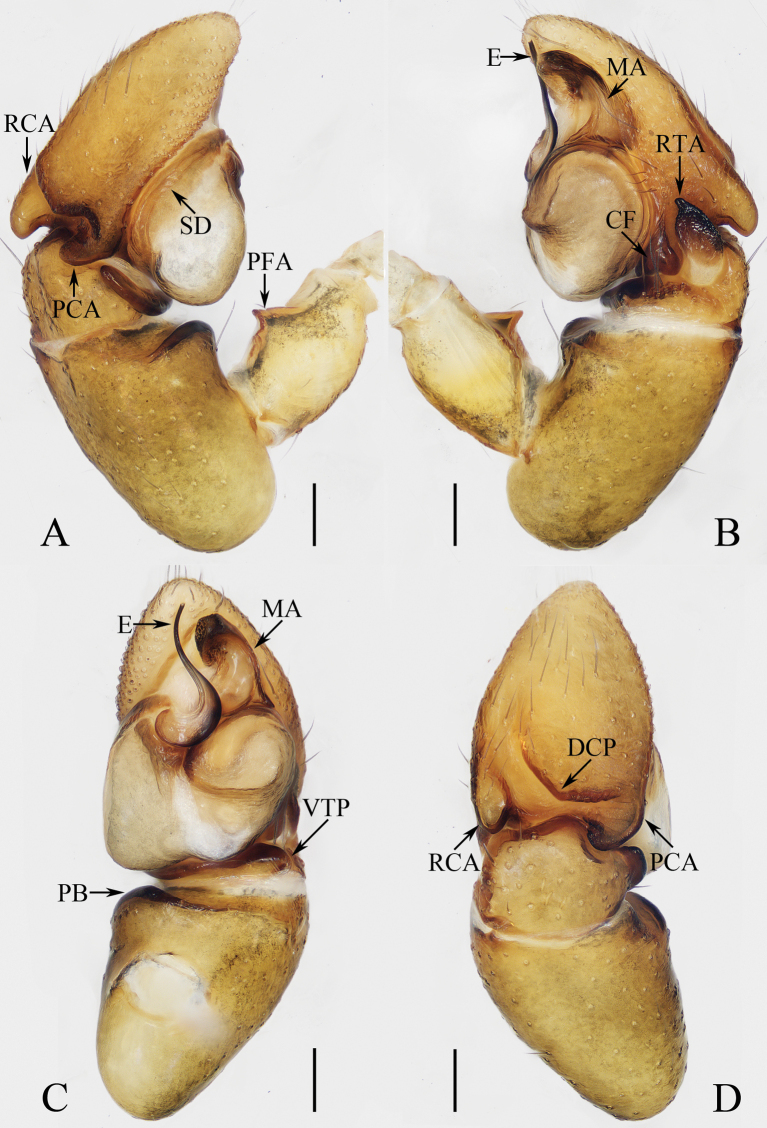
Male palp of *Synagelideskongmingi* sp. nov., paratype (TRU-JS 0778) **A** prolateral **B** retrolateral **C** ventral **D** dorsal. Abbreviations: CF cymbial flange; DCP dorsal cymbial process; E embolus; MA median apophysis; PB patellar bump of male palp; PCA prolateral cymbial apophysis; PFA prolateral femoral apophysis; RCA retrolateral cymbial apophysis; RTA retrolateral tibial apophysis; SD sperm duct; VTP ventral tibial process. Scale bars: 0.1 mm.

**Figure 32.**
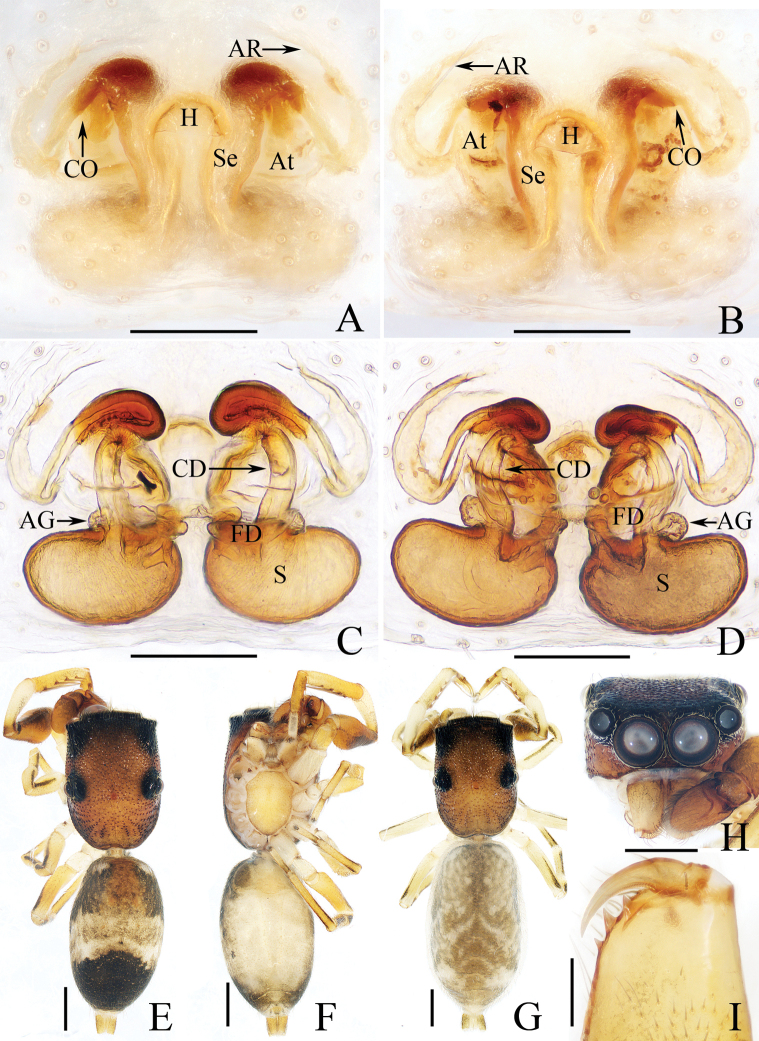
*Synagelideskongmingi* sp. nov. **E, F, H, I** holotype **A, C, G** female paratype (TRU-JS 0779) **B, D** female paratype (TRU-JS 0780) **A, B** epigyne, ventral **C, D** vulva, dorsal **E, G** habitus, dorsal **F** ditto, ventral **H** carapace, frontal **I** chelicera, posterior. Abbreviations: AG accessory gland; At atrium; AR atrial ridge; CD copulatory duct; CO copulatory opening; FD fertilization duct; H epigynal hood; S spermatheca; Se septum. Scale bars: 0.1 mm (**A–D, I**); 0.5 mm (**E–H**).

*Synagelideskongmingi* sp. nov. resembles that of *S.tianquan* Wang, Mi & Li, 2024 in having very similar habitus and copulatory organs, but differs in: 1) retrolateral cymbial apophysis (RCA) with a smooth edge in dorsal view (Fig. 31D) vs a shallow incision on inner edge (Wang et al. 2024: fig. 18D); 2) presence of a groove between the retrolateral cymbial apophysis and dorsal cymbial process (Fig. 31D) vs absent (Wang et al. 2024: fig. 18D); 3) spermathecae (S) transversely extending (Fig. 32C, D) vs anteriorly extending at lateral portions (Wang et al. 2024: fig. 19B); 4) accessory glands (AG) visible (Fig. 32C, D) vs invisible (Wang et al. 2024: fig. 19B).

##### Description.

**Male** (Figs 31, 32E, F, H, I). Total length 3.42. Carapace 1.53 long, 1.15 wide. Abdomen 1.84 long, 1.10 wide. Eye sizes and interdistances: AME 0.36, ALE 0.20, PLE 0.18, AERW 1.08, PERW 1.18, EFL 0.89. Legs: I 3.73 (1.18, 0.90, 0.90, 0.45, 0.30), II 2.54 (0.75, 0.43, 0.58, 0.50, 0.28), III 2.61 (0.75, 0.38, 0.60, 0.60, 0.28), IV 3.51 (1.00, 0.50, 0.88, 0.80, 0.33). Carapace mainly red-brown, covered with sparse, thin setae. Legs mainly yellow except enlarged femora I brown, with lateral stripes on femora, patellae, tibiae, and metatarsi II, III, IV, and four and two pairs of ventral spines on tibiae and metatarsi I, respectively. Dorsum of abdomen divided into brown, pale and dark portions, with pair of transverse, anterior, pale stripes bearing white setae, and longitudinal, central scutum extending through anterior 1/3; venter pale, without distinct markings.

***Palp*** (Fig. 31A–D): femur length/width ratio ca 1.8; patella ~ 1.5× longer than wide in retrolateral view; tibia ~ 1/3 patellar length, with flat retrolateral apophysis (RTA) abruptly narrowed distally to blunt tip directed ca 11 o’clock position; cymbium length/width ratio ca 1.6, with flat prolateral and horn-shaped retrolateral apophyses, as well as sheet-shaped dorsal process (DCP); tegulum swollen; median apophysis (MA) irregular, slightly bent towards ventrally at median portion; embolus (E) flat, and curved into invert C-shape at base, and followed by slightly curved, thinner, whip-shaped portion.

**Female** (Fig. 32A–D, G). Total length 3.42. Carapace 1.53 long, 1.15 wide. Abdomen 1.84 long, 1.10 wide. Eye sizes and interdistances: AME 0.36, ALE 0.20, PLE 0.18, AERW 1.08, PERW 1.18, EFL 0.89. Legs: I 3.33 (1.00, 0.75, 0.85, 0.43, 0.30), II 2.29 (0.65, 0.38, 0.53, 0.45, 0.28), III 2.54 (0.75, 0.38, 0.58, 0.55, 0.28), IV 3.44 (1.00, 0.45, 0.88, 0.78, 0.33). Carapace (Fig. 32G) similar to that of male except paler. Dorsum of abdomen (Fig. 32G) pale brown; venter pale.

***Epigyne*** (Fig. 32A–D) ~ 1.27× wider than long; atrium (At) occupies anterior 1/3, separated by broad, longitudinal septum (Se) grooved medio-posteriorly and bearing invert cup-shaped anterior hood (H), with pair of lateral arc-shaped ridges (AR) antero-laterally; copulatory openings (CO) invisible; copulatory ducts (CD) strongly curved at proximal 1/3, and connected to antero-inner portions of spermathecae, with short, transversely extended, terminal accessory glands (AG); spermathecae (S) oval, separated by ~ 1/8 of their width.

##### Distribution.

Known only from the type locality in Sichuan, China (Fig. 47).

#### ﻿ Synagelides xuandei sp. nov.

Taxon classificationAnimaliaAraneaeSalticidae

E600F30A-C5FE-5239-A5CA-51158A1E2DA2

https://zoobank.org/7B3C45E2-7239-462B-BB03-A92DBF9510BF

Figs 33 , 34 , 47

##### Type material.

***Holotype*** ♂ (TRU-JS 0781), China: • Guangxi Zhuang Autonomous Region, Laibing City, Jinxiu Yao Autonomous County, Yinshan Park (24°10.07'N, 110°14.48'E, ca 1310 m), 8.XI.2021, A.L. He et al. leg. ***Paratypes*** • 3 ♂ 7 ♀ (TRU-JS 0782–0791), same data as for holotype.

##### Etymology.

The specific name is after Mr. Liu Xuande, who is the first emperor of Shu during the Three Kingdoms of ancient China; noun (name) in the genitive case.

##### Diagnosis.

**Figure 33.**
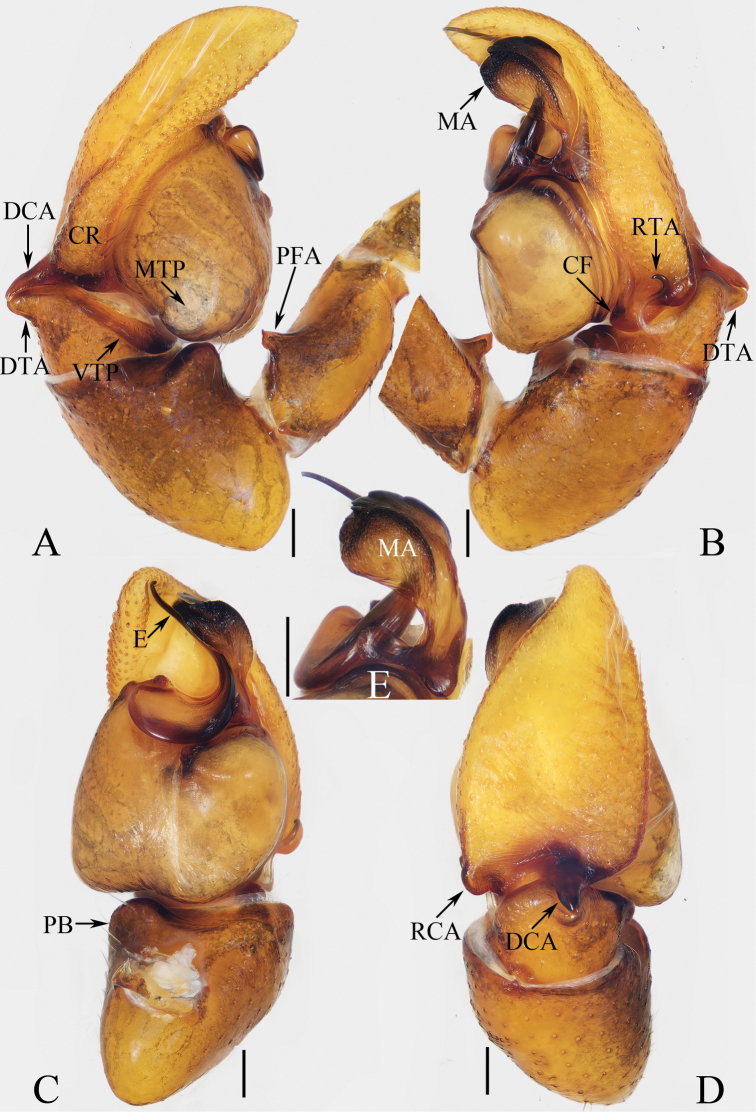
Male palp of *Synagelidesxuandei* sp. nov. **A–D** holotype and **E** paratype (TRU-JS 0782) **A** prolateral **B** retrolateral **C** ventral **D** dorsal **E** embolus and median apophysis, retrolateral. Abbreviations: CF cymbial flange; CR cymibal ridge; DCA dorsal cymbial apophysis; DTA dorsal tibial apophysis; E embolus; MA median apophysis; MTP membranous tegular peak; PB patellar bump of male palp; PFA prolateral femoral apophysis; RCA retrolateral cymbial apophysis; RTA retrolateral tibial apophysis; VTP ventral tibial process. Scale bars: 0.1 mm.

**Figure 34.**
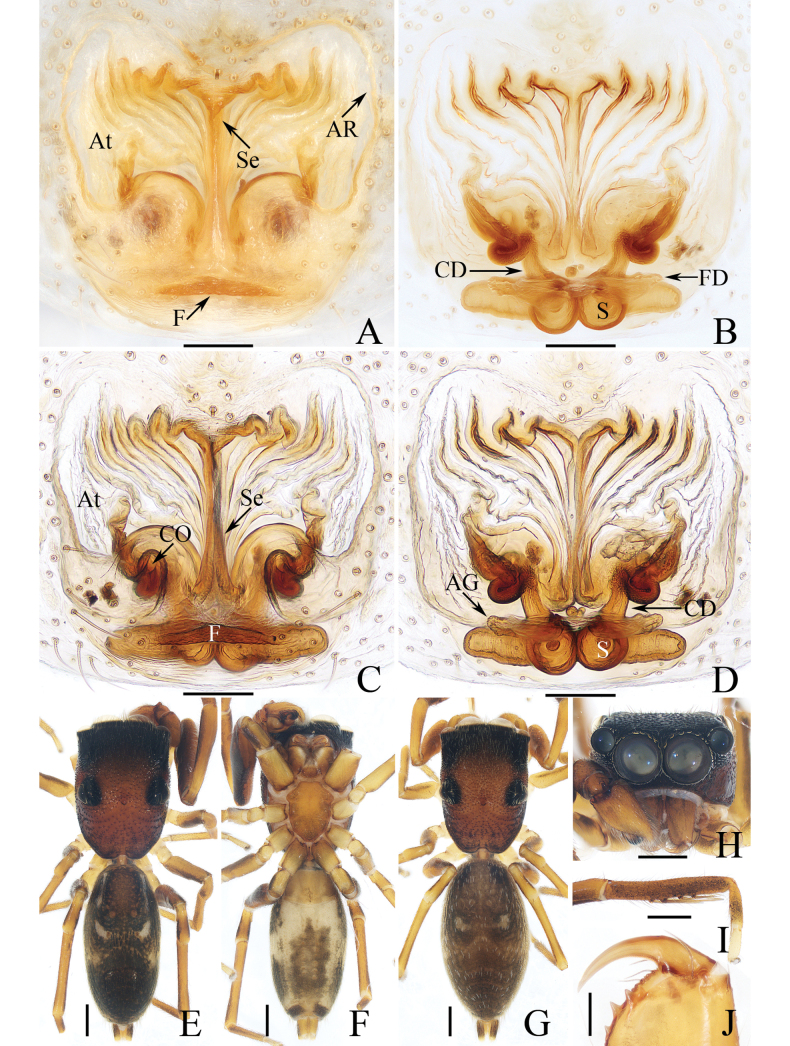
*Synagelidesxuandei* sp. nov. **E, F, H–J** holotype and **A–D, G** female paratype (TRU-JS 0785) **A, C** epigyne, ventral **B, D** vulva, dorsal **E, G** habitus, dorsal **F** ditto, ventral **H** carapace, frontal **I** tibia and metatarsi I, prolateral **J** chelicera, posterior. Abbreviations: AG accessory gland; AR atrial ridge; CD copulatory duct; CO copulatory opening; F epigynal fold; FD fertilization duct; S spermatheca; Se septum. Scale bars: 0.1 mm (**A–D, J**); 0.5 mm (**E–I)**.

The male of *Synagelidesxuandei* sp. nov. is similar to *S.huangxin* Lin & Li, 2024 in general shape of the palp, but can be distinguished by the median apophysis (MA), which is widest distally and has a base elongate-oval lamellar branch (Fig. 33B, E) vs almost tapered at distal half and lacking similar branch (Lin et al. 2024b: fig. 47B). The female of this species resembles that of *S.subgambosus* Wang, Mi, Irfan & Peng, 2020 in general shape of epigyne, especially the rugulose atrium (At), but can be easily distinguished the copulatory ducts (CD), which are strongly curved at proximal half (Fig. 34B–D) vs straight (Wang et al. 2020: fig. 12B, C).

##### Description.

**Male** (Figs 33, 34E, F, H–J). Total length 4.63. Carapace 2.07 long, 1.48 wide. Abdomen 2.52 long, 1.22 wide. Eye sizes and interdistances: AME 0.50, ALE 0.27, PLE 0.26, AERW 1.46, PERW 1.48, EFL 1.15. Legs: I 5.86 (1.85, 1.68, 1.45, 0.58, 0.30), II 3.72 (1.13, 0.58, 0.95, 0.73, 0.33), III 3.91 (1.15, 0.53, 0.95, 0.95, 0.33), IV 5.27 (1.38, 0.68, 1.43, 1.35, 0.43). Carapace mainly red-brown, covered with thin setae. Legs mainly red-brown, with four and two pairs of ventral spines on tibiae and metatarsi I. Dorsum of abdomen dark brown, with pair of longitudinal, anterolateral, pale setal stripes and pair of oblique, median, pale setal patches; venter pale, with broad, central dark brown patch.

***Palp*** (Fig. 33A–E): femur length/width ratio ca 2.1; patella ~ 1.6× longer than wide in retrolateral view; tibia short, with sub-triangular dorsal apophysis (DTA) and slender, S-shaped retrolateral apophysis (RTA); cymbium ~ 1.8× longer than wide, with prolateral ridged portion (CR), blunt retrolateral apophysis (RCA) and tapered baso-dorsal apophysis (DCA) with pointed end; tegulum swollen; median apophysis (MA) large, broadened and forming mesal ridge, with base, elongate-oval lamellar branch extended ventrally; embolus (E) forming half-round disc at base, then tapered and curved into rather blunt tip.

**Female** (Fig. 34A–D, G). Total length 4.88. Carapace 2.02 long, 1.39 wide. Abdomen 2.72 long, 1.47 wide. Eye sizes and inter-distances: AME 0.50, ALE 0.28, PLE 0.26, AERW 1.51, PERW 1.49, EFL 1.21. Legs: I 4.83 (1.50, 1.23, 1.25, 0.55, 0.30), II 3.63 (1.10, 0.55, 0.90, 0.78, 0.30), III 3.94 (1.13, 0.53, 0.95, 0.98, 0.35), IV 5.40 (1.45, 0.70, 1.40, 1.40, 0.45). Habitus (Fig. 34G) similar to that of male except paler and with much shallow similar patterns.

***Epigyne*** (Fig. 34A–D) slightly longer than wide, with broad posterior fold (F) ~ 1/3 atrial width; atrium (At) crinkly, occupied anterior 3/5 and separated by narrow septum (Se), with pair of lateral ridges (AR); copulatory openings (CO) invisible; copulatory ducts (CD) strongly curved at proximal, and connected to inner portions of spermathecae, with bar-shaped, terminal accessory glands (AG); spermathecae (S) touching each other, with spherical inner portions and transversely extended, elongate-oval outside portions.

##### Distribution.

Known only from the type locality in Guangxi, China (Fig. 47).

#### ﻿ Synagelides yidei sp. nov.

Taxon classificationAnimaliaAraneaeSalticidae

A60B6CC1-5E17-55CE-9306-C5692CE5D6BA

https://zoobank.org/D9A6BA46-20F4-458E-950E-7231D9986C9F

Figs 35 , 36 , 48

##### Type material.

***Holotype*** ♂ (TRU-JS 0792), China: • Guangxi Zhuang Autonomous Region, Laibing City, Jinxiu Yao Autonomous County, Yinshan Park (24°10.07'N, 110°14.48'E, ca 1310 m), 8.XI.2021, A.L. He et al. leg. ***Paratypes*** • 1 ♂ (TRU-JS 0793), same data as for holotype; • 1 ♂ (TRU-JS 0794), Shengtangshan Scenic Area (23°58.05'N, 110°6.53'E, ca 1520 m), 11.X.2021, A.L. He et al. leg.

##### Etymology.

The specific name is after Mr. Zhang Yide, who is one of the famous Shu Generals in the Three Kingdoms of ancient China; noun (name) in the genitive case.

##### Diagnosis.

**Figure 35.**
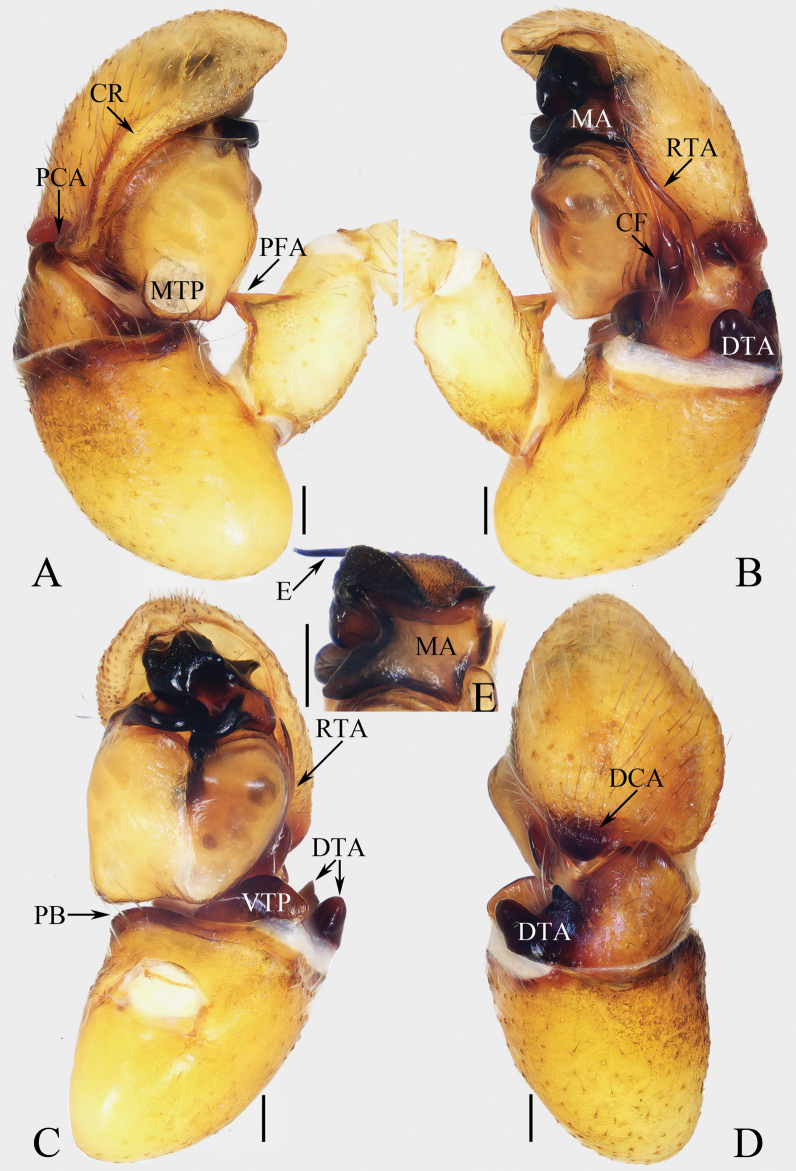
Male palp of *Synagelidesyidei* sp. nov. **A–D** holotype and **E** paratype (TRU-JS 0793) **A** prolateral **B** retrolateral **C** ventral **D** dorsal **E** embolus and median apophysis, retrolateral. Abbreviations: CF cymbial flange; CR prolateral cymbial ridge; DCA dorsal cymbial apophysis; DTA dorsal tibial apophysis; E embolus; MA median apophysis; MTP membranous tegular peak; PB patellar bump of male palp; PCA prolateral cymbial apophysis; PFA prolateral femoral apophysis; RTA retrolateral tibial apophysis; VTP ventral tibial process. Scale bars: 0.1 mm.

*Synagelidesyidei* sp. nov. can be easily distinguished from other known male congeners by the bifurcated dorsal tibial apophysis (DTA) (Fig. 35B–D) vs absent or not bifurcated in congeners (see Metzner 2024).

##### Description.

**Figure 36.**
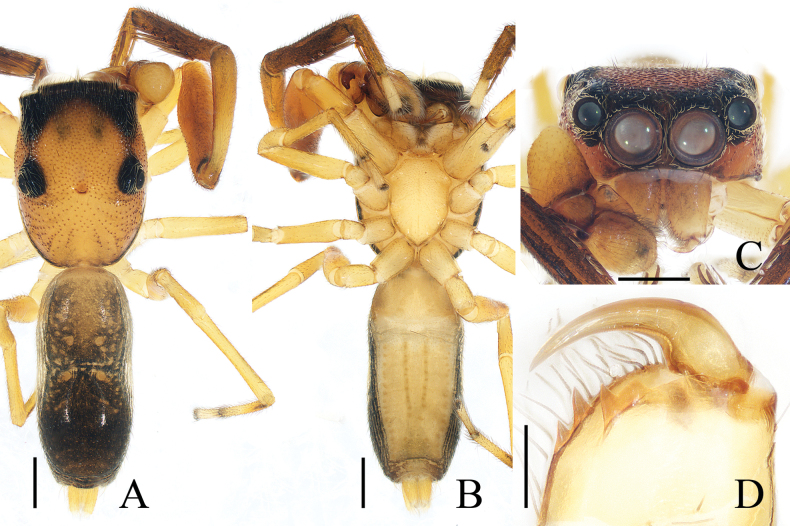
*Synagelidesyidei* sp. nov., holotype **A** habitus, dorsal **B** ditto, ventral **C** carapace, frontal **D** chelicera, posterior. Scale bars: 0.5 mm (**A–C**); 0.1 mm (**D**).

**Male** (Figs 35, 36). Total length 3.93. Carapace 1.76 long, 1.34 wide. Abdomen 2.23 long, 0.95 wide. Eye sizes and interdistances: AME 0.42, ALE 0.23, PLE 0.21, AERW 1.18, PERW 1.30, EFL 0.97. Legs: I 7.13 (2.25, 2.05, 1.63, 0.75, 0.45), II 3.51 (1.03, 0.55, 0.88, 0.70, 0.35), III 3.57 (0.98, 0.53, 0.88, 0.83, 0.35), IV 4.79 (1.28, 0.70, 1.30, 1.13, 0.38). Carapace mainly yellow, with pair of indistinct dark patches anteriorly on square cephalon. Legs slender, bear four and two pairs of ventral spines on tibiae and metatarsi I. Abdomen slightly constricted medially, dorsum dark brown posteriorly, with two pairs of median, yellow muscle depressions; venter pale, with pair of central, dotted lines.

***Palp*** (Fig. 35A–E): femur length/width ratio ca 1.68; patella ~ 1.5× longer than wide in retrolateral view; dorsal tibial apophysis (DTA) bifurcated with two short, blunt rami; retrolateral tibial apophysis (RTA) tapered, slender, > 1/2 cymbial length, and pointed apically; cymbium with prolateral ridged portion (CR), strongly sclerotized, blunt baso-dorsal apophysis (DCA) and prolateral apophysis (PCA); tegulum swollen; median apophysis (MA) irregular, retrolateral to embolus; embolus (E) forming disc at base, then twisted into blunt end.

**Female.** Unknown.

##### Distribution.

Known only from the type locality in Guangxi, China (Fig. 48).

#### ﻿ Synagelides yunchangi sp. nov.

Taxon classificationAnimaliaAraneaeSalticidae

85925AFF-0564-5362-9AFE-196678E05D3B

https://zoobank.org/0477C6C3-63F4-4281-BBF2-3C23EC7F2F4B

Figs 37 , 38 , 47

##### Type material.

***Holotype*** ♂ (TRU-JS 0795), China: • Guangxi Zhuang Autonomous Region, Laibing City, Jinxiu Yao Autonomous County, Shengtangshan Scenic Area (23°58.05'N, 110°6.53'E, ca 1520 m), 11. X.2021, A.L. He et al. leg. ***Paratypes*** • 4 ♂ 2 ♀ (TRU-JS 0796–0801), same data as for holotype.

##### Etymology.

The specific name is after Mr. Guan Yunchang, who is one of the famous Shu Generals in the Three Kingdoms of ancient China; noun (name) in the genitive case.

##### Diagnosis.

**Figure 37.**
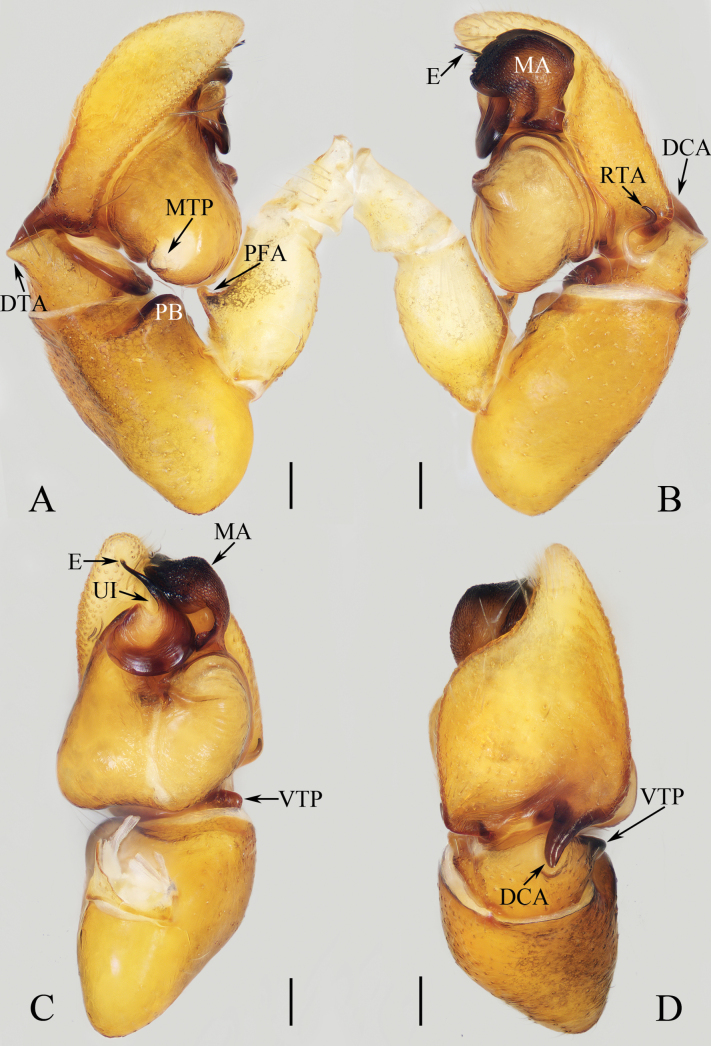
Male palp of *Synagelidesyunchangi* sp. nov., holotype **A** prolateral **B** retrolateral **C** ventral **D** dorsal. Abbreviations: DCA dorsal cymbial apophysis; DTA dorsal tibial apophysis; E embolus; MA median apophysis; MTP membranous tegular peak; PB patellar bump of male palp; PFA prolateral femoral apophysis; RTA retrolateral tibial apophysis; UI U-shaped incision of embolic disc; VTP ventral tibial process. Scale bars: 0.1 mm.

**Figure 38.**
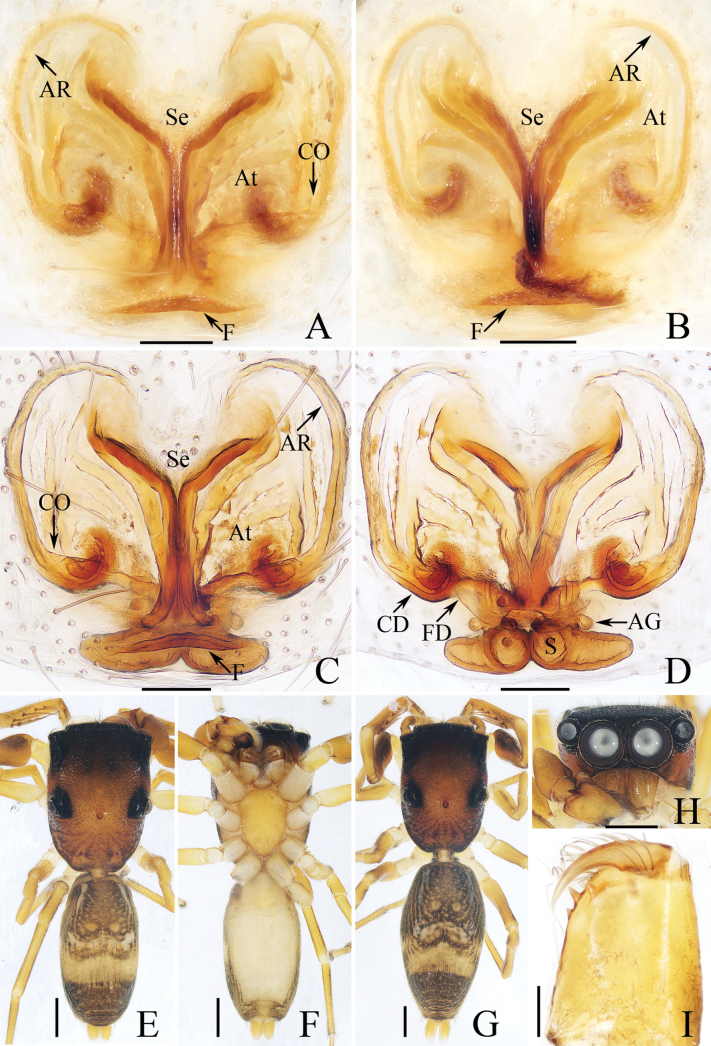
*Synagelidesyunchangi* sp. nov. **E, F, H, I** male holotype **A, C, D, G** female paratype (TRU-JS 0800) **B** female paratype (TRU-JS 0801) **A–C** epigyne, ventral **D** vulva, dorsal **E, G** habitus, dorsal **F** ditto, ventral **H** carapace, frontal **I** chelicera, posterior. Abbreviations: AG accessory gland; At atrium; AR atrial ridge; CD copulatory duct; CO copulatory opening; F epigynal fold; FD fertilization duct; S spermatheca; Se septum. Scale bars: 0.1 mm (**A–D, I**); 0.5 mm (**E–H**).

*Synagelidesyunchangi* sp. nov. resembles that of *S.gambosus* Xie & Yin, 1991, in having very similar copulatory organs, but can be distinguished by the following: 1) ratio of the constricted portion of median apophysis (MA) to the broadest portion ~ 1/2 in retrolateral view (Fig. 37B) vs ~ 1/3 (Peng 2020: fig. 326d); 2) presence of an U-shaped incision (UI) on the anterior margin of embolic disc (Fig. 37C) vs very shallow, near C-shaped incision (Peng 2020: fig. 326b); 3) septum (Se) almost Y-shaped, and ~ 3/5 of atrial width (Fig. 38A–C) vs approximately T-shaped, and > 4/5 of atrial width (Peng 2020: fig. 326f); 4) atrial ridge (AR) approximately auricle-shaped (Fig. 38A–C) vs approximately L-shaped (Peng 2020: fig. 326f); 5) presence of a pair of anterolateral, pale stripes on dorsum of abdomen (Fig. 36E, G) vs pair of round spots (Peng 2020: fig. 326a).

##### Description.

**Male** (Figs 37, 38E, F, H, I). Total length 3.87. Carapace 1.85 long 1.34 wide. Abdomen 1.96 long, 1.05 wide. Eye sizes and interdistances: AME 0.45, ALE 0.25, PLE 0.21, AERW 1.36, PERW 1.40, EFL 1.11. Legs: I 4.41 (1.38, 1.15, 1.10, 0.50, 0.28), II 2.94 (0.88, 0.45, 0.68, 0.63, 0.30), III 3.19 (0.93, 0.45, 0.73, 0.78, 0.30), IV 4.19 (1.15, 0.50, 1.13, 1.08, 0.33). Carapace red-brown, with longitudinal, anteromedian, paired, dark patches on cephalon. Legs slender, with four and two pairs of ventral spines on tibiae and patellae I. Dorsum of abdomen brown at anterior half and dark posteriorly, with pair of anterolateral pale stripes followed by paired yellow spots and transverse, pale patches; venter pale, without patterns.

***Palp*** (Fig. 37A–D): femur length/width ratio ca 1.79; patella ~ 1.8× longer than wide; tibia ~ 1/3 patellar length, with slender, S-shaped retrolateral apophysis (RTA) and sub-triangular dorsal apophysis (DTA); cymbium ~ 1.6× longer than wide, with tapered, spine-shaped baso-dorsal apophysis (DCA); tegulum swollen; median apophysis (MA) irregular, broadened and swollen distally; embolus (E) flat and forming round disc at base, and then acutely narrowed to whip-shaped portion.

**Female** (Fig. 38A–D, G). Total length 4.77. Carapace 2.03 long, 1.47 wide. Abdomen 2.67 long, 1.41 wide. Eye sizes and inter-distances: AME 0.50, ALE 0.27, PLE 0.24, AERW 1.50, PERW 1.48, EFL 1.20. Legs: I 4.38 (1.33, 1.05, 1.20, 0.50, 0.30), II 3.14 (0.98, 0.48, 0.75, 0.63, 0.30), III 3.44 (1.00, 0.48, 0.78, 0.85, 0.33), IV 4.43 (1.25, 0.50, 1.20, 1.13, 0.35). Habitus (Fig. 38G) similar to that of male except slightly darker.

***Epigyne*** (Fig. 38A–D) almost as long as wide, with transverse, lamellar, posterior fold (F); atrium (At) large, occupies anterior 3/5, separated by Y-shaped septum (Se), with pair of auricle-shaped lateral ridges (AR); copulatory openings (CO) beneath baso-lateral portions of atrial ridges; copulatory ducts (CD) thin, anterior half curved into C-shape, posterior half posteriorly descending with short, transversely extending accessory glands (AG) ; spermathecae touched, with spherical inner portions.

##### Distribution.

Known only from the type locality in Guangxi, China (Fig. 47).

#### ﻿ Synagelides zilongi sp. nov.

Taxon classificationAnimaliaAraneaeSalticidae

CFEA074E-7F7C-5ABB-8785-4D63340008C1

https://zoobank.org/80BEA892-2AA3-4BB4-8A21-1DE32F673C9A

Figs 39 , 40 , 48

##### Type Material.

***Holotype*** ♂ (TRU-JS 0802), China: • Yunnan Province, Wenshan City, Wenshan National Nature Reserve, Bozhushan (23°22.19'N, 103°55.17'E, ca 2730 m), 14.V.2024, C. Wang et al.leg. ***Paratypes*** • 1 ♂ 2 ♀ (TRU-JS 0803–0805), same data as for holotype.

##### Etymology.

The specific name is after Mr. Zhao Zilong, who is one of the famous Shu Generals in the Three Kingdoms of ancient China; noun (name) in the genitive case.

##### Diagnosis.

**Figure 39.**
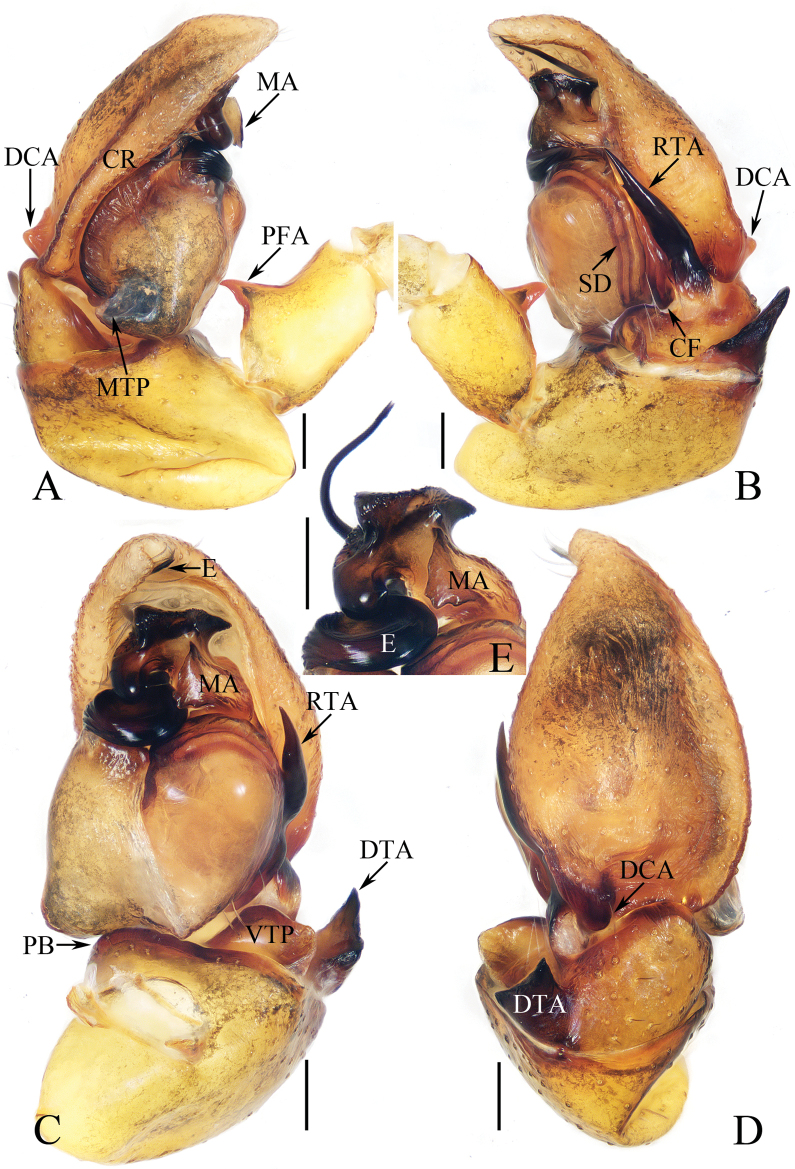
Male palp of *Synagelideszilongi* sp. nov. **A–D** holotype and **E** paratype (TRU-JS 0803) **A** prolateral **B** retrolateral **C** ventral **D** dorsal **E** embolus and median apophysis, ventral. Abbreviations: CF cymbial flange; CR prolateral cymbial ridge; DCA dorsal cymbial apophysis; DTA dorsal tibial apophysis; E embolus; MA median apophysis; MTP membranous tegular peak; PB patellar bump of male palp; PFA prolateral femoral apophysis; RTA retrolateral tibial apophysis; SD sperm duct; VTP ventral tibial process. Scale bars: 0.1 mm.

**Figure 40.**
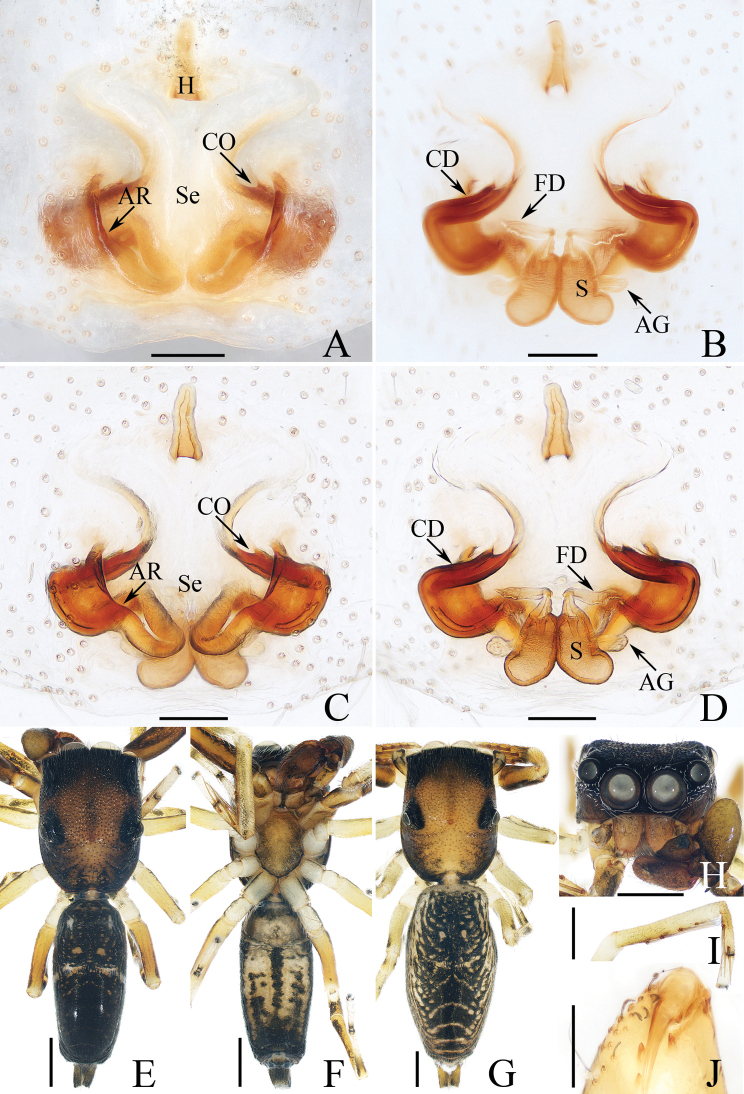
*Synagelideszilongi* sp. nov. **E, F, H–J** male holotype and **A–D, G** female paratype (TRU-JS 0804) **A, C** epigyne, ventral **B, D** vulva, dorsal **E, G** habitus, dorsal **F** ditto, ventral **H** carapace, frontal **I** tibia and metatarsi I, prolateral **J** chelicera, ventral. Abbreviations: AG accessory gland; AR atrial ridge; CD copulatory duct; CO copulatory opening; FD fertilization duct; H epigynal hood; S spermatheca; Se septum. Scale bars: 0.1 mm (**A–D, J**); 0.5 mm (**E–I**).

*Synagelideszilongi* sp. nov. resembles that of *S.jingzhao* Yang, Zhu & Song, 2007 in the habitus and general shape of copulatory organs, but can be easily distinguished by the following: 1) retrolateral tibial apophysis (RTA) ~ 1/2 of cymbial length in retrolateral view (Fig. 39B) vs ~ 3/5 of cymbial length (Yang et al. 2007: fig. 1F); 2) epigynal hood (H) ~ 3× longer than wide (Fig. 40A–D) vs just slightly longer than wide (Yang et al. 2007: fig. 1B); 3) presence of four pairs of ventral spines on tibiae I (Fig. 40I) vs five pairs (see the description in Yang et al. 2007: 1).

##### Description.

**Male** (Figs 39, 40E, F, H–J). Total length 3.18. Carapace 1.43 long, 1.09 wide. Abdomen 1.72 long, 0.78 wide. Eye sizes and inter-distances: AME 0.34, ALE 0.19, PLE 0.18, AERW 1.02, PERW 1.11, EFL 0.83. Legs: I 4.54 (1.38, 1.20, 1.13, 0.50, 0.33), II 2.53 (0.75, 0.40, 0.60, 0.50, 0.28), III 2.59 (0.75, 0.38, 0.60, 0.58, 0.28), IV3.49 (0.95, 0.48, 0.93, 0.83, 0.30). Carapace yellow-brown to dark, with longitudinal, dark, thin stripe centrally on cephalon. Legs slender, with four and two pairs of ventral spines on patellae and metatarsi I. Dorsum of abdomen dark, with pair of anterolateral, pale dots followed by paired, dark yellow dots, and inconsistent, transverse, white stripes bearing sparse white setae medially; venter pale, mingled with dark, with longitudinal, dark stripe extending from epigastric groove to posterior 1/3.

***Palp*** (Fig. 39A–E): femur length/width ratio ca 1.67; patella ~ 1.5× longer than wide; tibia ~ 1/3 patellar length; dorsal tibial apophysis (DTA) strongly sclerotized, directed towards ca 1:30 o’clock position apically in retrolateral view; retrolateral tibial apophysis (RTA) tapered into pointed tip reaches base of median apophysis (MA), with small, spinous ramus located on ventral margin of anterior 1/3; cymbium with prolateral ridged portion (CR), and blunt baso-dorsal apophysis (DCA); tegulum swollen; median apophysis (MA) irregular, retrolateral to embolus; embolus (E) broadened at base, twisted into blunt end.

**Female** (Fig. 40A–D, G). Total length 4.45. Carapace 1.88 long, 1.39 wide. Abdomen 2.58 long, 1.36 wide. Eye sizes and inter-distances: AME 0.42, ALE 0.24, PLE 0.21, AERW 1.29, PERW 1.42, EFL 1.01. Legs: I 4.27 (1.28, 0.98, 1.13, 0.55, 0.33), II 2.89 (0.88, 0.45, 0.68, 0.58, 0.30), III 3.07 (0.88, 0.43, 0.73, 0.73, 0.30), IV 4.27 (1.20, 0.58, 1.13, 1.03, 0.33). Habitus (Fig. 40G) similar to that of male except paler and wider abdomen.

***Epigyne*** (Fig. 40A–D) ~ 1.15× longer than wide, with tube-shaped anterior hood (H) ~ 3× longer than wide; atrium (At) oval, with pair of arc-shaped lateral ridges (AR); septum (Se) wide, narrowest medially; copulatory openings (CO) baso-lateral to narrowest portion of septum; copulatory ducts (CD) slender, curved into U-shape at anterior 2/3, with short, bar-shaped accessory glands (AG); spermathecae (S) elongate-oval, touching each other, ~ 1.8× longer than wide.

##### Distribution.

Known only from the type locality in Yunnan, China (Fig. 48).

#### ﻿Genus Yaginumaella

Taxon classificationAnimaliaAraneaeSalticidae

Prószyński, 1979

41D092FB-D241-506E-B08B-2E1888773351

##### Type species.

*Pellenesususudi* Yaginuma, 1972; type locality Hidaka District, Hokkaido, Japan.

##### Comments.

*Yaginumaella*, a member of Plexippini (Maddison 2015), is represented by 17 species restricted in east, south, and southeast Asia (WSC 2024). The genus is relatively poorly studied because it has never been widely revised, and more than 35% of species are known only from a single sex (WSC 2024). Moreover, the relationship between the genus and *Ptocasius* Simon, 1885 remains controversial, and thus the generic position of many related species cannot be ultimately confirmed (Logunov 2024; Wang et al. 2024).

#### ﻿ Yaginumaella daweishan sp. nov.

Taxon classificationAnimaliaAraneaeSalticidae

1B3995C8-947A-542C-BC90-C9AF9B155CE7

https://zoobank.org/06D6603D-1E63-4D32-91A7-05A9AEE538A8

Figs 41 , 42 , 47

##### Type material.

***Holotype*** ♂ (TRU-JS 0806), China: • Yunnan Province, Pingbian Miao Autonomous County, Daweishan National Nature Reserve (22°54.81'N, 103°42.02'E, ca 2040 m), 15.V.2024, C. Wang et al. leg. ***Paratypes*** • 2 ♂ 6 ♀ (TRU-JS 0807–0814), same data as for holotype.

##### Etymology.

The specific name refers to type locality; noun (name) in apposition.

##### Diagnosis.

**Figure 41.**
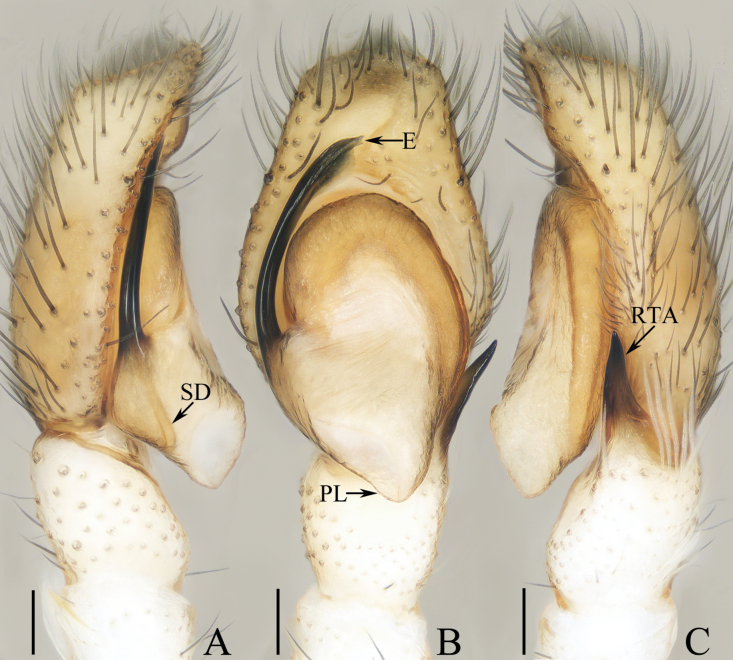
Male palp of *Yaginumaelladaweishan* sp. nov., holotype **A** prolateral **B** ventral **C** retrolateral. Abbreviations: E embolus; PL posterior tegular lobe; RTA retrolateral tibial apophysis; SD sperm duct. Scale bars: 0.1 mm.

**Figure 42.**
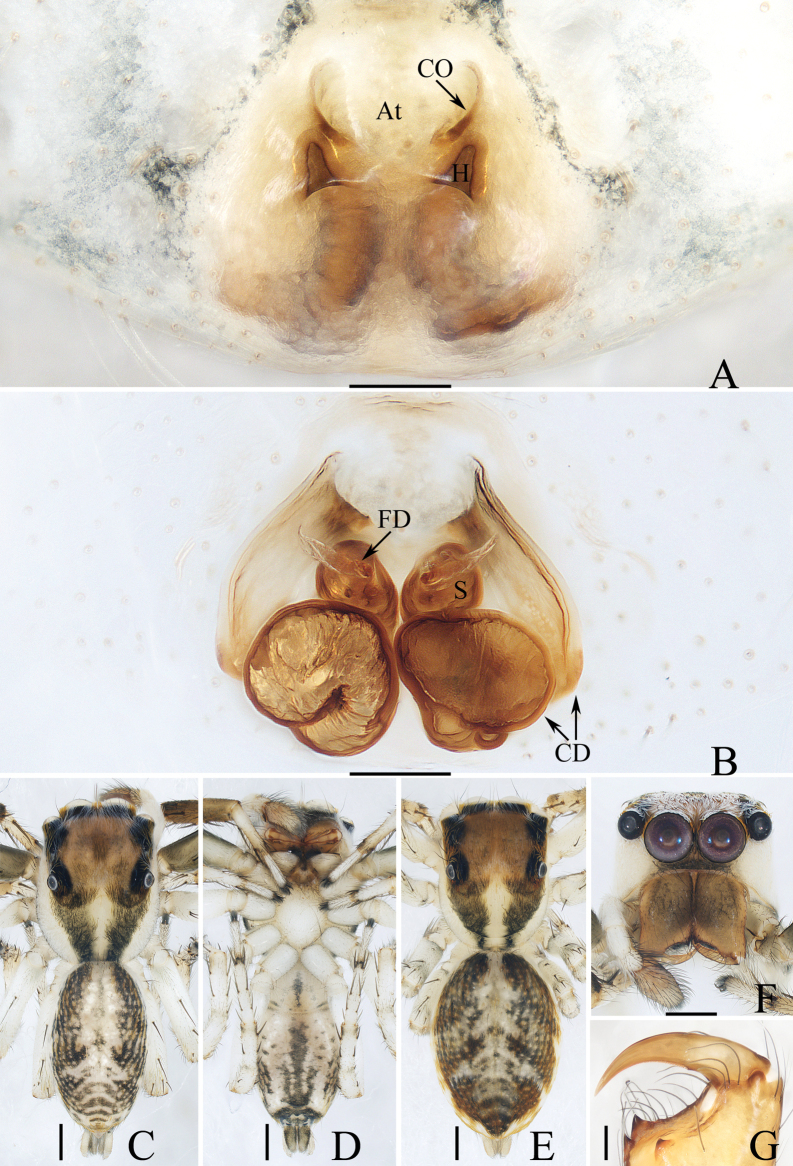
*Yaginumaelladaweishan* sp. nov. **C, D, F, G** male holotype and **A, B, E** female paratype (TRU-JS 0809) **A** epigyne, ventral **B** vulva, dorsal **C**, **E** habitus, dorsal **D** ditto, ventral **F** carapace, frontal **G** chelicera, posterior. Abbreviations: At atrium; CD copulatory duct; CO copulatory opening; FD fertilization duct; H epigynal hood; S spermatheca. Scale bars: 0.1 mm (**A, B, G**); 0.5 mm (**C–F**).

*Yaginumaelladaweishan* sp. nov. resembles that of *Y.ususudi* (Yaginuma, 1972) in the general shape of copulatory organs, but can be distinguished by the following: 1) embolus (E) slightly curved, and with terminal broadened part (Fig. 41B) vs curved into a C-shape and without similar broadened part (Bohdanowicz and Prószyński 1987: fig. 307); 2) retrolateral tibial apophysis (RTA) tapered into a pointed tip in retrolateral view (Fig. 41C) vs almost equal in width from the base to the distal portion, and with a blunt tip (Bohdanowicz and Prószyński 1987: fig. 308); 3) epigynal hood (H) slightly longer than wide (Fig. 42A) vs > 2.5× wider than long (Bohdanowicz and Prószyński 1987: fig. 309).

##### Description.

**Male** (Figs 41, 42C, D, F, G). Total length 4.46. Carapace 2.18 long, 1.75 wide. Abdomen 2.39 long, 1.36 wide. Eye sizes and interdistances: AME 0.51, ALE 0.30, PLE 0.28, AERW 1.59, PERW 1.54, EFL 0.98. Legs: I 4.76 (1.55, 0.83, 0.88, 0.90, 0.60), II 4.20 (1.25, 0.70, 1.00, 0.75, 0.50), III 4.82 (1.50, 0.63, 1.08, 1.08, 0.53), IV 5.02 (1.55, 0.63, 1.13, 1.18, 0.53). Carapace sub-square, with pair of bilateral pale setal bands and pair of longitudinal, dark brown stripes separated by central pale-yellow stripe on thoracic part. Legs pale except legs I darker, with three and two pairs of ventral spines on tibiae and metatarsi I. Dorsum of abdomen dark brown, with longitudinal, central pale band bearing sparse sliver spots, followed by several arc-shaped transverse pale stripes; venter pale, with central, longitudinal, irregular dark patch.

***Palp*** (Fig. 41A–C): femur length/width ratio ca 3.4; patella ~ 1.4× longer than wide in retrolateral view; tibia as long as retrolateral tibial apophysis (RTA); retrolateral tibial apophysis tapering to pointed tip directed anteriorly; cymbium ~ 1.6× longer than wide; tegulum ca 1.5× longer than wide, with posteriorly extended posterior lobe (PL); embolus (E) originating at ca 9 o’clock position, slightly curved, and terminating at 12 o’clock position, with broadened terminal part.

**Female** (Fig. 42A, B, E). Total length 4.63. Carapace 1.77 long, 1.40 wide. Abdomen 2.80 long, 1.97 wide. Eye sizes and interdistances: AME 0.47, ALE 0.23, PLE 0.20, AERW 1.30, PERW 1.25, EFL 0.90. Legs: I 4.23 (1.30, 0.75, 1.00, 0.70, 0.48), II 3.94 (1.20, 0.68, 0.93, 0.65, 0.48), III 4.84 (1.50, 0.68, 1.05, 1.08, 0.53), IV 5.02 (1.55, 0.63, 1.13, 1.18, 0.53). Habitus (Fig. 42E) similar to that of male except darker.

***Epigyne*** (Fig. 42A, B) approximately as long as wide, with pair of hoods (H) posteriorly to copulatory openings (CO), and ~ 1.5× longer than wide; atrium (At) oval, located anteriorly; copulatory ducts (CD) broad, forming complicated path; spermathecae (S) almost oval.

##### Distribution.

Known only from the type locality in Yunnan, China (Fig. 47).

#### ﻿ Yaginumaella moinba sp. nov.

Taxon classificationAnimaliaAraneaeSalticidae

8CCF4E61-9721-5C59-B23B-BA1FBAE00E09

https://zoobank.org/0706CACB-A27D-4E22-A3C9-DEA584C425BB

Figs 43 , 44 , 47

##### Type material.

***Holotype*** ♂ (TRU-JS 0815), China: • Xizang Autonomous Region, Medog County, Beibeng Township, Deergong Village, Yarlung Zangbo National Nature Reserve (29°10.84'N, 95°8.67'E, ca 1670 m), 25.V.2024, X.Q. Mi et al. leg. ***Paratypes*** • 2 ♂ 1 ♀ (TRU-JS 0816–0818), same data as for holotype.

##### Etymology.

The specific name is after the Mionba ethnic group, one of the two significant national minorities in Medog; noun in apposition.

##### Diagnosis.

**Figure 43.**
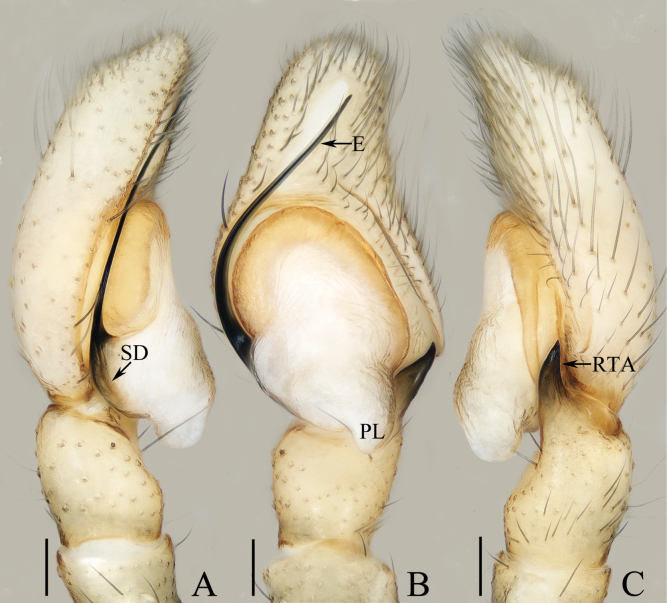
Male palp of *Yaginumaellamoinba* sp. nov., holotype **A** prolateral **B** ventral **C** retrolateral. Abbreviations: E embolus; PL posterior tegular lobe; RTA retrolateral tibial apophysis; SD sperm duct. Scale bars: 0.1 mm.

**Figure 44.**
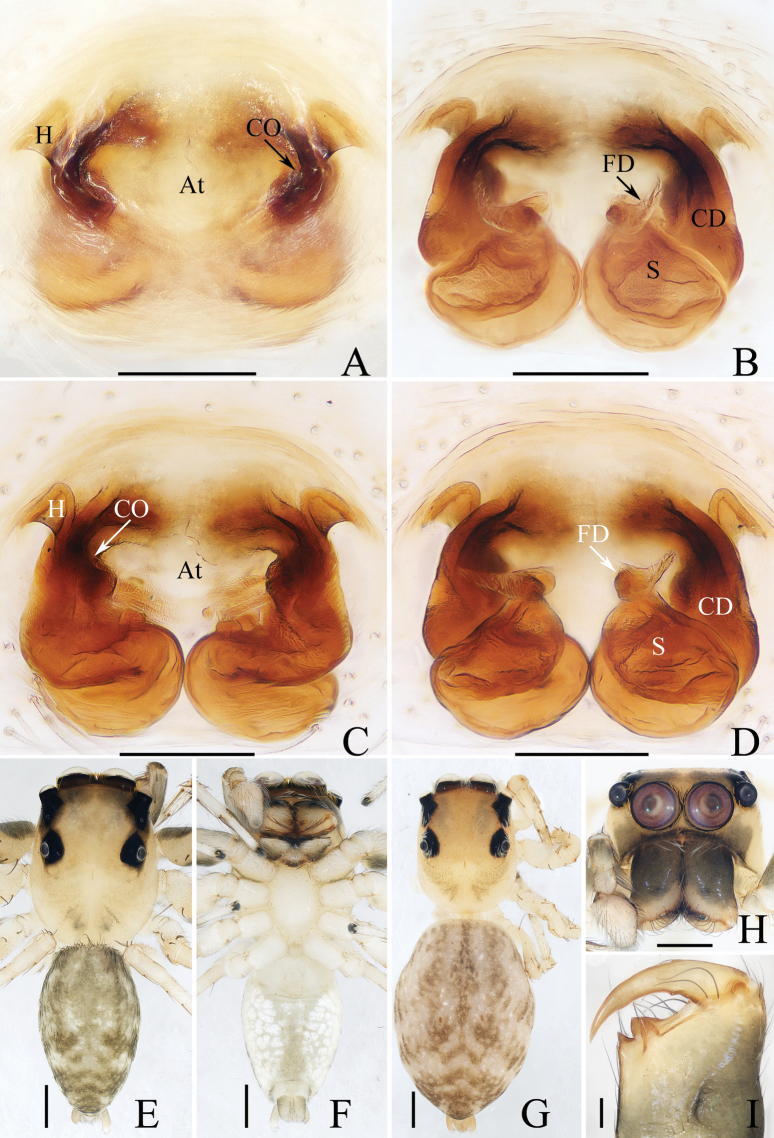
*Yaginumaellamoinba* sp. nov. **E, F, H, I** male holotype and **A–D, G** female paratype (TRU-JS 0818) **A, C** epigyne, ventral **B, D** vulva, dorsal **E, G** habitus, dorsal **F** ditto, ventral **H** carapace, frontal **I** chelicera, posterior. Abbreviations: At atrium; CD copulatory duct; CO copulatory opening; FD fertilization duct; H epigynal hood; S spermatheca. Scale bars: 0.1 mm (**A–D, I**); 0.5 mm (**E–H**).

The male of *Yaginumaellamoinba* sp. nov. resembles that of *Y.curvata* Li, Liu & Peng, 2024 in the general shape of palpal structure, but can be distinguished by the following: 1) embolus arising at ca 8:30 o’clock position (Fig. 43A, B) vs ca 6:30 o’clock position (Li et al. 2024: fig. 4A); 2) retrolateral tibial apophysis (RTA) apically directed towards ca 12:30 o’clock position in retrolateral view (Fig. 43C) vs ca 1: 30 o’clock (Li et al. 2024: fig. 4B). The female of this species resembles that of *Y.pingbian* sp. nov. in having anterolaterally located, bell-shaped epigynal hood (H), but can be easily distinguished by the copulatory ducts, which are extending into an approximate S-shape (Fig. 44B–D) vs extending into approximate L-shape at beginning (Fig. 46B).

##### Description.

**Male** (Figs 43, 44E, F, H, I). Total length 3.94. Carapace 1.94 long, 1.54 wide. Abdomen 2.00 long, 1.23 wide. Eye sizes and interdistances: AME 0.46, ALE 0.24, PLE 0.20, AERW 1.36, PERW 1.29, EFL 0.89. Legs: I 4.18 (1.20, 0.68, 1.10, 0.70, 0.50), II 3.86 (1.20, 0.63, 0.95, 0.63, 0.45), III 4.39 (1.28, 0.63, 1.03, 0.95, 0.50), IV 4.59 (1.28, 0.55, 1.13, 1.13, 0.50). Carapace pale yellow except baso-lateral part of face and area behind AMEs brown. Legs pale yellow, with dark brown patches on femora I, and three and two pairs of ventral spines on tibiae and metatarsi I. Dorsum of abdomen with irregular green-brown patches and sliver spots; venter grey centrally and covered with dense sliver spots laterally.

***Palp*** (Fig. 43A–C): femur length/width ratio ca 3.5; patella ~ 1.45× longer than tibia; tibia ~ 1.3× longer than wide in retrolateral view, with tapered retrolateral apophysis (RTA) shorter than tibia, slightly curved medially and pointed apically; cymbium ~ 1.5× longer than wide in ventral view; tegulum almost oval, slightly swollen medio-posteriorly, with tapered, somewhat curved posterior lobe (PL); embolus (E) originating at ca 8:30 o’clock position, curved clockwise along the tegulum at anterior half, and terminating at ca 1 o’clock position.

**Female** (Fig. 44A–D, G). Total length 4.63. Carapace 1.77 long, 1.40 wide. Abdomen 2.80 long, 1.97 wide. Eye sizes and interdistances: AME 0.47, ALE 0.23, PLE 0.20, AERW 1.30, PERW 1.25, EFL 0.90. Legs: I 3.23 (0.90, 0.65, 0.75, 0.50, 0.43), II 3.07 (0.88, 0.60, 0.68, 0.48, 0.43), III 3.66 (1.08, 0.60, 0.75, 0.75, 0.48), IV 3.86 (1.13, 0.55, 0.85, 0.85, 0.48). Habitus (Fig. 44G) similar to that of male except dorsum of abdomen brown.

***Epigyne*** (Fig. 44A–D) ~ 1.3× wider than long, with pair of anterior hoods (H) lateral to transversely oval atrium (At) and copulatory openings (CO); copulatory ducts (CD) curved into ca S-shape; spermathecae (S) oval, separated from each other by ~ 1/4 of their width.

##### Distribution.

Known only from the type locality in Xizang, China (Fig. 47).

#### ﻿ Yaginumaella pingbian sp. nov.

Taxon classificationAnimaliaAraneaeSalticidae

92DAAA87-A0E7-57C7-A6C9-E49714BE0298

https://zoobank.org/5A9A8D0B-A854-402D-AFC6-5D3045DC751B

Figs 45 , 46 , 47

##### Type material.

***Holotype*** ♂ (TRU-JS 0819), China: • Yunnan Province, Pingbian Miao Autonomous County, Daweishan National Nature Reserve (22°54.81'N, 103°42.02'E, ca 2040 m), 15.V.2024, C. Wang et al. leg. ***Paratypes*** • 3 ♀ (TRU-JS 0820–0822), same data as for holotype.

##### Etymology.

The species name refers to the type locality: Pingbian Miao Autonomous County; noun in apposition.

##### Diagnosis.

**Figure 45.**
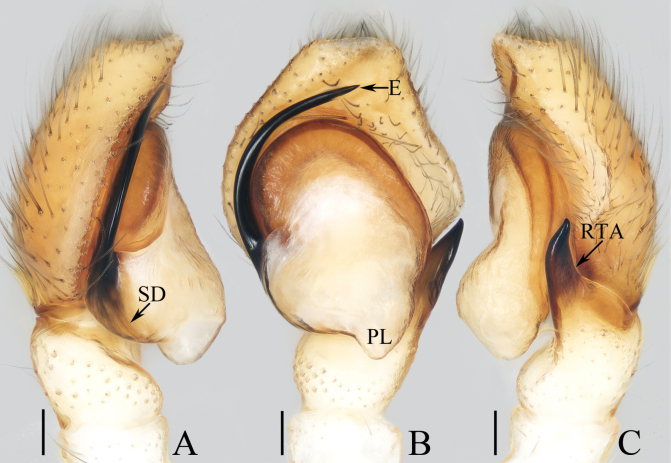
Male palp of *Yaginumaellapingbian* sp. nov., holotype **A** prolateral **B** ventral **C** retrolateral. Abbreviations: E embolus; PL posterior tegular lobe; RTA retrolateral tibial apophysis; SD sperm duct. Scale bars: 0.1 mm.

**Figure 46.**
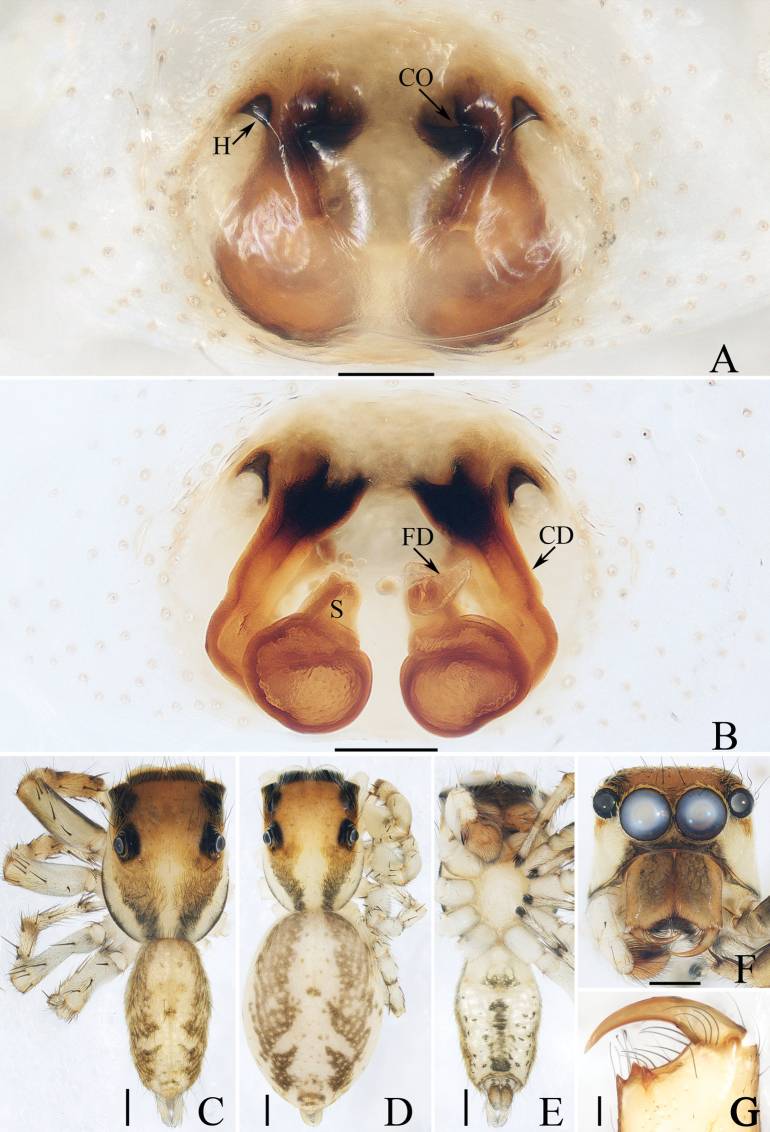
*Yaginumaellapingbian* sp. nov. **C, E–G** male holotype and **A, B, D** female paratype (TRU-JS 0820) **A** epigyne, ventral **B** vulva, dorsal **C, D** habitus, dorsal **E** ditto, ventral **F** carapace, frontal **G** chelicera, posterior. Abbreviations: CD copulatory duct; CO copulatory opening; FD fertilization duct; H epigynal hood; S spermatheca. Scale bars: 0.1 mm (**A, B, G**); 0.5 mm (**C–F**).

*Yaginumaellapingbian* sp. nov. resembles that of *Y.erlang* Wang, Mi & Li, 2024 in its habitus and general shape of copulatory organs, but can be easily distinguished by the following: 1) male palpal tibia approximately~ as long as wide in retrolateral view (Fig. 45C) vs wider than long (Wang et al. 2024: fig. 20C); 2) central longitudinal band on thoracic part in male ~ 1/4 of carapace width (Fig. 46C) vs < 1/5 (Wang et al. 2024: fig. 21C); 3) epigynal hood (H) strongly sclerotized, opened towards 6: 30 o’clock position (Fig. 46A) vs weakly sclerotized, opened towards ca 7:30 o’clock position (Wang et al. 2024: fig. 21A). The female also somewhat resembles that of *Y.moinba* sp. nov., but can be easily distinguished by the copulatory ducts (CO), which are extending into L-shape at beginning (Fig. 46B) vs approximately S-shape (Fig. 44B–D).

##### Description.

**Male** (Figs 45, 46C, E–G). Total length 4.37. Carapace 2.31 long, 1.76 wide. Abdomen 2.20 long, 1.19 wide. Eye sizes and interdistances: AME 0.52, ALE 0.30, PLE 0.28, AERW 1.63, PERW 1.56, EFL 0.98. Legs: I 4.79 (1.50, 0.78, 1.13, 0.85, 0.53), II 4.26 (1.30, 0.75, 0.95, 0.73, 0.53), III 4.56 (1.43, 0.70, 1.00, 0.95, 0.48), IV 5.08 (1.50, 0.65, 1.18, 1.20, 0.55). Carapace mainly orange-yellow, covered with dark and golden setae, with pair of lateral pale bands bearing pale setae and longitudinal, central pale stripe extending across thoracic part. Legs pale, mingled with orange-brown, with lateral dark brown stripes on femora I, and three and two pairs of ventral spines on tibiae and metatarsi I. Dorsum of abdomen brown, with longitudinal, central, broad pale band extending across whole surface and bifurcated at posterior 1/3; venter pale, with longitudinal, central inconsecutive green-brown stripes.

***Palp*** (Fig. 45A–C): femur length/width ratio ca 3.0; patella ~ 1.15× longer than wide in retrolateral view; tibia almost as long as wide in retrolateral view; retrolateral tibial apophysis (RTA) tapered, longer than tibia, curved distally into pointed tip; cymbium ca 1.2× longer than wide, with almost horizontal tip; tegulum nearly oval, with posteriorly extending posterior lobe (PL), embolus (E) originating at ca 8:30 o’clock position of bub, curved into C-shape.

**Female** (Fig. 46A, B, D). Total length 5.32. Carapace 2.20 long, 1.64 wide. Abdomen 3.24 long, 2.28 wide. Eye sizes and interdistances: AME 0.52, ALE 0.32, PLE 0.28, AERW 1.60, PERW 1.56, EFL 1.04. Legs: I 4.18 (1.25, 0.75, 1.00, 0.68, 0.50), II 3.94 (1.25, 0.70, 0.88, 0.63, 0.48), III 4.89 (1.58, 0.75, 0.93, 1.03, 0.60), IV 5.22 (1.58, 0.70, 1.18, 1.18, 0.58). Habitus (Fig. 46D) similar to that of male.

***Epigyne*** (Fig. 46A, B) ~ 1.3× wider than long, with pair of sub-triangular, anterior hoods (H) lateral to copulatory openings (CO); copulatory ducts (CD) run into L-shape at beginning, and with complex distal curves; spermathecae (S) without distinct border.

##### Distribution.

**Figure 47.**
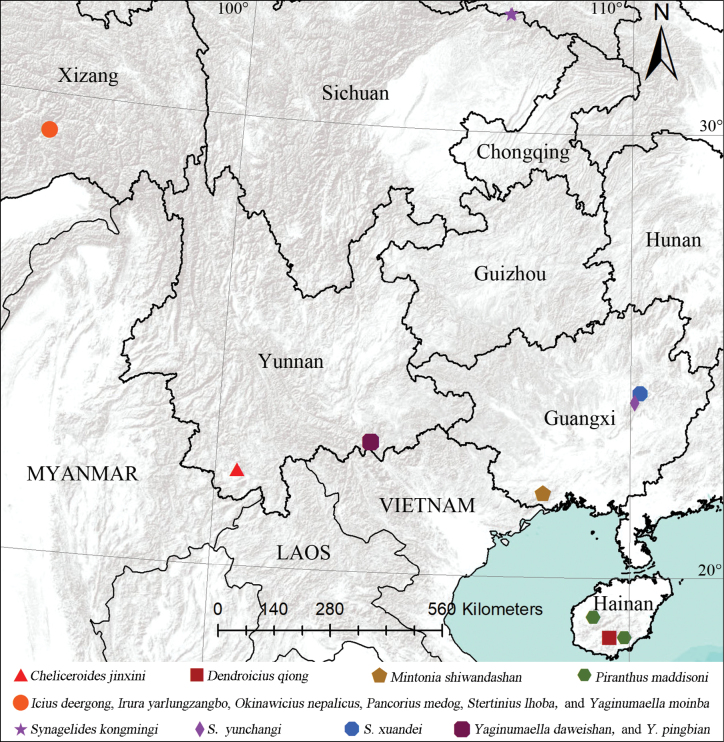
Distributional records of *Cheliceroidesjinxini* sp. nov., *Dendroiciusqiong* sp. nov., *Iciusdeergong* sp. nov., *I.yarlungzangbo* sp. nov., *Mintoniashiwandashan* sp. nov., *Okinawiciusnepalicus* (Andreeva, Hęciak & Prószyński, 1984), *Pancoriusmedog* sp. nov., *Piranthusmaddisoni* sp. nov., *Stertiniuslhoba* sp. nov., *Synagelideskongmingi* sp. nov., *S.yunchangi* sp. nov., *S.xuandei* sp. nov., *Yaginumaelladaweishan* sp. nov., *Y.moinba* sp. nov., and *Y.pingbian* sp. nov.

Known only from the type locality in Yunnan, China (Fig. 47).

**Figure 48.**
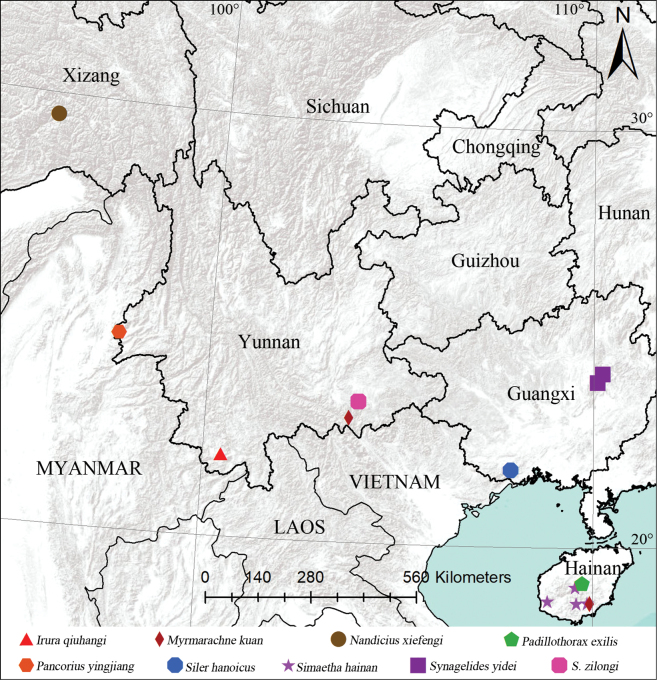
Distributional records of *Iruraqiuhangi* sp. nov., *Myrmarachnekuan* sp. nov., *Nandiciusxiefengi* sp. nov., *Padillothoraxexilis* (Cao & Li, 2016), *Pancoriusyingjiang* sp. nov., *Silerhanoicus* Prószyński, 1985, *Simaethahainan* sp. nov., *Synagelidesyidei* sp. nov., and *S.zilongi* sp. nov.

## Supplementary Material

XML Treatment for Cheliceroides

XML Treatment for Cheliceroides jinxini

XML Treatment for Dendroicius

XML Treatment for Dendroicius qiong

XML Treatment for Icius

XML Treatment for Icius deergong

XML Treatment for Irura

XML Treatment for Irura qiuhangi

XML Treatment for Irura yarlungzangbo

XML Treatment for Mintonia

XML Treatment for Mintonia shiwandashan

XML Treatment for Myrmarachne

XML Treatment for Myrmarachne kuan

XML Treatment for Nandicius

XML Treatment for Nandicius xiefengi

XML Treatment for Okinawicius

XML Treatment for Okinawicius nepalicus

XML Treatment for Padillothorax

XML Treatment for Padillothorax exilis

XML Treatment for Pancorius

XML Treatment for Pancorius medog

XML Treatment for Pancorius yingjiang

XML Treatment for Piranthus

XML Treatment for Piranthus maddisoni

XML Treatment for Siler

XML Treatment for Siler hanoicus

XML Treatment for Simaetha

XML Treatment for Simaetha hainan

XML Treatment for Stertinius

XML Treatment for Stertinius lhoba

XML Treatment for Synagelides

XML Treatment for Synagelides kongmingi

XML Treatment for Synagelides xuandei

XML Treatment for Synagelides yidei

XML Treatment for Synagelides yunchangi

XML Treatment for Synagelides zilongi

XML Treatment for Yaginumaella

XML Treatment for Yaginumaella daweishan

XML Treatment for Yaginumaella moinba

XML Treatment for Yaginumaella pingbian

## ﻿Additional information

### Author ORCIDs

Cheng Wang https://orcid.org/0000-0003-1831-0579

Xiaoqi Mi https://orcid.org/0000-0003-1744-3855

Shuqiang Li https://orcid.org/0000-0002-3290-5416

Xiang Xu https://orcid.org/0000-0001-9485-5373

### Data availability

All of the data that support the findings of this study are available in the main text.
